# Iridium/N-heterocyclic carbene-catalyzed C–H borylation of arenes by diisopropylaminoborane

**DOI:** 10.3762/bjoc.12.65

**Published:** 2016-04-07

**Authors:** Mamoru Tobisu, Takuya Igarashi, Naoto Chatani

**Affiliations:** 1 Center for Atomic and Molecular Technologies, Graduate School of Engineering, Osaka University, Osaka 565-0871, Japan; 2 Department of Applied Chemistry, Faculty of Engineering, Osaka University, Osaka 565-0871, Japan

**Keywords:** boronic acid, C–H borylation, iridium, N-heterocyclic carbene

## Abstract

Catalytic C–H borylation of arenes has been widely used in organic synthesis because it allows the introduction of a versatile boron functionality directly onto simple, unfunctionalized arenes. We report herein the use of diisopropylaminoborane as a boron source in C–H borylation of arenes. An iridium(I) complex with 1,3-dicyclohexylimidazol-2-ylidene is found to efficiently catalyze the borylation of arenes and heteroarenes. The resulting aminoborylated products can be converted to the corresponding boronic acid derivatives simply by treatment with suitable diols or diamines.

## Introduction

**Scheme 1:**
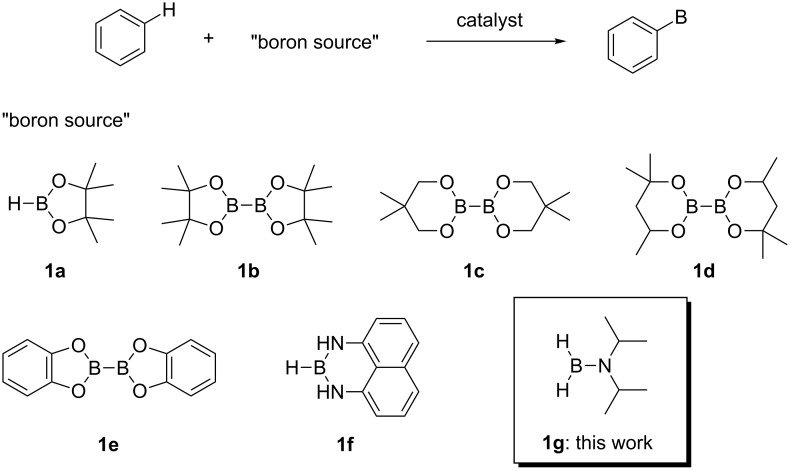
Catalytic C–H borylation of arenes and related reported boron sources.

Catalytic C–H borylation of arenes has become an essential tool in organic synthesis [1]. The eminent features of this methodology include 1) no directing group is needed, allowing the direct functionalization of simple arenes; 2) the regioselectivity is readily predictable based on steric factors; 3) the resulting boryl group is versatile and can be converted into a variety of carbon- or heteroatom-based substituents. An iridium complex in conjunction with 4,4’-di-*tert*-butylbipyridine (dtbpy) developed by Ishiyama, Miyaura and Hartwig has served as the state-of-the-art catalyst for C–H borylation of arenes [2]. In addition to the Ir/dtbpy system, various other catalytic systems have also been developed. For example, base metals such as Fe [3–6], Co [7] and Ni [8–9] have been shown to be viable metal centers for the use as C–H borylation catalysts. We also reported the first use of a Pt catalyst that enables C–H borylation of simple hindered arenes such as mesitylene [10–11]. Metal-free C–H borylation has also been reported [12]. Ligand modification has been used to control the regioselectivity of C–H borylation reactions; for example, in the *ortho*-selective C–H borylation of arenes containing a directing group [13], and to improve the *meta*/*para*-selectivity of monosubstituted benzenes [14–15]. Despite considerable progress in the C–H borylation reaction, the scope of the boryl group that can be introduced is relatively limited (Scheme 1). The most commonly used boron sources are pinacolborane (HBpin, **1a**) and bis(pinacolato)diboron (B_2_pin_2_, **1b**), which form pinacol esters of arylboronic acids. Although the pinacol ester products prepared in these reactions are amenable to a range of transformations, their reactivity is generally lower than that of the corresponding boronic acids. Because of this lower reactivity, several transformations require deprotection of a pinacol ester under oxidative conditions (e.g., NaIO_4_) [16]. Hartwig reported that the Ir/dtbpy system is also able to introduce more reactive neopentyl and hexylene glycolate esters and catecolates using the corresponding diboron reagents **1c**, **1d**, and **1e**, respectively [17–18]. Suginome reported that an Ir/1,2-diphenylphosphinoethane catalyst can promote C–H borylation using 1,8-naphthalenediaminatoborane (**1f**) [19]. The resulting diaminonaphthalene (dan)-protected arylboronic acid is synthetically useful because it can be readily deprotected with a dilute aqueous acid [20].

We envisioned that diisopropylaminoborane (**1g**) [21] could be a useful boron source because the resulting aminoborylated products are sufficiently labile to be converted into various boron derivatives by treatment with protecting groups in a one-pot reaction sequence. The reactivity of **1g** has previously been well-exploited in catalytic borylation of aryl halides [22–27]. Herein, we report the C–H borylation of arenes using **1g** catalyzed by an Ir/N-heterocyclic carbene (NHC) system.

## Results and Discussion

On the basis of a superior reactivity of indoles in several C–H borylation reactions [7–9], we initially examined the borylation of indole **2** with aminoborane **1g** using an iridium catalyst under forcing conditions (140 °C, 15 h). Although all the attempts to isolate an initially formed aminoborylated product **3** were not successful, its formation was confirmed by ^11^B NMR (δ = 40.7 ppm in cyclohexane-*d*_12_). The crude reaction mixture was treated with pinacol and the yield of the product was estimated by ^1^H NMR spectroscopy. Using dtbpy, the common ligand for iridium-catalyzed C–H borylation [2], the reaction failed to give **2-B** under these conditions (Table 1, entry 1). Several mono- and diphosphine ligands were found to be active for the formation of **2-B**, but the best yield was only 21% (Table 1, entries 2–6). Our success in C–H borylation using NHC ligands [8,10] led us to investigate a series of NHC ligands for this process. Among the NHC ligands examined, 1,3-dicyclohexylimidazol-2-ylidene (ICy) [28–33] was found to be most effective, giving **2-B** in 33% yield with a 2-/3-borylation ratio of 88:12 (Table 1, entry 9). It should be noted that [Ir(cod)(ICy)_2_](CF_3_CO_2_) was previously reported to promote C–H borylation of arenes using HBpin [34]. Further optimization using an ICy ligand determined that decreasing the reaction temperature to 110 °C and shortening the reaction time to 4 h markedly improved the yield of **2-B** (72%) with near complete regioselectivity (99:1) (Table 1, entry 12).

**Table 1:** Effect of the ligand on the Ir-catalyzed borylation of **2** with **1g**.^a^

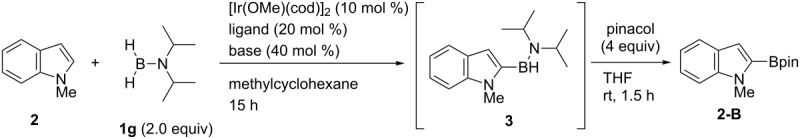					

Entry	Ligand	Base	Temperature [°C]	NMR yield^b^ [%]	2-Isomer/3-Isomer

1	dtbpy	none	140	trace	–
2	PPh_3_	none	140	21	57/43
3	PCy_3_	none	140	3	>99/1
4	dppe	none	140	2	>99/1
5	xantphos	none	140	18	56/44
6	Xphos	none	140	19	71/29
7	IMes·HCl	NaO*t*-Bu	140	5	>99/1
8	IPr·HCl	NaO*t*-Bu	140	3	>99/1
9	ICy·HCl	NaO*t*-Bu	140	33	88/12
10	I*t*-Bu·HCl	NaO*t*-Bu	140	0	–
11	ICy·HCl	NaO*t*-Bu	110	58	95/5
12	ICy·HCl	NaO*t*-Bu	110	72 (65)^c^	99/1

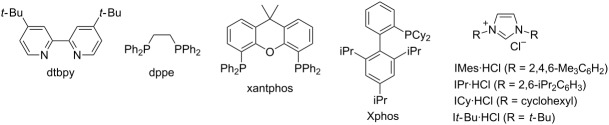					

^a^Reaction conditions: **2** (0.50 mmol), **1g** (1.0 mmol), [Ir(OMe)(cod)]_2_ (0.050 mmol), ligand (0.10 mmol), NaO*t*-Bu (0.20 mmol) in methylcyclohexane (1.0 mL) at 140 °C for 15 h. After treatment with pinacol (2.0 mmol), the borylated product was converted to the corresponding pinacolate. ^b^The yield refers to a combined NMR yield of 2- and 3-borylated products. ^c^Isolated yield.

Having optimized the conditions, we next explored the scope of Ir/ICy-catalyzed borylation of heteroarene substrates using **1g** (Table 2). Functionalized indoles, such as those containing methoxy, fluoro, bromo and chloro substituents, all underwent the borylation to form the corresponding 2-borylated products **4-B**, **5-B**, **6-B** and **7-B**, respectively (Table 2, entries 1–4). When 1,4-dimethylindole (**8**) was used, 2-borylated product **8-B** was formed exclusively with no borylation occurring at the benzylic position (Table 2, entry 5) [8,35–36]. Benzothiophenes readily gave 2-borylated products using our system, as exemplified by the high yields obtained from **9** and **10** (Table 2, entries 6 and 7). Although benzofuran **11** was borylated at the 2-position successfully, the isolated yield was somewhat lower than the yield calculated from the ^1^H NMR data, probably because of the instability of **11-B** during isolation (Table 2, entry 8). Our protocol was able to borylate non-benzofused five-membered heteroarenes. Pyrrole **12** was much less reactive than indoles, and required neat conditions to obtain a modest yield of the borylated product **12-B** (Table 2, entry 9). Thiophene (**13**) afforded a 1.1:1 mixture of 2-borylated and 2,5-diborylated products under our standard conditions (Table 2, entry 10). 2-Substituted thiophenes **14** and **15** and furan **16** were borylated successfully at the 5-positions (Table 2, entries 11–13). Electron-deficient heteroarenes such as pyridine and quinolone failed to form the borylated product under the current conditions.

**Table 2:** Scope of the heteroarene substrates.^a^

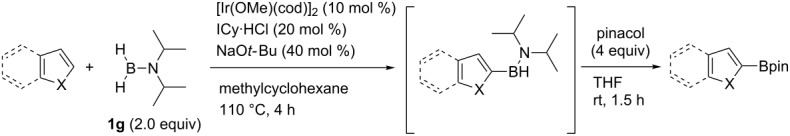			

Entry	Heteroarene	Product	Isolated yield (NMR yield) [%]

1	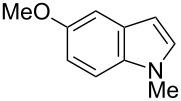 **4**	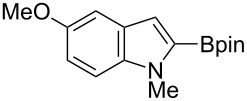 **4-B**	48 (51)
2	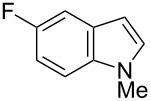 **5**	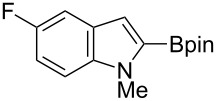 **5-B**	75 (82)
3	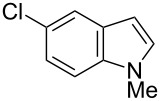 **6**	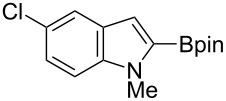 **6-B**	66 (66)
4	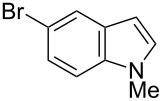 **7**	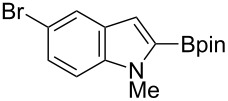 **7-B**	50 (55)^b^
5	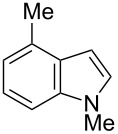 **8**	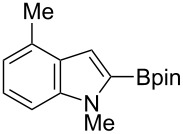 **8-B**	51 (58)
6	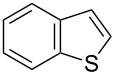 **9**	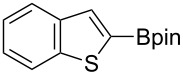 **9-B**	94 (>99)
7	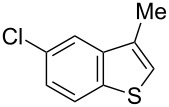 **10**	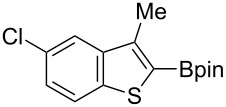 **10-B**	91 (99)
8	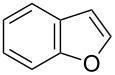 **11**	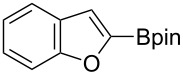 **11-B**	65 (87)
9	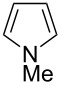 **12**	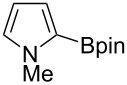 **12-B**	0 50 (52)^c^
10	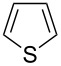 **13**	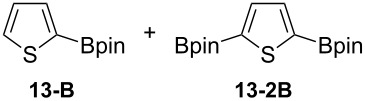	71 (91) **13-B**:**13-2B** = 1.1:1
11	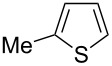 **14**	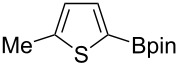 **14-B**	96 (>99)
12	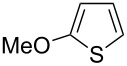 **15**	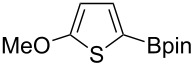 **15-B**	91 (>99)
13	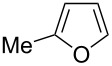 **16**	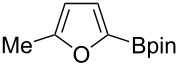 **16-B**	68 (92)

^a^Reaction conditions: heteroarene (0.50 mmol), **1g** (1.0 mmol), [Ir(OMe)(cod)]_2_ (0.050 mmol), ICy·HCl (0.10 mmol), NaO*t*-Bu (0.20 mmol) in methylcyclohexane (1.0 mL) at 110 °C for 4 h. After treatment with pinacol (2.0 mmol), the borylated product was converted to the corresponding pinacolate. In cases where NMR yield is modest, the recovered starting heteroarene can account for the material balance, unless otherwise noted. ^b^Debrominative borylation also occurred with a yield of 6%. ^c^Run using 1.0 mL of *N-*methylpyrrole instead of methylcyclohexane.

We next turned our attention to the borylation of benzene derivatives. Unfortunately, benzene derivatives proved to be much less reactive than heteroarenes when borylated with **1g**. For example, Ir/ICy-catalyzed borylation of benzene with **1g** afforded **17-B** in 48% isolated yield even when the reaction was conducted under neat conditions (Table 3, entry 1) (see the Supporting Information File 1 for details on the optimization for the borylation of benzene). Borylation was relatively independent of the electronic nature of the arene substrates, as indicated by the similar yields and regioselectivity observed with toluene, anisole and trifluoromethylbenzene (Table 3, entries 2–4). Similar to the reported C–H borylation using other boron sources, 1,3-disubstituted benzenes were borylated at the 5-position in a regioselective manner (Table 3, entry 5). Naphthalene also underwent borylation with **1g** at the less hindered 2-position (Table 3, entry 6).

**Table 3:** Scope of the arene substrates.^a^

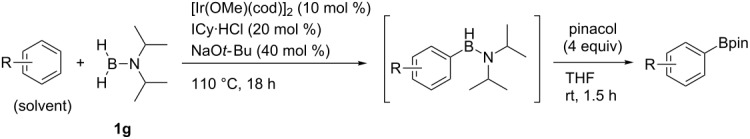				

Entry	Arene	Product	Isolated yield [%]	Ratio of *o/m/p* isomers

1	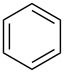 **17**	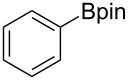 **17-B**	48	–
2	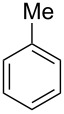 **18**	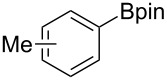 **18-B**	42	0/64/36
3	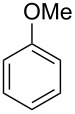 **19**	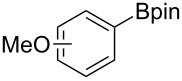 **19-B**	35	0/60/40
4	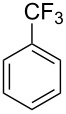 **20**	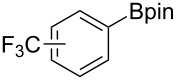 **20-B**	49	0/76/24
5	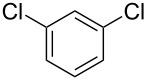 **21**	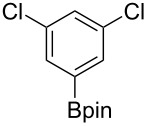 **21-B**	31	–
6^b^	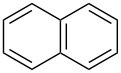 **22**	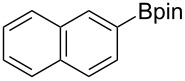 **22-B**	50	–

^a^Reaction conditions: arene (1.0 mL), **1g** (0.50 mmol), [Ir(OMe)(cod)]_2_ (0.050 mmol), ICy·HCl (0.10 mmol), NaO*t*-Bu (0.20 mmol) at 110 °C for 15 h. After treatment with pinacol (2.0 mmol), the borylated product was converted to the corresponding pinacolate. ^b^Naphthalene (3.0 mmol) was used in methylcyclohexane (1.0 mL).

**Scheme 2:**
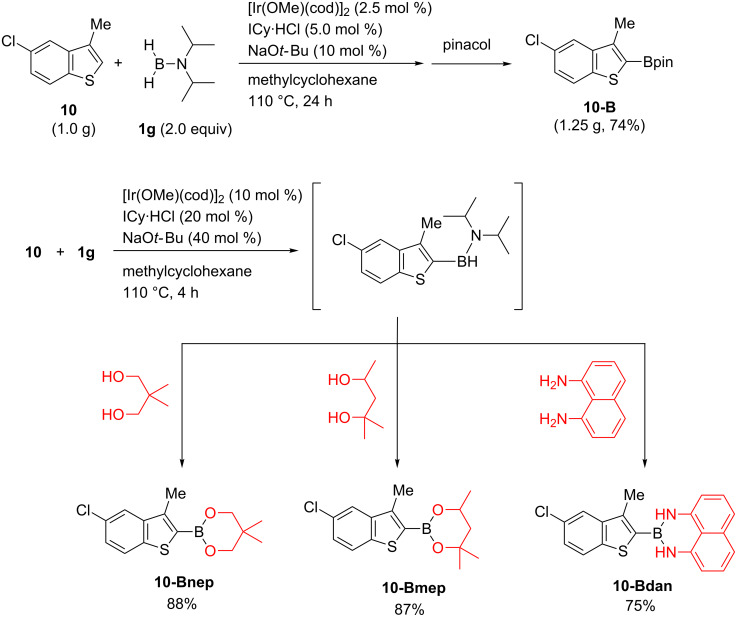
Scalability and derivatization.

Our protocol was performed on a gram scale without any difficulty using a lower loading of the iridium catalyst (Scheme 2, top). Using **1g** as the boron source in C–H borylation reactions has the synthetic advantage of allowing various substituents to be introduced onto the boron atom during the work-up stage simply by changing the reagents added. For example, addition of different diols delivered the corresponding boronic esters **10-Bnep** and **10-Bmep** (Scheme 2, bottom). It was also possible to introduce Suginome’s dan group, which allows us to use the borylated products in more elaborate manner, such as iterative cross-coupling reactions [20].

## Conclusion

We have developed a C–H borylation of arenes and heteroarenes using diisopropylaminoborane as a borylating reagent. An iridium(I) complex bearing an ICy ligand was the most efficient catalyst. The initially formed aminoborylated products can readily be converted to the corresponding organoboron compounds bearing various boron-protecting groups.

## Experimental

### Procedure for the Ir-catalyzed borylation of heteroarenes using **1g**

In a glovebox filled with nitrogen, [Ir(OMe)(cod)]_2_ (33.1 mg, 0.050 mmol, 0.10 equiv), ICy·HCl (26.2 mg, 0.10 mmol, 0.20 equiv), NaO*t*-Bu (19.2 mg, 0.20 mmol, 0.40 equiv) and methylcyclohexane (1.0 mL) were added to a 10 mL sample vial with a Teflon-sealed screwcap, and stirred for 5 min at room temperature. A heteroarene (0.50 mmol, 1.0 equiv) and **1g** (113.1 mg, 2.0 equiv) were added, and then the cap was screwed on seal the vial. The vial was stirred at 110 °C for 4 h. The reaction mixture was cooled to room temperature. Pinacol (236 mg, 2.0 mmol) in THF (2.0 mL) was added and the reaction mixture was stirred under N_2_ at room temperature for 1.5 h. The crude mixture was filtered through a pad of Celite and eluted with EtOAc. The filtrate was concentrated in vacuo and sampled for analysis by ^1^H NMR spectroscopy using 1,2-dichloroethane as an internal standard. The residue was purified by flash column chromatography over silica gel eluting with hexane/EtOAc. Product-containing fractions were concentrated in vacuo to give a pure borylated product.

## Supporting Information

File 1 Experimental procedures, data for optimization studies and copies of ^1^H and ^13^C NMR spectra.
