# Supplementary material for: Iridium/N-heterocyclic carbene-catalyzed C–H borylation of arenes by diisopropylaminoborane
Source: Beilstein J Org Chem. 2016 Apr 7;12:654–61. doi: 10.3762/bjoc.12.65 (PMC4901929; doi:10.3762/bjoc.12.65)
Supplement: File 1 — Experimental procedures, data for optimization studies and copies of 1H and 13C NMR spectra. [file Beilstein_J_Org_Chem-12-654-s001.pdf]

Supporting Information

for

Iridium/N-heterocyclic carbene-catalyzed C–H borylation of arenes by diisopropylaminoborane

Mamoru Tobisu*[1,2], Takuya Igarashi[2] and Naoto Chatani*[2]

Address: [1]Center for Atomic and Molecular Technologies, Graduate School of Engineering, Osaka University, Osaka 565-0871, Japan and [2]Department of Applied Chemistry, Faculty of Engineering, Osaka University, Osaka 565-0871, Japan

Email: Mamoru Tobisu - tobisu@chem.eng.osaka-u.ac.jp; Naoto Chatani - chatani@chem.eng.osaka-u.ac.jp

* Corresponding author

Experimental procedures, data for optimization studies and copies of ^{1}H and ^{13}C NMR spectra

CONTENTS

**I**	**General information**	**S2**
**II**	**Materials**	**S2**
**III**	**Synthesis of starting materials**	**S2**
**IV**	**Optimization studies**	**S3**
**V**	**Typical procedure**	**S9**
**VI**	**Spectroscopic data**	**S11**

I. General information

^{1}H NMR and ^{13}C NMR spectra were recorded on a JEOL ECS-400 spectrometer in $CDCl_3$ or C_6D_6 with tetrachloroethane as the internal standard. Data are reported as follows: chemical shift in ppm (δ), multiplicity (s = singlet, d = doublet, t = triplet, and m = multiplet), coupling constant (Hz), and integration. Infrared spectra (IR) were obtained using a JASCO FT/IR-4200 spectrometer; absorptions are reported in reciprocal centimeters with the following relative intensities: s (strong), m (medium), or w (weak). Mass spectra and high resolution mass spectra (HRMS) were obtained on a JEOL JMS-700 spectrometer. Analytical gas chromatography (GC) was carried out on a Shimazu GC-2014 gas chromatograph, equipped with a flame ionization detector. Melting points were determined using a Yamato melting point apparatus. Column chromatography was performed with SiO_2 (silicycle SilicaFlash F60 (230–400 mesh)).

II. Materials

$[Ir(OMe)(cod)]_2$ (TCI), ICy·HCl (TCI) and NaO*t*-Bu (TCI) were used as received. Methylcyclohexane was purified by distillation prior to use. *N*-methylindole (TCI), benzo[*b*]thiophene (TCI), 5-chloro3-methylbenzo[*b*]thiophene (TCI), 2,3-benzofuran (TCI), thiophene (TCI), 2-methylthiphene (TCI), 2-methoxythiophene (TCI), 2-methylfuran (TCI) and *N*-methylpyrrole (TCI) were obtained from commercial suppliers and used as received. All arenes (TCI) and naphthalene (Aldrich) were used as received. The other *N*-methylindoles used in this study were synthesized by the reaction of the corresponding indole with MeI according to the literature procedure.[1]

III. Synthesis of starring material

Diisopropylaminoborane (1g). [CAS: 22092-92-8]

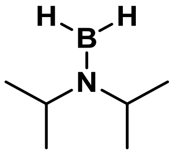

1g

Diisopropylaminoborane was prepared as described in literatures.[2]

To a stirred solution of diisopropylamine (28.2 mL, 200 mmol, 1.0 equiv) in THF (70 mL), H_2SO_4 (5.4 mL, 100 mmol, 0.5 equiv) were added at 0 °C. A white precipitate appeared immediately. After

1 Greulich, T. W.; Daniliuc, C. G.; Studer, A. *Org. Lett.* **2015**, *17*, 254.

2 a) Marciasini, L.; Richy, N.; Vaultier, M.; Pucheault, M. *Chem. Commun.* **2012**, *448*, 1553.

b) Marciasini, L.; Richy, N.; Vaultier, M.; Pucheault, M. *Adv. Synth. Catal.* **2013**, *6*, 1083.

the mixture was stirred at 0 °C for 30 min, $NaBH_4$ (8.2 g, 220 mmol, 1.1 equiv) was carefully added. The mixture was allowed to warm to room temperature and stirred for 4 h. The crude mixture was concentrated under vacuum and the residue was taken with toluene (100 mL), washed with water (4 × 100 mL). The organic phase was driedusing Na_2SO_4 and concentrated under reduced pressure to give an amine-borane complex as a colorless oil. The resulting amine-borane complex was then refluxed at 195 °C for 9 h, and the diisopropylaminoborane was distilled under N_2 to give 17.2 g (76% yield).

IV. Optimization studies

IV-I. Optimization studies for heteroarenes

The effect of the ligand was initially examined using **2** (0.50 mmol), **1g** (1.0 mmol), $[Ir(OMe)(cod)]_2$ (0.050 mmol), ligand (0.10 mmol) and base (0.20 mmol) in methylcyclohexane (1.0 mL) at 140 °C, 15 h (Table S1). Under these conditions, ICy·HCl was found to be an optimal ligand with a borylated product **2-B** being formed in 33% (Entry 17).

Table S1. Effect of ligands.

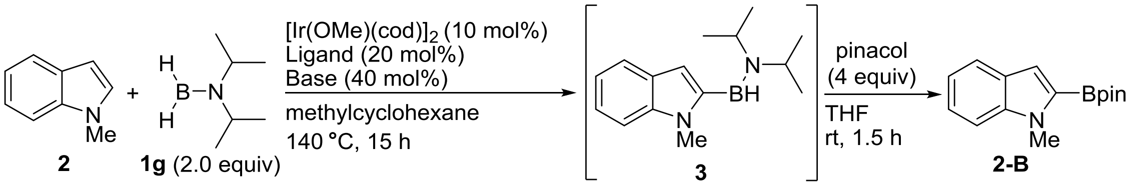

Entry	Ligand	Base	NMR yield [%]	2-Isomer/3-Isomer	Recovered **2** [%]
1	dtbpy	none	trace	-	85
2	dppe	none	2	100/0	48
3	dppf	none	11	91/9	65
4	Xantphos	none	18	56/44	59
5	DPEPhos	none	6	85/15	69
6	PPh_3	none	21	57/43	66
7	PCy_3	none	3	>99/1	71
8	$P(OPh)_3$	none	11	45/55	82
9	$P(C_6F_5)_3$	none	19	21/79	64
10	Cy-JohnPhos	none	11	64/36	72
11	JohnPhos	none	6	50/50	87
12	DavePhos	none	15	67/33	71
13	XPhos	none	21	71/29	61
14	SPhos	none	15	67/33	69
15	IPr•HCl	NaO^{t}Bu	3	>99/1	72
16	IMes•HCl	NaO^{t}Bu	5	>99/1	65
17	ICy•HCl	NaO^{t}Bu	33	88/12	53
18	I^{t}Bu•HCl	NaO^{t}Bu	0	-	89

The effect of the temperature was then examined using **2** (0.50 mmol), **1g** (1.0 mmol), $[Ir(OMe)(cod)]_2$ (0.050 mmol), ICy·HCl (0.10 mmol) and NaO*t*-Bu (0.20 mmol) in methylcyclohexane (1.0 mL) for 15 h (Table S2). Under these conditions, 110 °C was found to be an

optimal temperature with a borylated product **2-B** being formed in 58% (Entry 5).

Table S2. Effect of temperature.

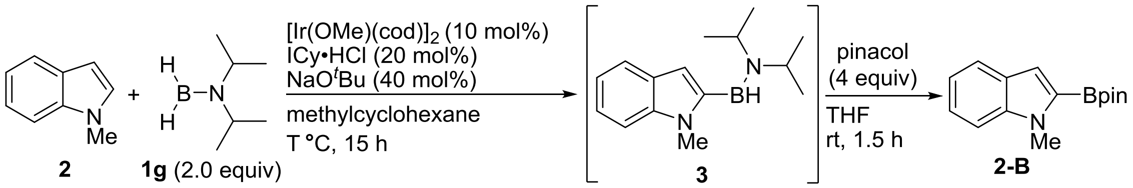

Entry	T [°C]	NMR yield [%]	2-Isomer/3-Isomer	Recovered **2** [%]
1	60	9	>99/1	90
2	80	21	>99/1	44
3	90	49	96/4	35
4	100	47	94/6	31
5	110	58	95/5	44
6	120	43	88/12	54
7	130	35	91/9	71
8	140	33	88/12	82

The effect of the amount of **1g** was then examined using **2** (0.50 mmol), **1g**, [Ir(OMe)(cod)]$_2$ (0.050 mmol), ICy·HCl (0.10 mmol) and NaO*t*-Bu (0.20 mmol) in methylcyclohexane (1.0 mL) at 110 °C, 15 h (Table S3). Under these conditions, 2 was found to be an optimal amount of **1g** with a borylated product **2-B** being formed in 58% (Entry 4).

Table S3. Effect of the amount of **1g**.

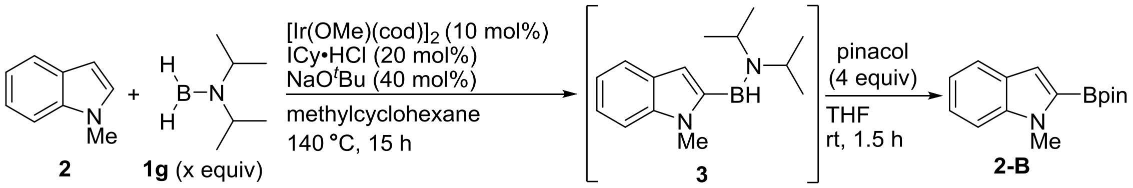

Entry	x [equiv]	NMR yield [%]	2-Isomer/3-Isomer	Recovered **2** [%]
1	1.0	22	91/9	54
2	1.25	41	93/7	34
3	1.5	41	93/7	33
4	2.0	58	95/5	44
5	2.5	46	96/4	42
6	3.0	46	96/4	36
7	3.5	41	98/2	30
8	4.0	59	93/7	49

The effect of the amount of ICy·HCl was then examined using **2** (0.50 mmol), **1g** (1.0 mmol), [Ir(OMe)(cod)]$_2$ (0.050 mmol), ICy·HCl and NaO*t*-Bu in methylcyclohexane (1.0 mL) at 110 °C, 15 h (Table S4). Under these conditions, 20 mol % was found to be an optimal amount of ICy·HCl with a borylated product **2-B** being formed in 58% (Entry 3).

Table S4. Effect of the amount of ICy•HCl.

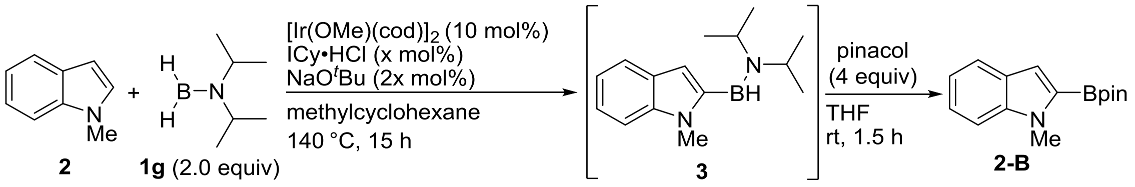

Entry	x [mol%]	NMR yield [%]	2-Isomer/3-Isomer	Recovered **2** [%]
1	10	24	83/17	59
2	15	31	94/6	54
3	20	58	95/5	44
4	25	61	95/5	33
5	30	56	93/7	46
6	35	61	92/8	42
7	40	57	91/9	51

The effect of the amount of $[Ir(OMe)(cod)]_2$ was then examined using **2** (0.50 mmol), **1g** (1.0 mmol), $[Ir(OMe)(cod)]_2$ (x mmol), ICy·HCl and NaO*t*-Bu in methylcyclohexane (1.0 mL) at 110 °C for 15 h (Table S5). Under these conditions, 10 mol % was found to be an optimal amount of $[Ir(OMe)(cod)]_2$ with a borylated product **2-B** being formed in 58% (Entry 4).

Table S5. Effect of the amount of $[Ir(OMe)(cod)]_2$.

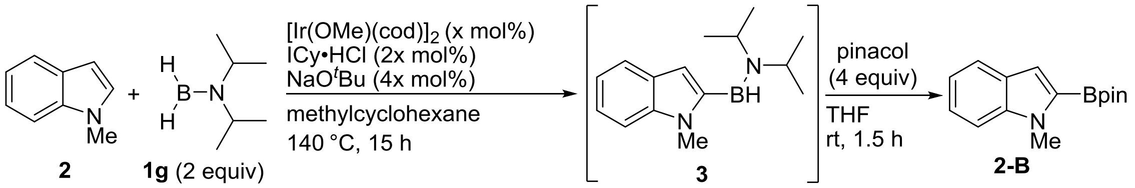

Entry	x [mol%]	NMR yield [%]	2-Isomer/3-Isomer	Recovered **2** [%]
1	3	42	90/10	71
2	5	41	98/2	65
3	7.5	45	96/4	47
4	10	58	95/5	44

The effect of the amount of $[Ir(OMe)(cod)]_2$ was also examined using **10** (0.50 mmol), **1g** (1.0 mmol), $[Ir(OMe)(cod)]_2$, ICy·HCl and NaO*t*-Bu in methylcyclohexane (1.0 mL) at 110 °C for 4 h (Table S6). Under these conditions, we were able to reduce the catalyst loading to 10 mol % without any loss of the yield of the product (Entry 4). This result indicate that benzo[*b*]thiophene is more reactive than *N*-methylindole toward this borylation.

Table S6. Effect of the amount of $[Ir(OMe)(cod)]_2$.

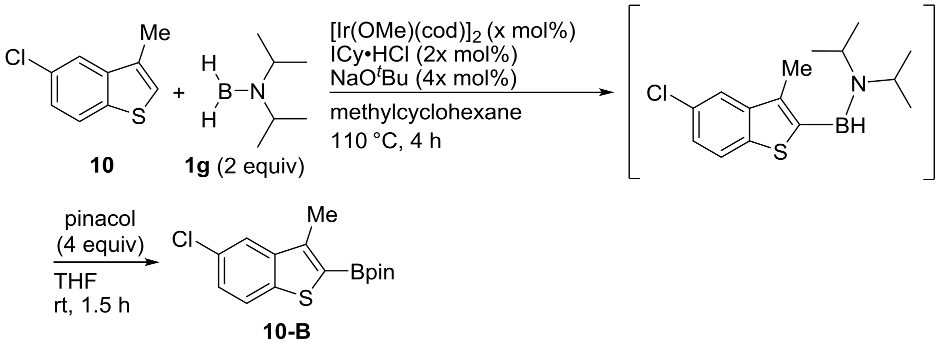

Entry	x [mol%]	NMR yield [%]	Recovered **10** [%]
1	1.25	76	30
2	2.5	66	34
3	5.0	>99	0
4	10	>99	0

The effect of the solvent was then examined using **2** (0.50 mmol), **1g** (1.0 mmol), $[Ir(OMe)(cod)]_2$ (0.050 mmol), ICy·HCl (0.10 mmol) and NaO*t*-Bu (0.20 mmol) in solvent (1.0 mL) at 110 °C, 15 h (Table S7). Under these conditions, methylcyclohexane was found to be an optimal solvent with a borylated product **2-B** being formed in 58% (Entry 1).

Table S7. Effect of solvents.

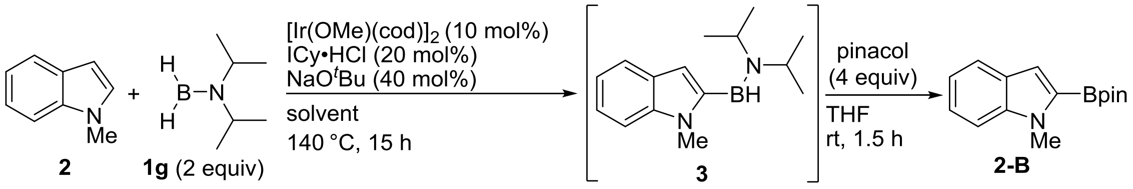

Entry	solvent	NMR yield [%]	2-Isomer/3-Isomer	Recovered **2** [%]
1	methylcyclohexane	58	95/5	44
2	hexane	45	91/9	42
3	dioxane	42	95/5	45
4	CH_3CN	0	-	86

The effect of H_2 scavengers was then examined using **2** (0.50 mmol), **1g** (1.0 mmol), $[Ir(OMe)(cod)]_2$ (0.050 mmol), ICy·HCl (0.10 mmol), NaO*t*-Bu (0.20 mmol) and H_2 scavenger (0.50 mmol) in methylcyclohexane (1.0 mL) at 110 °C, 15 h (Table S8). However, addition of a hydrogen scavenger did not improve the yield of **2-B** under these conditions (Entry 9).

Table S8. Effect of H_2 scavengers.

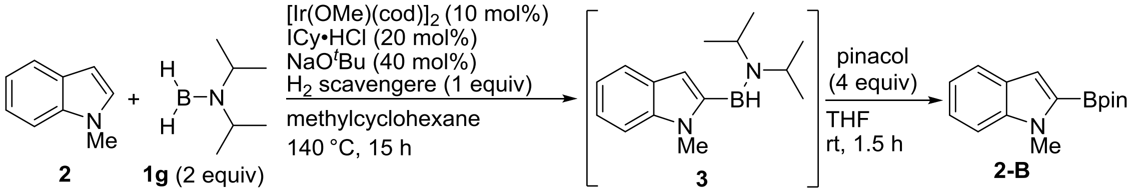

Entry	H_2 scavenger	NMR yield [%]	2-Isomer/3-Isomer	Recovered **2** [%]
1	cyclopentene	26	88/12	54
2	cyclohexene	19	84/16	34
3	1-methyl-1cyclohexene	43	91/9	33
4	cyclooctene	33	85/15	44
5	3,3-dimethyl-1-butene	66	89/11	42
6	3,3-dimethyl-1-butene	10	>99/1	36
7[a]	2,3,3-trimethyl-1-butene	21	90/10	30
8[b]	none	0	-	>99
9	none	58	95/5	44

a : H_2 scavenger was 2 equiv.

b : The reaction was conducted two neck flask in refluxing solvent.

The effect of the reaction time was then examined using **2** (0.50 mmol), **1g** (1.0 mmol), $[Ir(OMe)(cod)]_2$ (0.050 mmol), ICy·HCl (0.10 mmol) and NaO*t*-Bu (0.20 mmol) in methylcyclohexane (1.0 mL) at 110 °C (Table S9). Under these conditions, 4 h were found to be an optimal reaction time with a borylated product **2-B** being formed in 72% (Entry 3).

Table S9. Effect of reaction time.

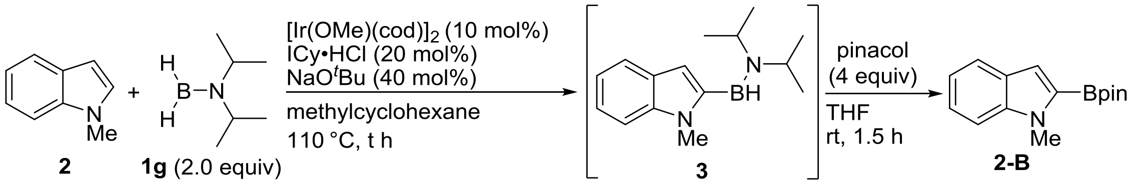

Entry	t [h]	NMR yield [%]	2-Isomer/3-Isomer	Recovered **2** [%]
1	1	37	>99/1	68
2	3	57	98/2	49
3	4	72	99/1	40
4	5	68	96/4	47
5	6	69	94/6	42
6	9	60	98/2	37
7	12	57	98/2	39
8	15	58	95/5	44
9	24	50	92/8	58
10	48	42	90/10	52

IV-II. Optimization studies for arenes

The effect of the ligand was initially examined using **2** (0.50 mmol), $[Ir(OMe)(cod)]_2$ (0.050 mmol), ligand (0.10 mmol) and NaO*t*-Bu (0.20 mmol) in benzene (1.0 mL) at 110 °C, 18 h (Table S10). Under these conditions, ICy·HCl was found to be an optimal ligand with a borylated product **17-B** being formed in 53% (Entry 1).

Table S10. Effect of ligands.

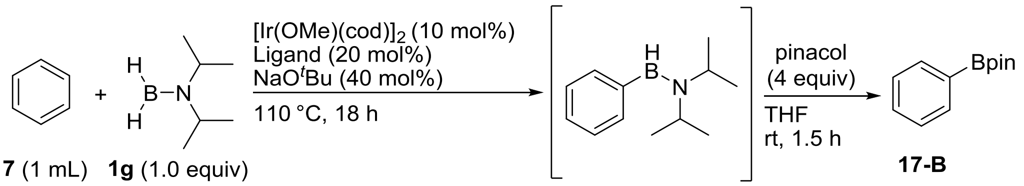

17 (1 mL) **1g** (1.0 equiv) **17-B**

Entry	Ligand	GC yield [%]
1	ICy•HCl	53
2	IPrHCl	2
3	IMes•HCl	0
4	BICy•HCl	0
5	I(1-Ad)•HCl	1

The effect of the temperature was then examined using **2** (0.50 mmol), $[Ir(OMe)(cod)]_2$ (0.050 mmol), ICy·HCl (0.10 mmol) and NaO*t*-Bu (0.20 mmol) in benzene (1.0 mL) for 15 h (Table S11). Under these conditions, 110 °C was found to be an optimal temperature with a borylated product **17-B** being formed in 47% (Entry 4).

Table S11. Effect of temperature.

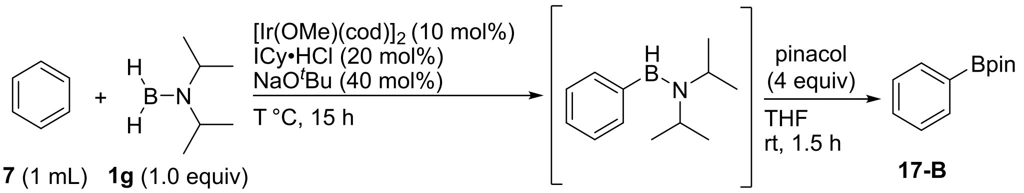

17 (1 mL) **1g** (1.0 equiv) **17-B**

Entry	T [°C]	GC yield [%]
1	60	5
2	80	30
3	100	26
4	110	47
5	115	31
6	120	13

The effect of the reaction time was then examined using **2** (0.50 mmol), $[Ir(OMe)(cod)]_2$ (0.050 mmol), ICy·HCl (0.10 mmol) and NaO*t*-Bu (0.20 mmol) in benzene (1.0 mL) at 110 °C (Table S12). Under these conditions, 18 h was found to be an optimal reaction time with a borylated product **17-B** being formed in 53% (Entry 7).

Table S12.Effect of reaction time.

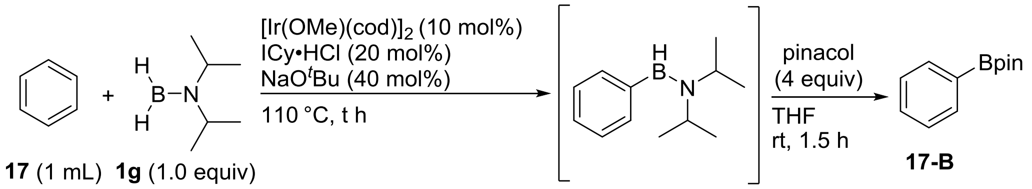

Entry	t [h]	GC yield [%]
1	2	19
2	4	28
3	6	40
4	8	37
5	12	42
6	15	47
7	18	53
8	24	40
9	48	32

The effect of the amount of ICy·HCl was initially examined using **2** (0.50 mmol), $[Ir(OMe)(cod)]_2$ (0.050 mmol), ICy·HCl and NaO*t*-Bu in benzene (1.0 mL) at 110 °C for 18 h (Table S13). Under these conditions, 20 mol % was found to be an optimal amount of ICy·HCl with a borylated product **17-B** being formed in 53% (Entry 1).

Table S13. Effect of the amount of ICy•HCl.

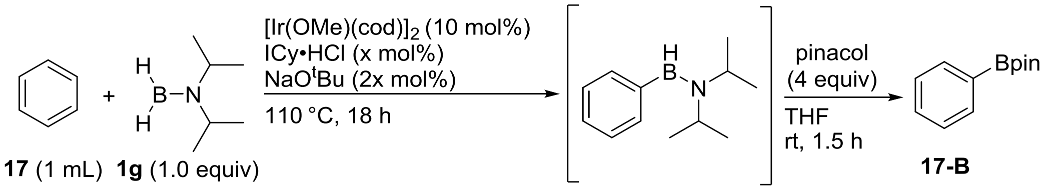

Entry	x [mol%]	GC yield [%]
1	20	53
2	25	27
3	30	31
4	40	19

IV. Typical procedure

Method A: Procedure for the Ir-catalyzed borylation of heteroarenes using 1g.

In a glovebox filled with nitrogen, $[Ir(OMe)(cod)]_2$ (33.1 mg, 0.050 mmol, 0.10 equiv), ICy·HCl (26.2 mg, 0.10 mmol, 0.20 equiv), NaO*t*-Bu (19.2 mg, 0.20 mmol, 0.40 equiv) and methylcyclohexane (1.0 mL) were added to a 10 mL-sample vial with a Teflon-sealed screwcap, and stirred for 5 min at room temperature. A heteroarene (0.50 mmol, 1.0 equiv) and **1g** (113.1 mg, 2.0 equiv) were added, and then the cap was screwed on seal the vial. The vial was stirred at 110 °C for 4 h. The reaction mixture was cooled to room temperature. Pinacol (236 mg, 2.0 mmol) in THF (2.0

mL) was added and the reaction mixture was stirred under N_2 at room temperature for 1.5 h. The crude mixture was filtered through a pad of Celite and eluted with EtOAc. The filtrate was concentrated in vacuo and sampled for analysis by ^{1}HNMR spectroscopy using 1,2-dichloroethane as an internal standard. The residue was purified by flash column chromatography over silica gel eluting with hexane/EtOAc. Product-containing fractions were concentrated *in vacuo* to give a pure borylated product.

Method B: Procedure for the Ir-catalyzed borylation of arenes using 1g.

In a glovebox, $[Ir(OMe)(cod)]_2$ (33.1 mg, 0.050 mmol, 0.10 equiv), ICy•HCl (26.2 mg, 0.10 mmol, 0.20 equiv), NaO*t*-Bu (19.2 mg, 0.20 mmol, 0.40 equiv) and benzene (1.0 mL) were added to a 10 mL-sample vial with a Teflon-sealed screwcap, and stirred for 5 min at room temperature. **1g** (113.1 mg, 1.0 mmol, 2.0 equiv) was added, and then the cap was screwed on to seal the vial. The vial was stirred at 110 °C for 18 h. The reaction mixture was cooled to room temperature. Pinacol (236 mg, 2.0 mmol, 4.0 equiv) in THF (2.0 mL) was added and the reaction mixture was stirred for 1.5 h at room temperature under N_2. The crude mixture was filtered through a pad of Celite and eluted with EtOAc. The filtrate was concentrated in vacuo and sampled for analysis by ^{1}H NMR spectroscopy using 1,2-dichloroethane as an internal standard. The residue was purified by flash column chromatography over silica gel eluting with hexane/EtOAc. Product-containing fractions were concentrated in vacuo to give a pure borylated product.

A procedure for the gram scale synthesis of 2-borylated 10.

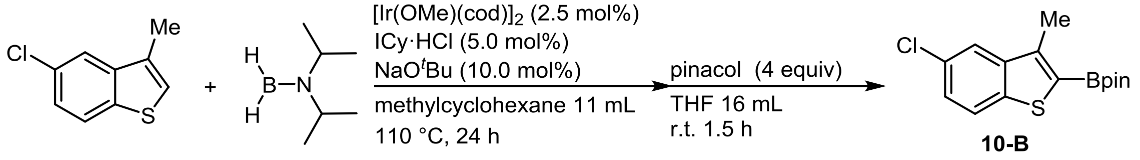

In a glovebox, $[Ir(OMe)(cod)]_2$ (91.0 mg, 0.138 mmol, 0.025 equiv), ICy·HCl (73.8 mg, 0.275 mmol, 0.050 equiv), NaO*t*-Bu (52.8 mg, 0.55 mmol, 0.10 equiv) and methylcyclohexane (11.0 mL) were added to a 190 mL-sample vial with a Teflon-sealed screwcap, and stirred for 5 min at room temperature. Compounds **10** (1.00 g, 5.50 mmol, 1.0 equiv) and **1g** (1.24g, 11.0 mmol, 2.0 equiv) were added, and then the cap was screwed on to seal the vial. The vial was stirred at 110 °C for 24 h. The reaction mixture was cooled to room temperature. Pinacol (2.57 g, 22.0 mmol, 4.0 equiv) in THF (16 mL) was added and the reaction mixture was stirred under N_2 at room temperature for 1.5 h. The crude mixture was filtered through a pad of Celite and eluted with EtOAc. The filtrate was concentrated in vacuo and sampled for analysis ^{1}H NMR spectroscopy using 1,2-dichloroethane as an internal standard. The residue was purified by flash column chromatography over silica gel eluting with hexane/EtOAc (40/1) solution. Product-containing fractions were concentrated in vacuo to give **10-B** as a white solid (1.25 g, 74%).

V. Spectroscopic Data

1-Methyl-2-(4,4,5,5-tetramethyl-1,3,2-dioxaborolan-2-yl)-1*H*-indole (2-B). [CAS: 596819-10-2]

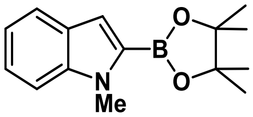

Method A was used. R_f 0.14 (Hexane/EtOAc =20/1). White solid (83 mg, 65%).

^{1}H NMR (C_6D_6, 399.78 MHz): δ 1.12 (s, 12H), 3.69 (s, 3H), 7.13 (d, *J* = 7.8 Hz, 2H), 7.27 (td, *J* = 0.9, 7.8 Hz, 1H), 7.57 (s, 1H), 7.68-7.70 (m, 1H).

^{1}H NMR ($CDCl_3$, 399.78 MHz): δ1.37 (s, 12H), 3.98 (s, 3H), 7.07-7.10 (m, 1H), 7.14 (s, 1H), 7.24-7.28 (m, 1H), 7.35 (d, *J* = 8.2 Hz, 1H), 7.64 (d, *J* = 8.2 Hz, 1H).

^{13}C NMR (C_6D_6, 100.53 MHz): δ 24.9, 32.1, 83.6, 110.1, 115.6, 119.9, 122.2, 123.6, 128.8, 140.9.

HRMS (EI): Calcd for $C_{15}H_{20}BNO_2$ 257.1587, Found 257.1585.

^{1}H NMR spectroscopic data was in agreement with the reported value.[3]

5-Methoxy-1-methyl-2-(4,4,5,5-tetramethyl-1,3,2-dioxaborolan-2-yl)-1*H*-indole (4-B).
[CAS: 1256360-41-4]

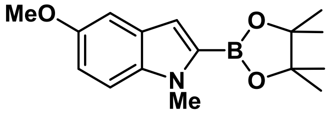

Method A was used. R_f 0.057 (Hexane/EtOAc = 40/1). White solid (69 mg, 48%).

^{1}H NMR (C_6D_6, 399.78 MHz): δ 1.13 (s, 12H), 3.45 (s, 3H), 3.68 (s, 3H), 6.99 (d, *J* = 9.2 Hz, 1H), 7.08 (d, *J* = 2.6 Hz, 1H), 7.19 (d, *J* = 2.6 Hz, 1H), 7.56 (s, 1H).

^{13}C NMR (C_6D_6, 100.53 MHz): δ 24.9, 30.1, 32.2, 55.2, 83.6, 102.4, 110.9, 114.9, 115.4, 136.53, 154.9.

HRMS (EI): Calcd for $C_{16}H_{22}BNO_3$ 287.1693, Found 257.1695.

^{1}H NMR spectroscopic data was in agreement with the reported value.[3]

5-Fluoro-1-methyl-2-(4,4,5,5-tetramethyl-1,3,2-dioxaborolan-2-yl)-1*H*-indole (5-B).
[CAS: 1683582-67-3]

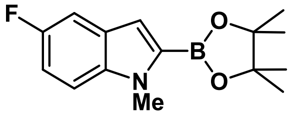

Method A was used. R_f 0.085 (Hexane/EtOAc = 40/1). White solid (103 mg, 75%).

^{1}H NMR (C_6D_6, 399.78 MHz): δ 1.10 (s, 12H), 3.57 (s, 3H), 6.79 (dd, *J* = 4.1, 9.2 Hz, 1H), 7.00 (dt, *J* = 2.3, 9.2 Hz, 1H), 7.28 (dd, *J* = 2.3, 9.6 Hz, 1H), 7.36 (s, 1H).

---

3 Furukawa, T.; Tobisu, M.; Chatani, N. *Chem. Commun.* **2015**, *51*, 6508.

^{13}C NMR (C_6D_6, 100.53 MHz): δ 24.8, 32.2, 83.7, 106.3 (d, *J* = 23 Hz), 110.8 (d, *J* = 9.5 Hz), 112.2 (d, *J* = 27 Hz), 115.1 (d, *J* = 4.8 Hz), 128.7 (d, *J* = 9.5 Hz), 137.5, 158.5 (d, *J* = 234 Hz).
HRMS (EI): Calcd for $C_{15}H_{20}BFNO_2$ 276.1568, Found 276.1570.
^{1}H NMR spectroscopic data was in agreement with the reported value.[3]

5-Chloro-1-methyl-2-(4,4,5,5-tetramethyl-1,3,2-dioxaborolan-2-yl)-1*H*-indole (6-B).

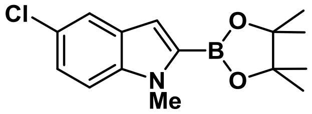

Method A was used. After purification by flush column chromatography over silica gel, a mixture of borylated product **6B** and **6** were obtained (**6-B**: 66%, **6**: 22%). GC/MS analysis revealed the existence of **6-B** and **6**; **6-B** had an m/z of 291 (M^+), and **6** had an m/z of 165 (M^+). The identity and ratio of **6** and **6B** was determined by the ^{1}HNMR spectrum of the mixture. The resonances specific to each compound are as follows: ^{1}H NMR (C_6D_6, 399.78 MHz): δ 0.454 (s, 3H, **6**), 3.52 (s, 3H, **6-B**).
R_f 0.086 (Hexane/EtOAc = 40/1). White solid as a 3:1 mixture of **6-B** and **6** (114 mg). Mp = 111 °C.
^{1}H NMR (C_6D_6, 399.78 MHz): δ 1.10 (s, 12H), 3.52 (s, 3H), 6.76 (d, 1H, *J* = 8.8 Hz), 7.23 (dd, *J* = 2.0, 8.7 Hz, 1H), 7.33 (s, 1H), 7.60 (d, *J* = 1.9 Hz, 1H).
^{13}C NMR (C_6D_6, 100.53 MHz): δ 24.8, 32.1, 83.8, 111.1, 114.8, 121.4, 123.9, 125.7, 129.5, 139.0.
IR (ATR): 2977 w, 2927 w, 2361 m, 2339 w, 1735 w, 1649 w, 1558 w, 1526 m, 1438 w, 1361 s, 1306 s, 1264 m, 1208 w, 1137 s, 1106 m, 1077 m, 1030 m, 974 w, 949 w, 866 m, 849 s, 805 m, 780 w, 732 w, 692 w, 671m.
MS m/z (% relative intensity): 293 (32), 292 (24), 291 (M^+, 100), 290 (25), 218 (12), 209 (18), 208 (17), 207 (10), 206 (31), 205 (12), 193 (12), 192 (21), 191 (35), 190 (22).
HRMS (EI): Calcd for $C_{15}H_{19}BClNO_2$ 291.1197, Found 291.1204.

5-Bromo-1-methyl-2-(4,4,5,5-tetramethyl-1,3,2-dioxaborolan-2-yl)-1*H*-indole (7-B) [CAS: 1192037-87-8] **and 1-methyl-5-(4,4,5,5-tetramethyl-1,3,2-dioxaborolan-2-yl)-1*H*-indole (7-B').** [CAS: 837392-62-8]

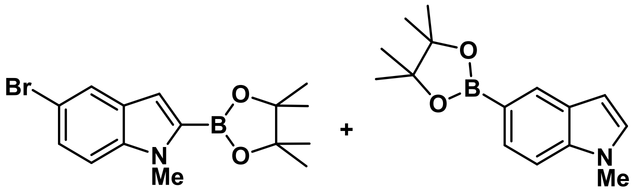

7-B (50%) **7-B' (6%)**

Method A was used. After purification by flush column chromatography over silica gel, a mixture of two borylated products and **7** was obtained (**7-B**: 50%, 7-**B'**: 6%, **7**: 20%). GC/MS analysis revealed the existence of two borylated products and **7**; **7-B** had an m/z of 335 (M^+), **7-B'** had an m/z of 257

(M^+), and **7** had an m/z of 209 (M^+). The identity and ratio of each of these was determined by the ^{1}HNMR spectrum of the mixture. The resonances specific to each isomer are as follows: ^{1}H NMR (C_6D_6, 399.78 MHz): δ 2.74 (s, 3H, **7**), 3.50 (s, 3H, **7-B**), 3.69 (s, 3H, **7-B'**).
MS m/z (% relative intensity) **7-B**: 338 (16), 337 (99), 336 (39), 335 (M^+, 100), 334 (23), 255 (15), 253 (15), 252 (25), 251 (11), 250 (22), 237 (24), 236 (24), 235 (27), 234 (14), 183 (11), 156 (10).
7-B': 258 (18), 257 (M^+, 100), 256 (25), 184 (21), 175 (21), 172 (31), 158 (15), 157 (36), 156 (25).
HRMS (EI) **7-B**: Calcd for $C_{15}H_{19}BBrNO_2$ 335.0692, Found 335.0689.
7-B': Calcd for $C_{15}H_{20}BNO_2$ 257.1587, Found 257.1583.
^{1}H NMR spectroscopic data was in agreement with the reported value.[4]

1,4-Dimethyl-2-(4,4,5,5-tetramethyl-1,3,2-dioxaborolan-2-yl)-1*H*-indole (8-B).

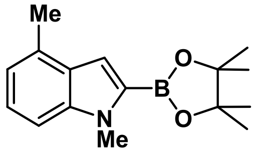

Method A was used. R_f 0.14 (Hexane/EtOAc = 40/1). White solid (69 mg, 51%). Mp = 151 °C.
^{1}H NMR (C_6D_6, 399.78 MHz): δ 1.14 (s, 12H), 2.51 (s, 3H), 3.71 (s, 3H), 6.97 (d, *J* = 7.4 Hz, 1H), 7.04 (d, *J* = 8.2 Hz, 1H), 7.25 (t, *J* = 8.2 Hz, 1H), 7.64 (s, 1H).
^{13}C NMR (C_6D_6, 100.53 MHz): δ 18.8, 24.9, 32.3, 83.6, 107.9, 114.3, 120.1, 124.0, 128.8, 131.4, 140.8.
IR (ATR): 2975 w, 2921 w, 2361 w, 1606 w, 1580 w, 1522 m, 1496 w, 1467 w, 1383 m, 1349 w, 1317 m, 1293 m, 1258 m, 1239 m, 1216 w, 1139 m, 1111 w, 1070 m, 964 w, 858 m, 827 w, 805 w, 770m , 739 m, 688 m, 670 w.
MS m/z (% relative intensity): 272 (18), 271 (M^+, 100), 270 (25), 198 (16), 189 (29), 188 (11), 172 (10), 171 (29), 170 (24).
HRMS (EI): Calcd for $C_{16}H_{22}BNO_2$ 271.1744, Found 271.17430.

2-(Benzo[*b*]thiophen-2-yl)-4,4,5,5-tetramethyl-1,3,2-dioxaborolane (9-B). [CAS: 376584-76-8]

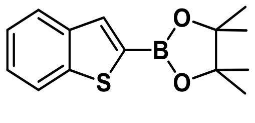

Method A was used. R_f 0.086 (Hexane/EtOAc = 40/1). White solid (122 mg, 94%).
^{1}H NMR (C_6D_6, 399.78 MHz): δ 1.10 (s, 12H), 7.01-7.09 (m, 2H), 7.54-7.58 (m, 2H), 8.06 (s, 1H).
^{1}H NMR ($CDCl_3$, 399.78 MHz): δ 1.38 (s, 12H), 7.35-7.39 (m, 2H), 7.85-7.92 (m, 3H).
^{13}C NMR (C_6D_6, 100.53 MHz): δ 24.8, 84.3, 124.4, 124.7, 122.9, 125.6, 135.3, 141.0, 144.4.
HRMS (EI) **A**: Calcd for $C_{14}H_{17}BO_2S$ 260.1042, Found 260.1040.

---

[4] Stadlwieser, J. F.; Dambaur, M. E. *Helv. Chim. Acta.* **2006**, *89*, 936.

^{1}H NMR spectroscopic data was in agreement with the reported value.[5]

2-(5-Chloro-3-methylbenzo[*b*]thiophen-2-yl)-4,4,5,5-tetramethyl-1,3,2-dioxaborolane (10-B).
[CAS: 1809298-96-1]

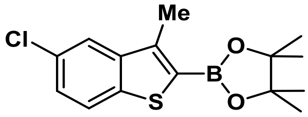

Method A was used. R_f 0.22 (Hexane/EtOAc = 40/1). White solid (140 mg, 91%).
^{1}H NMR (C_6D_6, 399.78 MHz): δ 1.07 (s, 12H), 2.51 (s, 3H), 7.04 (dd, *J* = 1.8, 8.7 Hz, 1H), 7.18 (d, *J* = 8.7 Hz, 1H), 7.63 (d, *J* = 1.8 Hz, 1H).
^{13}C NMR (C_6D_6, 100.53 MHz): δ 14.0, 24.8, 84.1, 122.7, 124.0, 126.1, 130.5, 141.9, 142.9, 143.4.
HRMS (EI): Calcd for $C_{15}H_{18}BClO_2S$ 308.0809, Found 308.0811.
^{1}H NMR spectroscopic data was in agreement with the reported value.[5]

2-(Benzofuran-2-yl)-4,4,5,5-tetramethyl-1,3,2-dioxaborolane (11-B). [CAS: 402503-13-3]

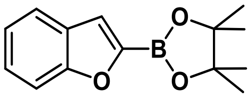

Method A was used. R_f 0.057 (Hexane/EtOAc = 40/1). White solid (79 mg, 65%).
^{1}H NMR (C_6D_6, 399.78 MHz): δ 1.08 (s, 12H), 6.98-7.08 (m, 2H), 7.34-7.36 (m, 1H), 7.40-7.42 (m, 1H), 7.48 (d, *J* = 0.92 Hz, 1H).
^{1}H NMR ($CDCl_3$, 399.78 MHz): δ 1.39 (s, 12H), 7.23 (t, *J* = 7.8 Hz, 1H), 7.34 (td, 0.9, *J* = 8.2 Hz, 1H), 7.40 (s, 1H), 7.57 (d, *J* = 8.2 Hz, 1H), 7.63 (1H, *J* = 7.8 Hz, 1H).
^{13}C NMR (C_6D_6, 100.53 MHz): δ 24.8, 84.4, 112.1, 120.1, 122.2, 123.0, 126.3, 158.3. One carbon peak is overlapped with solvent peaks.
HRMS (EI): Calcd for $C_{14}H_{17}BO_3$ 244.1271, Found 244.1276.
^{1}H NMR spectroscopic data was in agreement with the reported value.[5]

1-Methyl-2-(4,4,5,5-tetramethyl-1,3,2-dioxaborolan-2-yl)-1*H*-pyrrole (12-B).
[CAS: 850567-47-4]

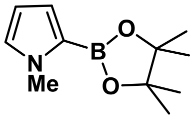

Method B was used except that the reaction was conducted in *N*-methyl pyrrole (1.0 mL).
R_f 0.14 (Hexane/EtOAc = 40/1). White solid (52 mg, 50%).
^{1}H NMR (C_6D_6, 399.78 MHz): δ 1.11 (s, 12H), 3.52 (s, 3H), 6.30 (dd, *J* = 1.4, 2.3 Hz, 1H), 7.22 (t, *J*

---

[5] Furukawa, T.; Tobisu, M.; Chatani, N. *J. Am. Chem. Soc.* **2015**, *137*, 12211.

= 1.8 Hz, 1H), 7.33 (dd, *J* = 1.4, 2.3 Hz, 1H).
^{13}C NMR (C_6D_6, 100.53 MHz): δ 24.9, 36.3, 83.0, 109.2, 123.4. One carbon peak is overlapped with solvent peaks.
HRMS (EI): Calcd for $C_{11}H_{18}BNO_2$ 207.1431, Found 207.1431.
^{1}H NMR spectroscopic data was in agreement with the reported value.[3]

4,4,5,5-Tetramethyl-2-(thiophen-2-yl)-1,3,2-dioxaborolane (13-B) and 2,5-bis(4,4,5,5-tetramethyl-1,3,2-dioxaborolan-2-yl)thiophene (13-2B).
Method A was used. The product was obtained as a mixture of mono and diborylated thiophenes. It was possible to purify two products by flush column chromatography over silica gel.

4,4,5,5-Tetramethyl-2-(thiophen-2-yl)-1,3,2-dioxaborolane (13-B). [CAS: 193978-23-3]

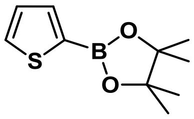

R_f 0.22 (Hexane/EtOAc = 40/1). White solid (41 mg, 39%).
^{1}H NMR (C_6D_6, 399.78 MHz): δ 1.08 (s, 12H), 6.89 (m, 1H), 7.18 (dd, *J* = 0.92, 4.6 Hz, 1H), 7.88-7.89 (m, 1H).
^{13}C NMR (C_6D_6, 100.53 MHz): δ 24.8, 84.0, 128.5, 132.8, 137.7.
HRMS (EI): Calcd for $C_{10}H_{15}BO_2S$ 210.0886, Found 210.0889.
^{1}H NMR spectroscopic data was in agreement with the reported value.[6]

2,5-Bis(4,4,5,5-tetramethyl-1,3,2-dioxaborolan-2-yl)thiophene (13-2B). [CAS: 175361-81-6]

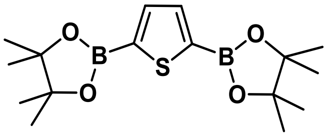

R_f 0.14 (Hexane/EtOAc = 40/1). White solid (54 mg, 32%).
^{1}H NMR (C_6D_6, 399.78 MHz): δ 1.03 (s, 24H), 7.97 (s. 2H).
^{1}H NMR ($CDCl_3$, 399.78 MHz): δ 1.34 (s, 24H), 7.66 (s, 2H).
^{13}C NMR (C_6D_6, 100.53 MHz): δ 24.8, 84.1, 138.6.
HRMS (EI): Calcd for $C_{16}H_{26}B_2O_4S$ 336.1738, Found 336.1738.
^{1}H NMR spectroscopic data was in agreement with the reported value.[7]

6 Boller, T. M.; Murphy, J. M.; Hapke, M.; Ishiyama, T.; Miyaura, N.; Hartwig, J. F. *J. Am. Chem. Soc.* **2005**, *127*, 14263.
7 Guerrand, H. D. S.; Marciasini, L. D.; Jousseaume, M.; Vaultier. M.; Pucheault, M. *Chem. Eur. J.* **2014**, *20*, 5573.

4,4,5,5-Tetramethyl-2-(5-methylthiophen-2-yl)-1,3,2-dioxaborolane (14-B). [CAS: 476004-80-5]

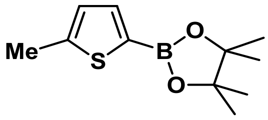

Method A was used. R_f 0.14 (Hexane/EtOAc = 40/1). Colorless oil (108 mg, 96%).

^{1}H NMR (C_6D_6, 399.78 MHz): δ 1.09 (s, 12H), 2.11 (s, 3H), 6.62 (d, *J* = 3.3 Hz, 1H), 7.8 (d, *J* = 3.5 Hz, 1H).

^{13}C NMR (C_6D_6, 100.53 MHz): δ 15.1, 24.9, 83.9, 127.5, 138.4, 147.8.

HRMS (EI): Calcd for $C_{11}H_{17}BO_3S$ 208.1271, Found 208.1272.

^{1}H NMR spectroscopic data was in agreement with the reported value.[5]

2-(5-Methoxythiophen-2-yl)-4,4,5,5-tetramethyl-1,3,2-dioxaborolane (15-B).
[CAS: 596819-12-4]

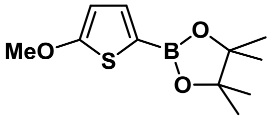

Method A was used. R_f 0.14 (Hexane/EtOAc = 40/1). Colorless oil (109 mg, 91%).

^{1}H NMR (C_6D_6, 399.78 MHz): δ 1.09 (s, 12H), 3.24 (s, 3H), 6.07 (d, *J* = 4.0 Hz, 1H), 7.62 (d, *J* = 3.9 Hz, 1H).

^{1}H NMR ($CDCl_3$, 399.78 MHz): δ 1.32 (s, 12H), 3.92 (s, 3H), 6.30 (d, *J* = 3.8 Hz, 1H), 7.33 (d, *J* = 3.8 Hz, 1H).

^{13}C NMR (C_6D_6, 100.53 MHz): δ 24.9, 59.7, 83.8, 106.4, 137.2, 173.5.

HRMS (EI): Calcd for $C_{16}H_{26}B_2O_4S$ 240.0991, Found 240.0994.

^{1}H NMR spectroscopic data was in agreement with the reported value.[5]

4,4,5,5-Tetramethyl-2-(5-methylfuran-2-yl)-1,3,2-dioxaborolane (16-B). [CAS: 338998-93-9]

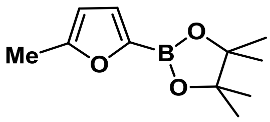

Method A was used. R_f 0.028 (Hexane/EtOAc = 40/1). Colorless oil (71 mg, 68%).

^{1}H NMR (C_6D_6, 399.78 MHz): δ 1.09 (s, 12H), 1.99 (s, 3H), 5.81 (d, *J* = 2.3 Hz, 1H), 7.22 (d, *J* = 3.2 Hz, 1H).

^{13}C NMR (C_6D_6, 100.53 MHz): δ 13.6, 24.8, 83.7, 107.2, 125.4, 157.6.

HRMS (EI): Calcd for $C_{11}H_{17}BO_3$ 208.1271, Found 208.1270.

^{1}H NMR spectroscopic data was in agreement with the reported value.[8]

---

8 Hatanaka, T.; Ohki, Y.; Tatsumi, K. *Chem. Asian. J.* **2010**, *5*, 1657.

4,4,5,5-Tetramethyl-2-phenyl-1,3,2-dioxaborolane (17-B). [CAS: 24388-23-6]

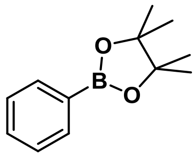

Method B was used. R_f 0.20 (Hexane/EtOAc = 40/1). White solid (49 mg, 48%).

^{1}H NMR (C_6D_6, 399.78 MHz): δ 1.11 (s, 12H), 7.21-7.22 (m, 3H), 8.15-8.17 (m, 2H).

^{1}H NMR ($CDCl_3$, 399.78 MHz): δ 1.35 (s, 12H), 7.34-7.38 (m, 2H), 7.44-7.48 (m, 1H), 7.78-7.82 (m, 1H).

^{13}C NMR ($CDCl_3$, 100.53 MHz): δ 25.0, 83.9, 127.8, 131.4, 134.9.

HRMS (EI): Calcd for $C_{12}H_{17}BO_2$ 204.1322, Found 204.1321.

Borylation of toluene (Entry 2, Table 3). [CAS: 253342-48-2] and [CAS: 195062-57-8]

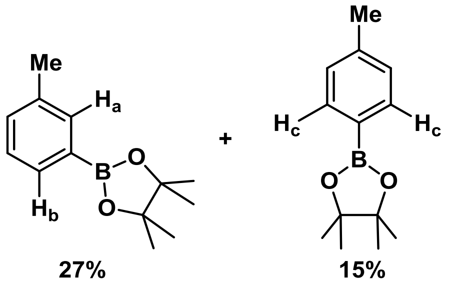

Method B was followed except that the reaction was conducted in toluene (1.0 mL). After purification by flash column chromatography over silica gel eluting with hexane/AcOEt = 20/1, a mixture of two isomers was obtained. GC/MS analysis revealed the two isomers of the borylated products had an m/z of 218 (M^+). The identity and ratio of each of the two isomers wre determined by comparing the ^{1}H NMR spectrum of the product mixture with those reported in the literature.[9] The resonances specific to each isomer are as follows: ^{1}H NMR ($CDCl_3$, 399.78 MHz): 7.60-7.64 ppm (m, 2H, meta isomer, H_a and H_b), 7.70 ppm (d, J = 7.8 Hz, 2H, para isomer, H_c).

[9] Ishiyama, T.; Takagi, J.; Ishida, K.; Miyaura, N.; Hartwig, J. F. *J. Am. Chem. Soc.* **2002**, *124*, 390.

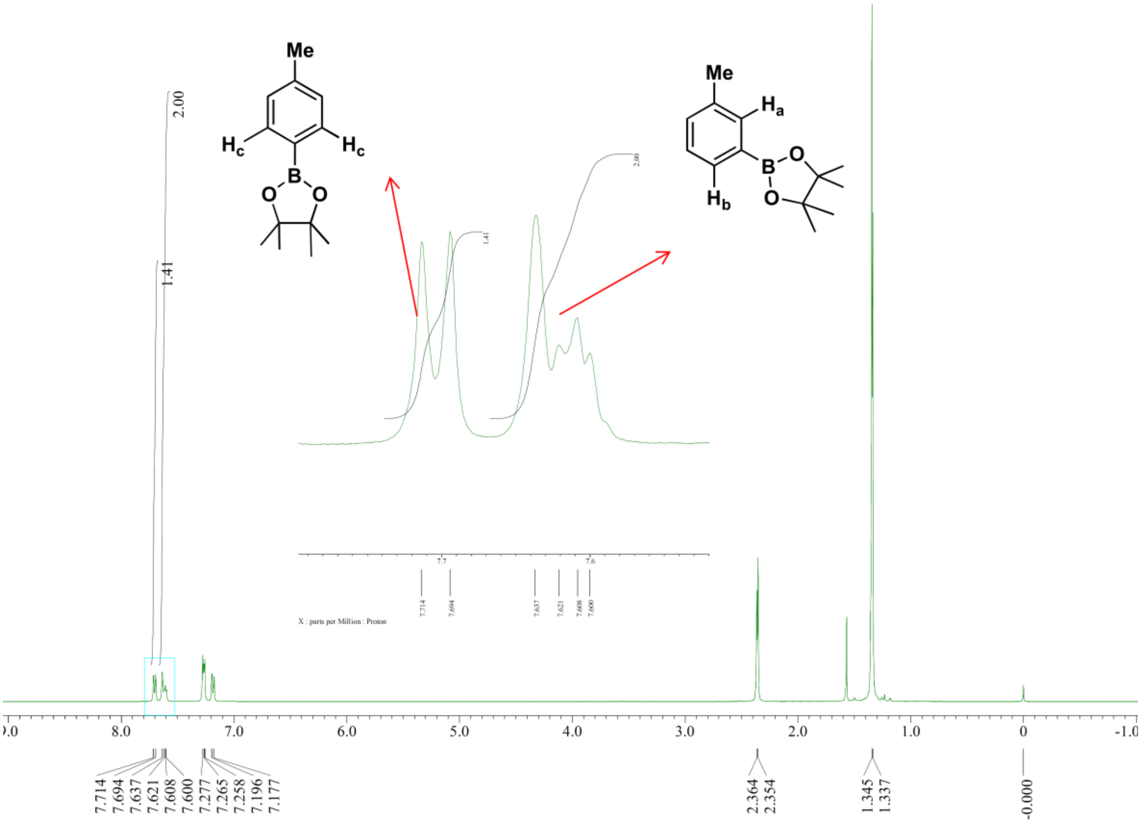

Borylation of anisole (Entry 3, Table 3). [CAS: 325142-84-5] and [CAS: 171364-79-7]

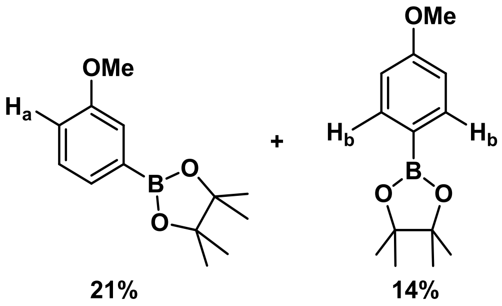

Method B was followed except that the reaction was conducted in anisole (1.0 mL). After purification by flash column chromatography over silica gel eluting with hexane/AcOEt = 20/1, a mixture of two isomers was obtained. GC/MS analysis revealed the two isomers of the borylated products had an m/z of 232 (M^+). The identity and ratio of each of the two isomers were determined by comparing the ^{1}H NMR spectrum of the product mixture with those reported in the literature.[9] The resonances specific to each isomer are as follows: ^{1}H NMR ($CDCl_3$, 399.78 MHz): 7.01 ppm (ddd, J = 0.8, 2.8, 8.0 Hz, 1H, meta isomer, H_a), 7.75 ppm (d, J = 8.2 Hz, 2H, para isomer, H_b).

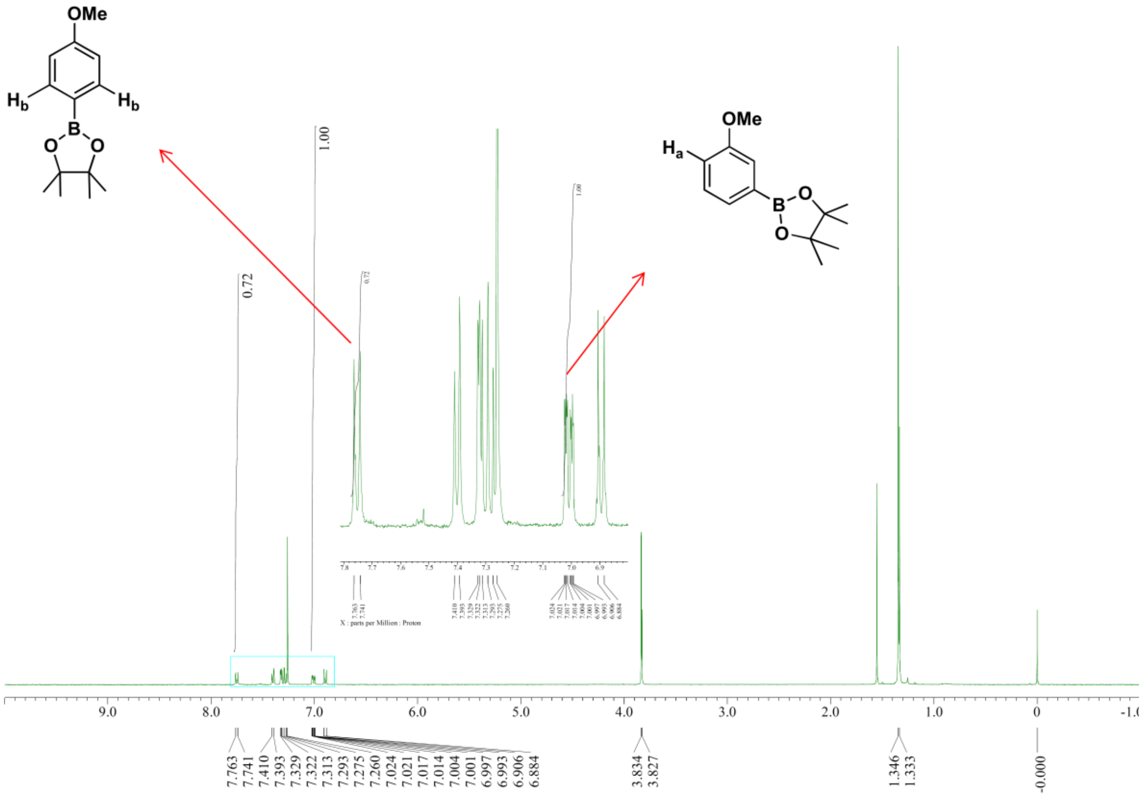

Borylation of trifuluoromethylbenzene (Entry 4, Table 3).

[CAS: 325142-82-3] and [CAS: 214360-65-3]

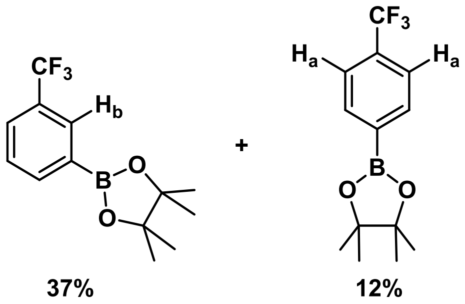

Method B was followed except that the reaction was conducted in trifluoromethylbenzene (1.0 mL). After purification by flash column chromatography over silica gel eluting with hexane/AcOEt = 40/1, a mixture of two isomers was obtained. GC/MS analysis revealed the two isomers of the borylated products had an m/z of 272 (M^+). The identity and ratio of each of the two isomers were determined by comparing the ^{1}H NMR spectrum of the product mixture with those reported in the literature.[9] The resonances specific to each isomer are as follows: ^{1}H NMR (C_6D_6, 399.78 MHz): 7.96 (d, J = 8.2 Hz, 1H, para isomer, H_a), 8.46 (s, 1H, meta isomer, H_b).

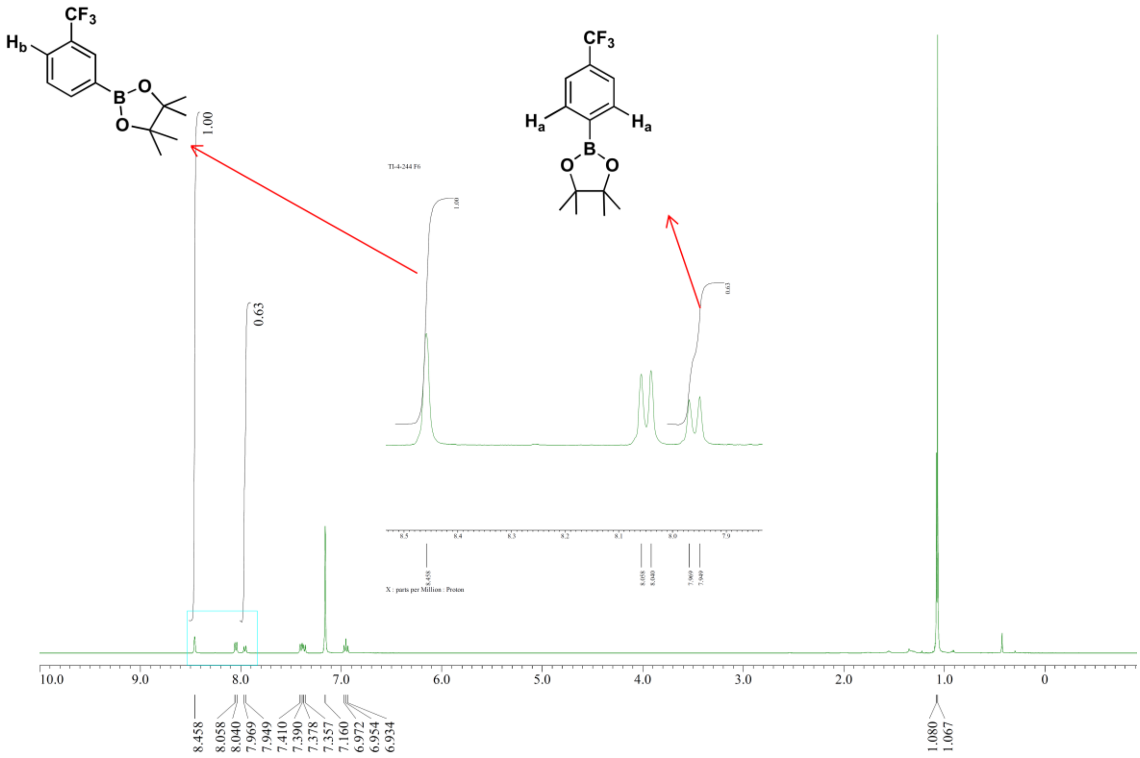

2-(3,5-Dichlorophenyl)-4,4,5,5-tetramethyl-1,3,2-dioxaborolane (21-B). [CAS: 68716-51-8]

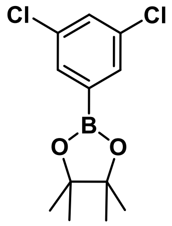

Method B was followed except that the reaction was conducted in1,3-dichlorobenzene (1.0 mL).

White solid (42 mg, 31%).

^{1}H NMR ($CDCl_3$, 399.78 MHz): δ 1.34 (s, 12H), 7.43 (t, J = 2.2 Hz, 1H), 7.64 (d, J = 2.3 Hz, 2H).

^{13}C NMR ($CDCl_3$, 100.53 MHz): δ 25.0, 84.7, 131.2, 132.9, 134.9.

HRMS (EI): Calcd for $C_{12}H_{15}BCl_2O_2$ 272.0542, Found 272.0540.

4,4,5,5-Tetramethyl-2-(naphthalen-2-yl)-1,3,2-dioxaborolane (22-B). [CAS: 256652-04-7]

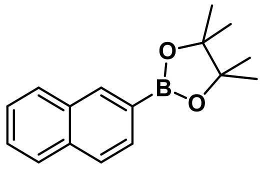

In a glovebox, $[Ir(OMe)(cod)]_2$ (33.1 mg, 0.050 mmol, 0.10 equiv), ICy·HCl (26.2 mg, 0.10 mmol, 0.20 equiv), NaO*t*-Bu (19.2 mg, 0.20 mmol, 0.40 equiv) and methylcyclohexane (1.0 mL) were added to a 10 mL-sample vial with Teflon-sealed screwcap, and stirred for 5 min at room

temperature. A naphthalene (384.1 mg, 3.0 mmol, 6.0 equiv) and **1g** (113.1 mg, 1.0 mmol, 2.0 equiv) were then added, and the cap was applied to seal the vial. The vial was stirred at 110 °C for 4 h. After the reaction mixture was cooled to room temperature, pinacol (236 mg, 2.0 mmol) in THF (2.0 mL) was added and stirred for 1.5 h at room temperature under N_2. The crude mixture was filtered through a pad of Celite eluting with AcOEt. The filtrate was concentrated in vacuo and analyzed by ^{1}H NMR using 1,2-dichloroethane as an internal standard. The crude mixture was concentrated under reduced pressure, and purified by flash column chromatography over silica gel eluting with hexane/AcOEt (40/1) solution. The filtrate was concentrated in vacuo to give a pure borylated product as a white solid (63.5 mg, 50%).

R_f 0.17 (Hexane/EtOAc = 40/1). White solid (64 mg, 50%).

^{1}H NMR (C_6D_6, 399.78 MHz): δ 1.16 (s, 12H), 7.19-7.24 (m, 2H), 7.6 (d, *J* = 8.2 Hz, 1H), 7.69 (t, *J* = 18.3, 18.3 Hz, 2H), 8.23 (d, *J* = 7.3 Hz, 1H), 8.75 (s, 1H).

^{1}H NMR ($CDCl_3$, 399.78 MHz): δ 1.40 (s, 12H), 7.47-7.53 (m, 2H), 7.82-7.83 (m, 3H), 7.89 (d, *J* = 7.8 Hz, 1H), 8.37 (s, 1H).

^{13}C NMR ($CDCl_3$, 100.53 MHz): δ 25.0, 84.0, 125.9, 127.0, 127.8, 128.7, 130.5, 132.9, 135.1, 136.3. One carbon peak is overlapped with solvent peaks.

HRMS (EI): Calcd for $C_{16}H_{19}BO_2$ 254.1478, Found 254.1482.

^{1}H NMR spectroscopic data was in agreement with the reported value.[10]

2-(5-Chloro-3-methylbenzo[*b*]thiophen-2-yl)-5,5-dimethyl-1,3,2-dioxaborinane (10-Bnep).

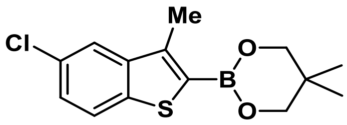

Method A was followed except that after the reaction mixture was cooled to room temperature, the neopentyl glycol (208 mg, 2.0 mmol) in THF (2.0 mL) was added and stirred for 1.5 h at room temperature under N_2.

R_f 0.085 (Hexane/EtOAc = 40/1). White Solid (130 mg, 88%). Mp = 119 °C.

^{1}H NMR (C_6D_6, 399.78 MHz): δ 0.53 (s, 6H), 2.50 (s, 3H), 3.32 (s, 4H), 7.08 (dd, *J* = 1.8, 7.8 Hz, 1H), 7.27 (d, *J* = 7.3 Hz, 1H), 7.69 (d, *J* = 2.3 Hz, 1H).

^{13}C NMR (C_6D_6, 100.53 MHz): δ 13.7, 21.5, 31.5, 72.2, 122.6, 124.0, 125.7, 130.3, 141.4, 141.6, 143.5.

IR (ATR): 2964 w, 2936 w, 1895 w, 1580 w, 1555 w, 1525 m, 1475 w, 1438 w, 1415 m, 1375 w, 1341 m, 1290 s, 1272 s, 1244 s, 1149 w, 1117 s, 1074 m, 1028 w, 977 w, 933 w, 916 w, 894 w, 865 w, 850 m, 809 s, 729 w, 697 w, 669 m.

MS m/z (% relative intensity): 296 (38), 295 (27), 294 (M^+, 100), 293 (30), 260 (12), 259 (67), 258 (21), 208 (15), 207 (14), 181 (14), 173 (15).

---

[10] Kinuta, H.; Tobisu, M.; Chatani, N. *J. Am. Chem. Soc.* **2015**, *137*, 1593.

HRMS (EI): Calcd for $C_{14}H_{16}BClO_2S$ 294.0653, Found 294.0653.

2-(5-Chloro-3-methylbenzo[*b*]thiophen-2-yl)-4,4,6-trimethyl-1,3,2-dioxaborinane (10-Bmep).

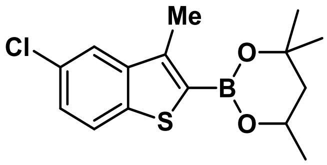

Method A was followed except that after the reaction mixture was cooled to room temperature, the 2-methylpentane-2,4-diol (236 mg, 2.0 mmol) in THF (2.0 mL) was added and stirred under N_2 at room temperature for 1.5 h.

R_f 0.23 (Hexane/EtOAc = 40/1). Colorless oil (134 mg, 87%).

^{1}H NMR (C_6D_6, 399.78 MHz): δ 1.00 (s, 3H), 1.06 (d, *J* = 6.4 Hz, 3H), 1.12-1.14 (m, 5H), 2.57 (s, 3H), 3.87-3.95 (m, 1H), 7.08 (dd, *J* = 1.8, 8.5 Hz, 1H), 7.28 (d, *J* = 8.4 Hz, 1H), 7.70 (d, *J* = 1.8 Hz, 1H).

^{13}C NMR (C_6D_6, 100.53 MHz): δ 13.6, 23.0, 28.0, 31.1, 45.7, 65.5, 71.7, 122.5, 123.9, 125.6, 130.3, 141.1, 141.3, 143.5.

IR (ATR): 2973 w, 2914 w, 2360 w, 2340 w, 1737 w, 1581 w, 1554 w, 1523 w, 1440 w, 1396 m, 1379 w, 1344 m, 1319 w, 1286 s, 1265 s, 1243 s, 1206 m, 1160 m, 1109 m, 1077 m, 1059 w, 1027 w, 980 w, 963 w, 937 w, 901 w, 864 w, 851 w, 823 w, 799 m, 768 m, 730 w, 692 w, 972 m.

MS m/z (% relative intensity): 310 (38), 309 (26), 308 (M^+, 100), 307 (24), 254 (13), 252 (35), 251 (13), 237 (16), 225 (11), 211 (18), 210 (42), 209 (57), 208 (97), 207 (24), 182 (11), 181 (18), 173 (26), 83 (21), 55 (10), 43 (26).

HRMS (EI): Calcd for $C_{15}H_{18}BClO_2S$ 308.0809, Found 308.0804.

2-(5-Chloro-3-methylbenzo[*b*]thiophen-2-yl)-2,3-dihydro-1*H*-naphtho[1,8-*de*][1,3,2]diazaborinine (10-Bdan).

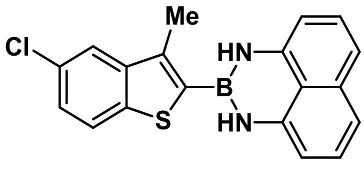

Method A was followed except that after the reaction mixture was cooled to room temperature, the 1,8-naphthalenediamine (316 mg, 2.0 mmol) in THF (2.0 mL) was added and stirred for 1.5 h at room temperature under N_2.

R_f 0.29 (Hexane/EtOAc = 20/1). White solid (131 mg, 75%). Mp = 173 °C.

^{1}H NMR (C_6D_6, 399.78 MHz): δ 1.97 (s, 3H), 5.32 (s, 2H), 5.92 (dd, *J* = 0.92, 7.3 Hz, 2H), 7.02-7.15 (m, 5H), 7.33 (d, *J* = 8.2 Hz, 1H). 7.65 (d, *J* = 1.8 Hz, 1H).

^{13}C NMR (C_6D_6, 100.53 MHz): δ 13.9, 106.8, 118.8, 120.6, 122.2, 123.8, 125.5, 130.9, 136.9, 137.0,

140.3, 140.8, 143.0.

IR (ATR): 3428 w, 3415 w, 3049 w, 2360 w, 1734 w, 1627 w, 1596 s, 1554 w, 1528 m, 1497 m, 1437 w, 1405 m, 1371 m, 1337 m, 1281 w, 1195 m, 1164 m, 1099 m, 1074 m, 1035 w, 935 w, 859 m, 814 m, 798 m, 751 s, 660 s.

MS m/z (% relative intensity): 350 (42), 349 (32), 348 (M^+, 100), 347 (29), 174 (15), 173 (14), 166 (38), 165 (21).

HRMS (EI): Calcd for $C_{19}H_{14}BClN_2S$348.0659, Found 348.0662.

Borylation of other substrates.

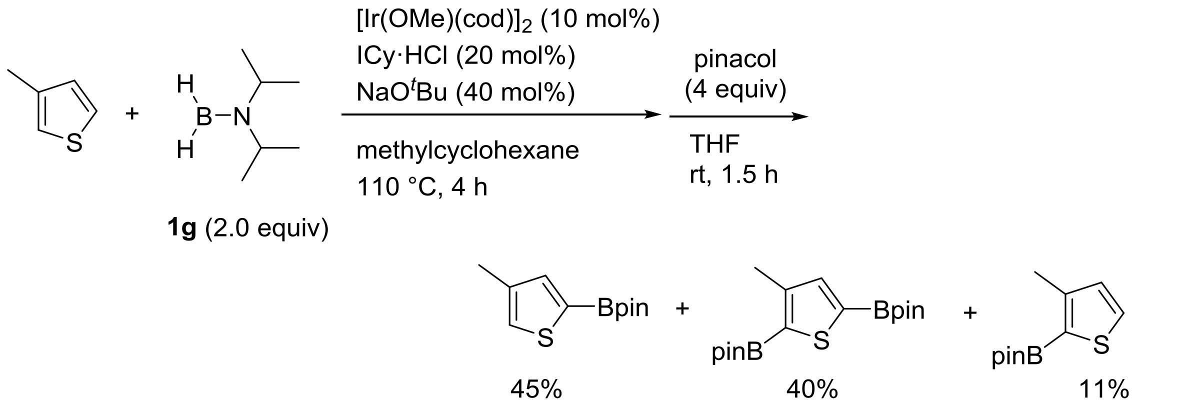

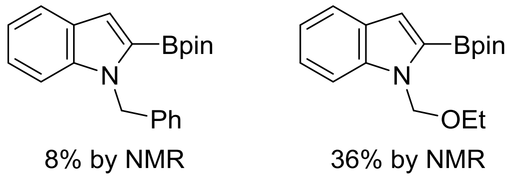

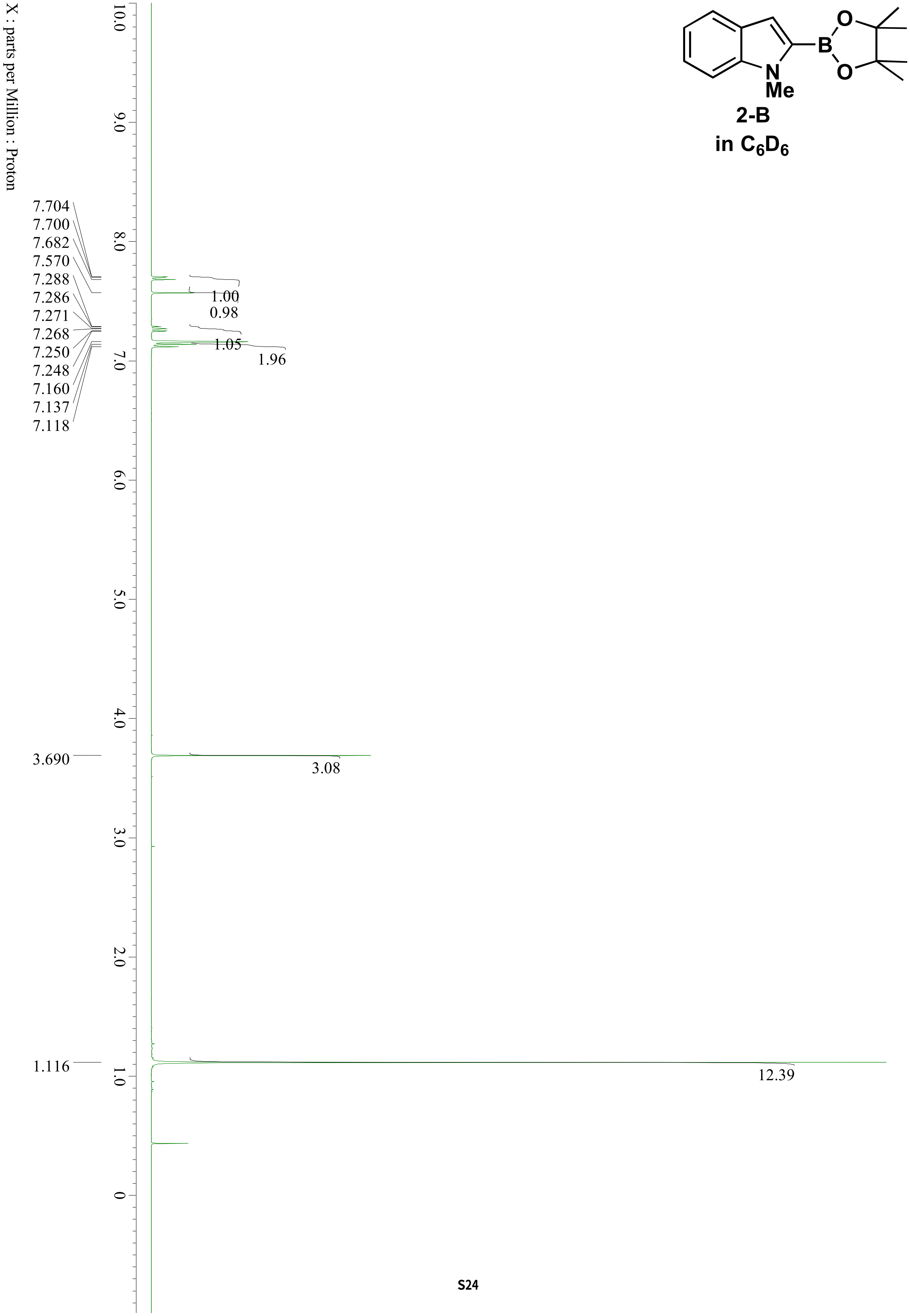

2-B

in C_6D_6

X : parts per Million : Proton
10.0
9.0
8.0
7.0
6.0
5.0
4.0
3.0
2.0
1.0
0
7.704
7.700
7.682
7.570
7.288
7.286
7.271
7.268
7.250
7.248
7.160
7.137
7.118
3.690
1.116
1.00
0.98
1.05
1.96
3.08
12.39

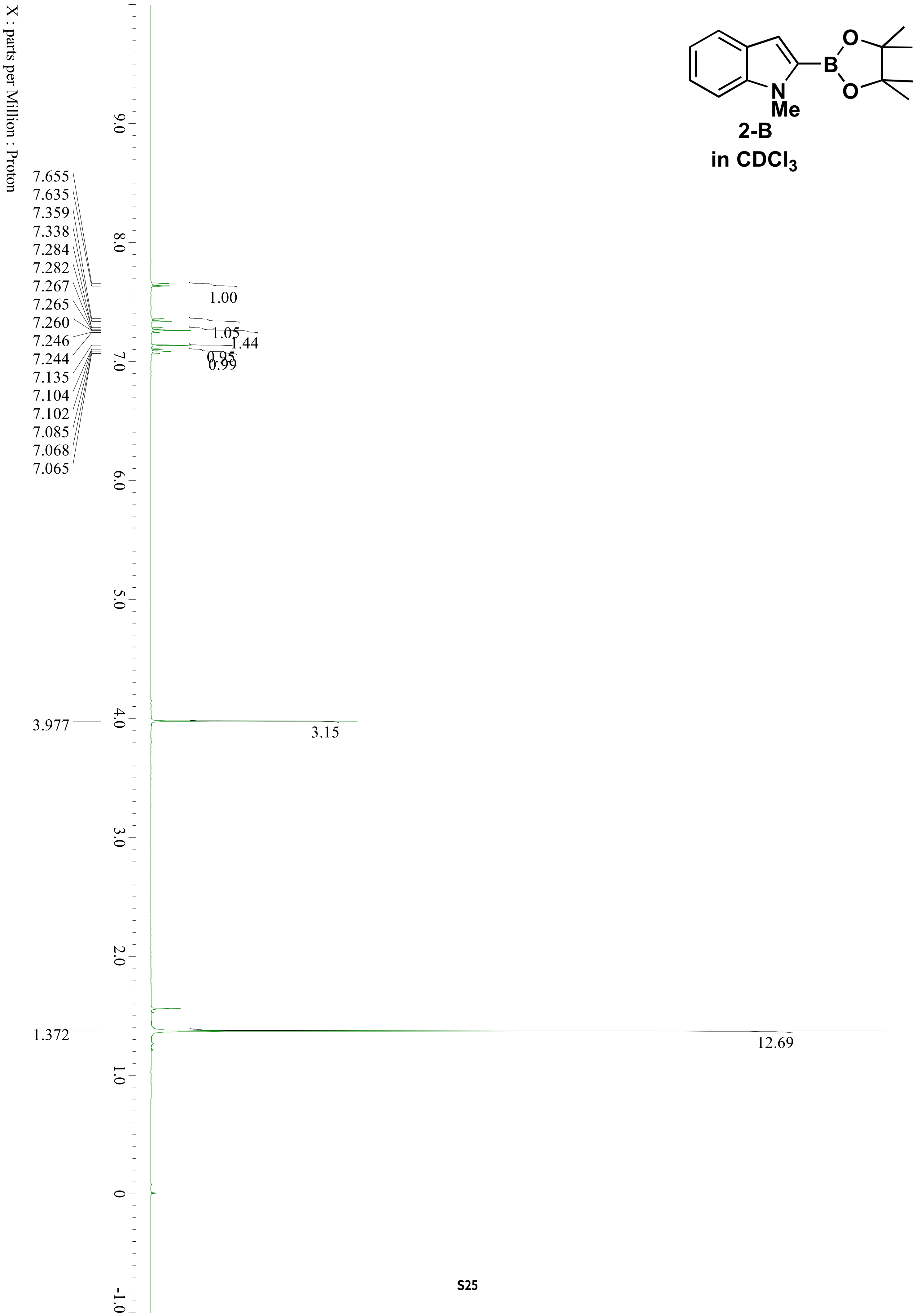

X : parts per Million : Proton
7.655
7.635
7.359
7.338
7.284
7.282
7.267
7.265
7.260
7.246
7.244
7.135
7.104
7.102
7.085
7.068
7.065
3.977
1.372
9.0
8.0
7.0
6.0
5.0
4.0
3.0
2.0
1.0
0
-1.0
1.00
1.05
1.44
0.95
0.99
3.15
12.69
O
B
O
N
Me

2-B
in $CDCl_3$

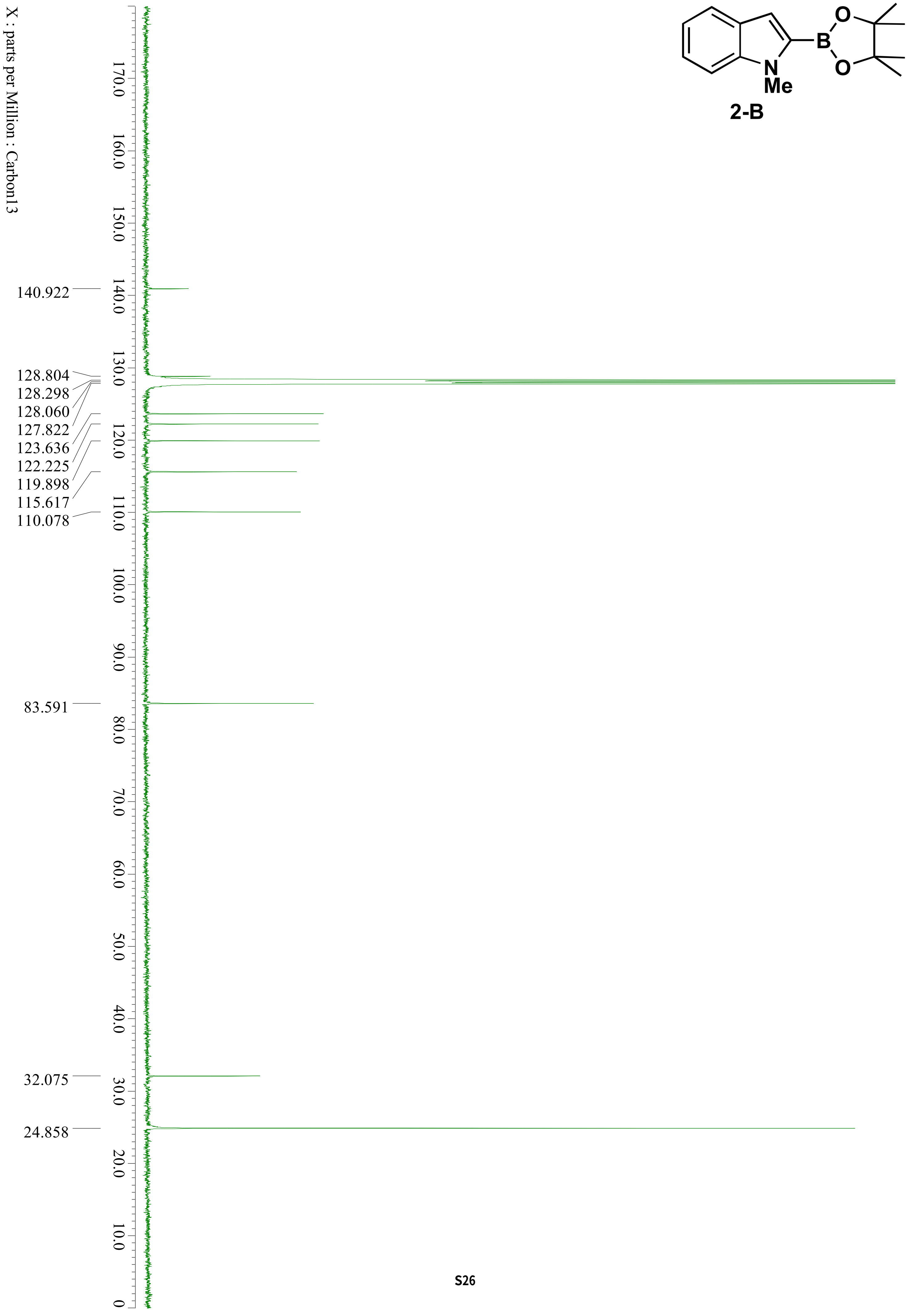

2-B
X : parts per Million : Carbon13
140.922
128.804
128.298
128.060
127.822
123.636
122.225
119.898
115.617
110.078
83.591
32.075
24.858
170.0
160.0
150.0
140.0
130.0
120.0
110.0
100.0
90.0
80.0
70.0
60.0
50.0
40.0
30.0
20.0
10.0
0

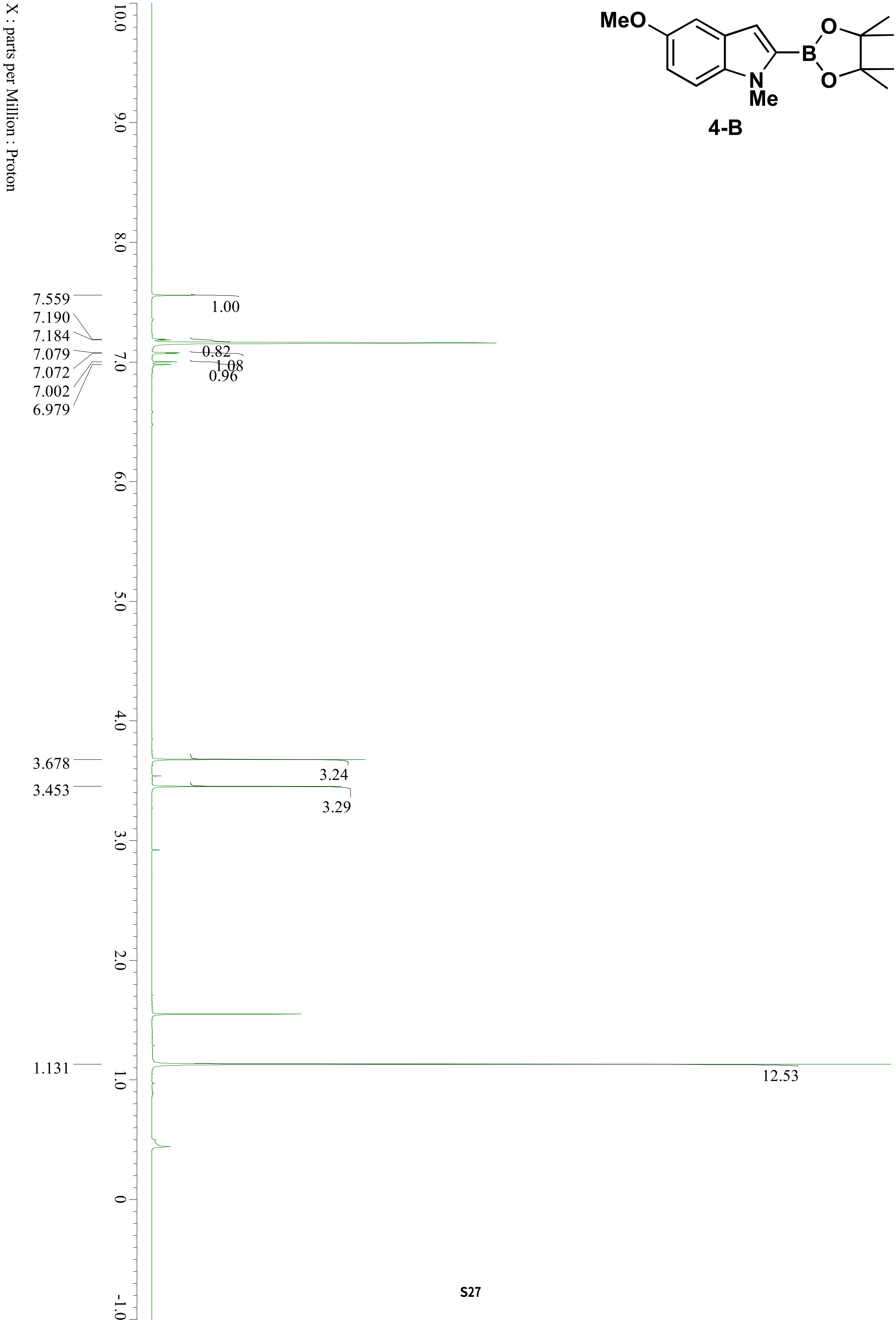
MeO
N
Me
B
O
O
4-B
X : parts per Million : Proton
7.559
7.190
7.184
7.079
7.072
7.002
6.979
3.678
3.453
1.131
1.00
0.82
1.08
0.96
3.24
3.29
12.53

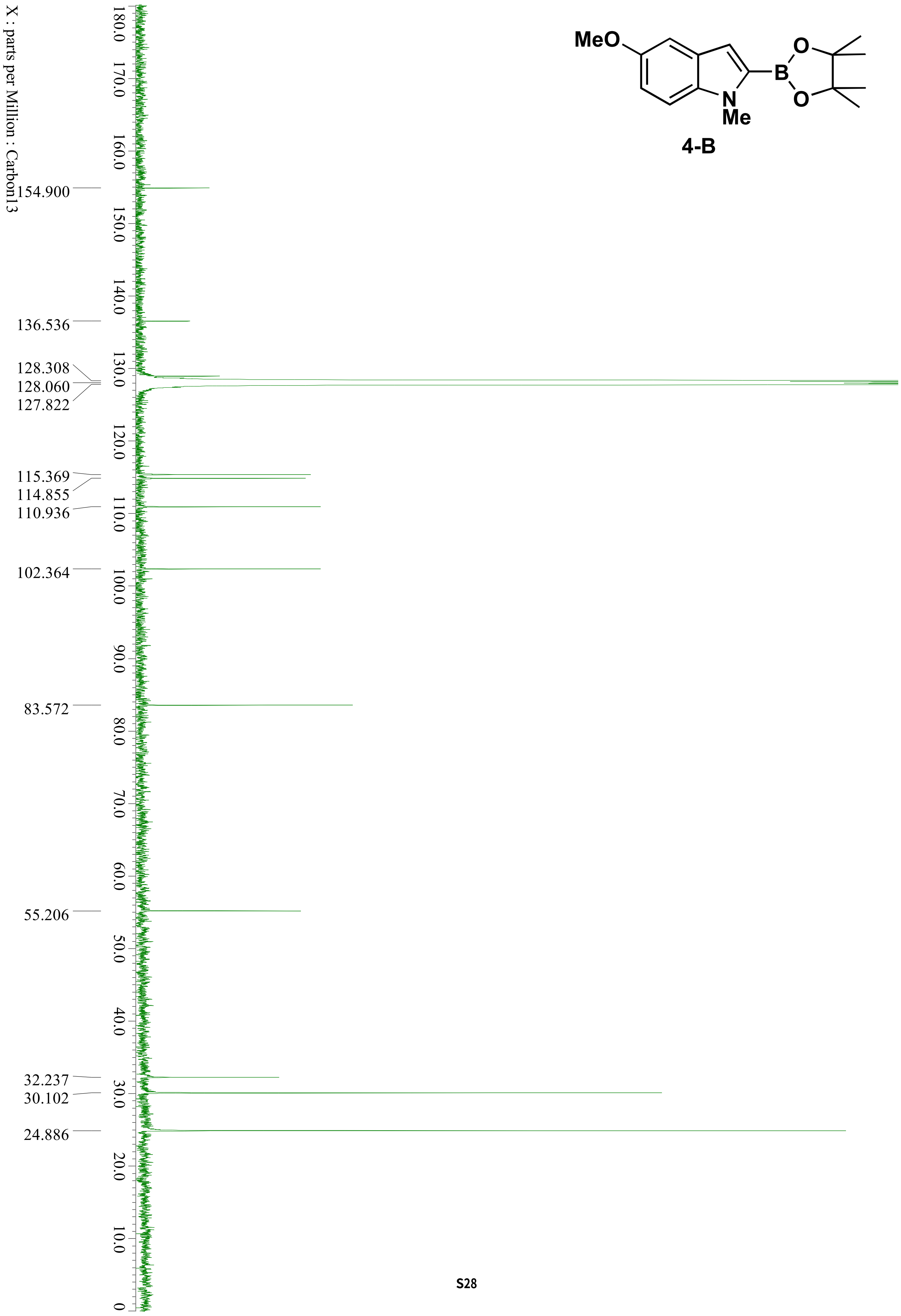
MeO
N
Me
B
O
O
4-B
X : parts per Million : Carbon13
154.900
136.536
128.308
128.060
127.822
115.369
114.855
110.936
102.364
83.572
55.206
32.237
30.102
24.886
180.0
170.0
160.0
150.0
140.0
130.0
120.0
110.0
100.0
90.0
80.0
70.0
60.0
50.0
40.0
30.0
20.0
10.0
0

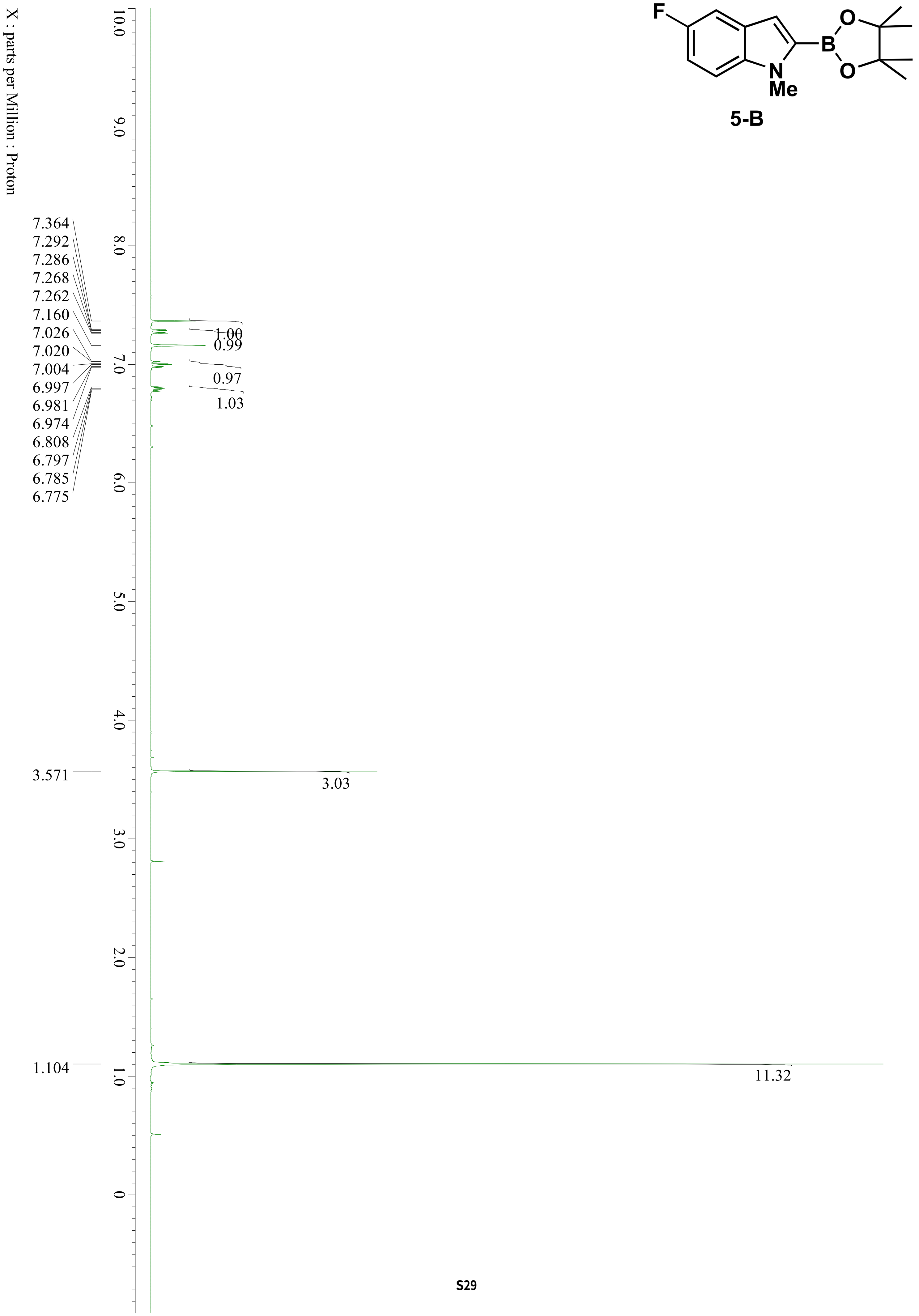
5-B
X : parts per Million : Proton
7.364
7.292
7.286
7.268
7.262
7.160
7.026
7.020
7.004
6.997
6.981
6.974
6.808
6.797
6.785
6.775
3.571
1.104
1.00
0.99
0.97
1.03
3.03
11.32
10.0
9.0
8.0
7.0
6.0
5.0
4.0
3.0
2.0
1.0
0

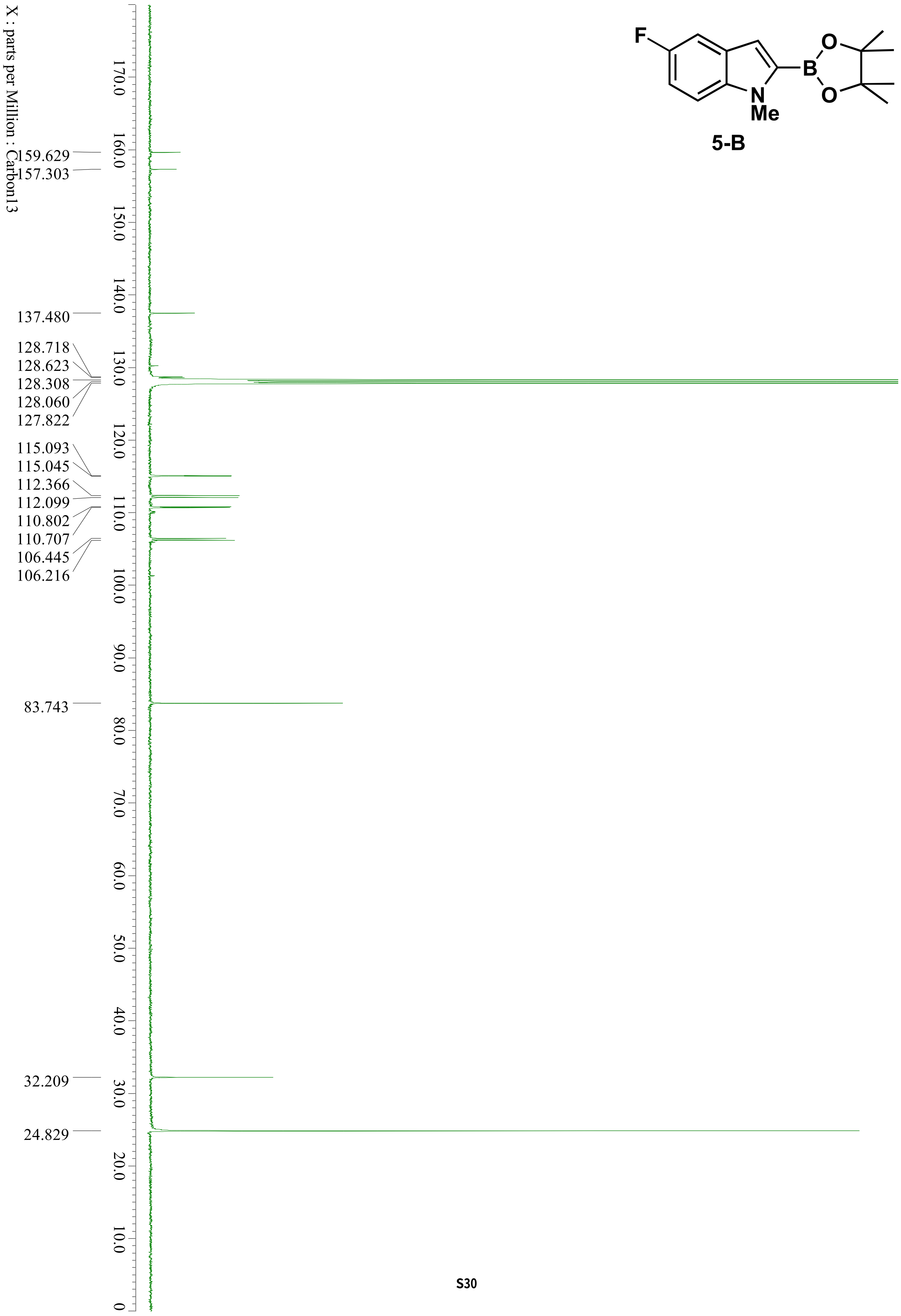
X : parts per Million : Carbon13
159.629
157.303
137.480
128.718
128.623
128.308
128.060
127.822
115.093
115.045
112.366
112.099
110.802
110.707
106.445
106.216
83.743
32.209
24.829
F
N
Me
B
O
O
5-B

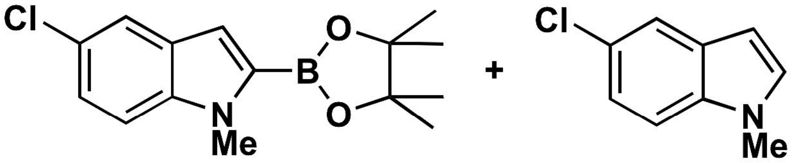

6-B + **6**

(6-B:6 = 9:1)

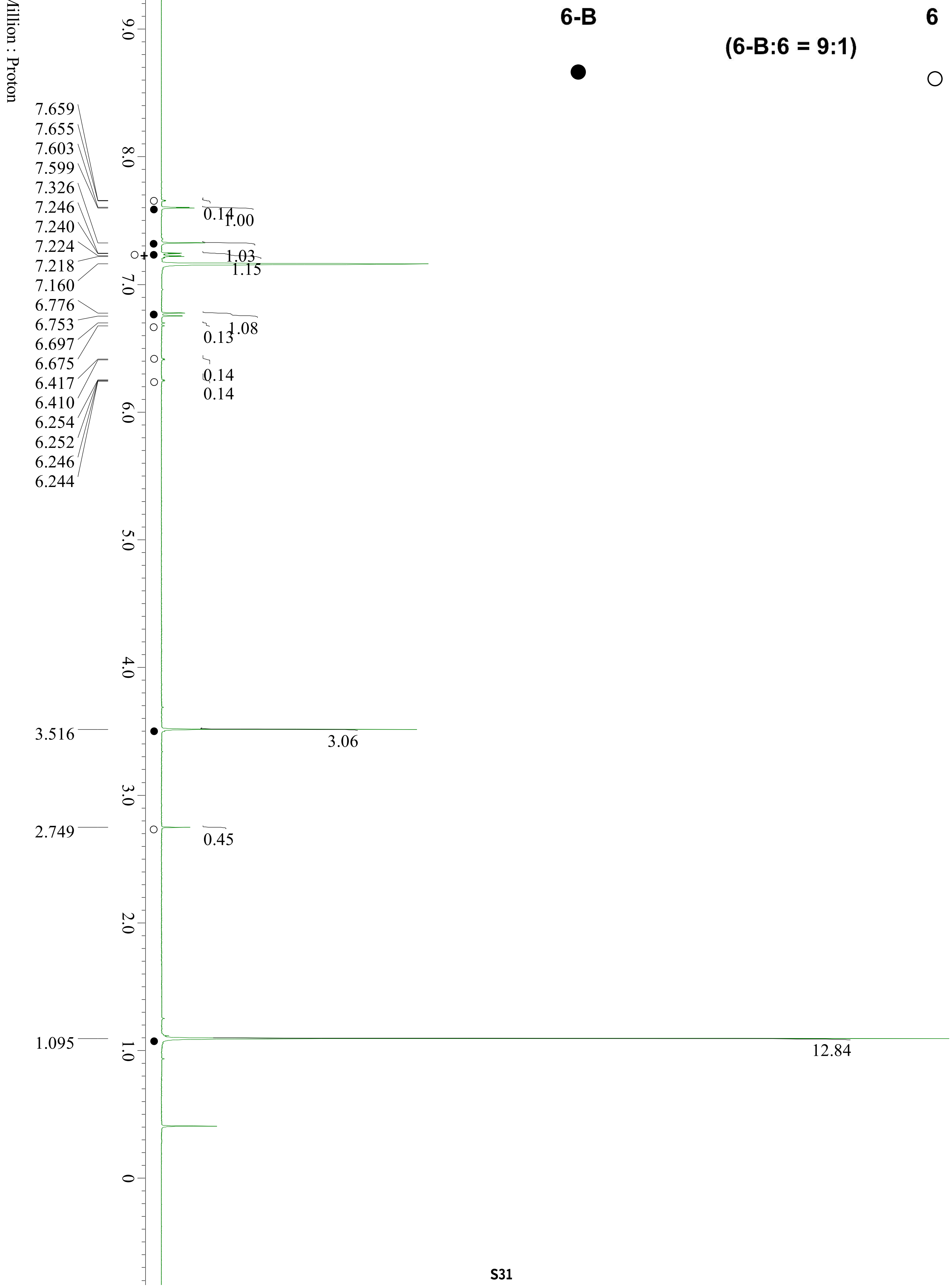
X : parts per Million : Proton
10.0
9.0
8.0
7.0
6.0
5.0
4.0
3.0
2.0
1.0
0
7.659
7.655
7.603
7.599
7.326
7.246
7.240
7.224
7.218
7.160
6.776
6.753
6.697
6.675
6.417
6.410
6.254
6.252
6.246
6.244
3.516
2.749
1.095
0.14
1.00
1.03
1.15
1.08
0.13
0.14
0.14
3.06
0.45
12.84

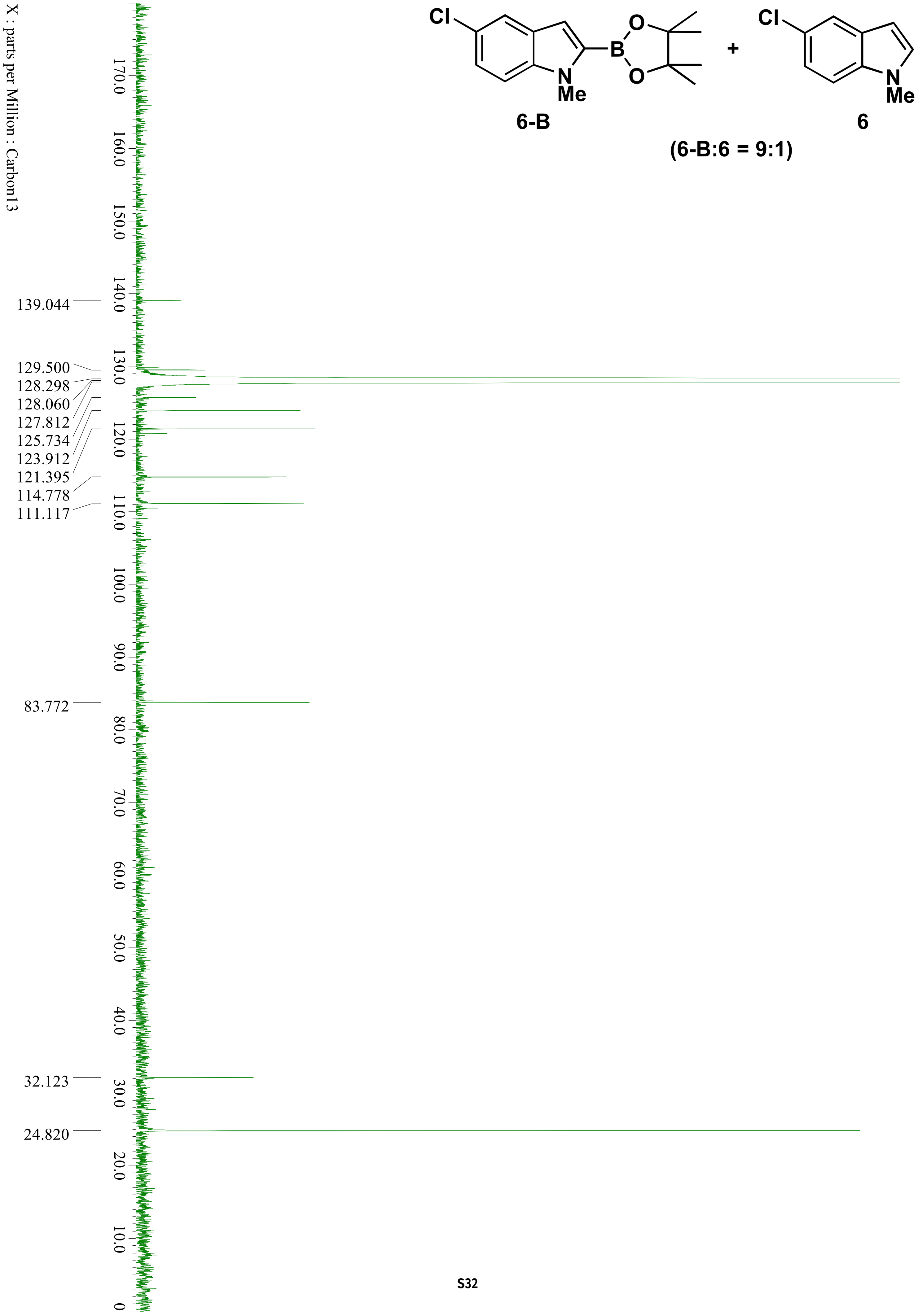
Cl
O
B
O
N
Me
+
Cl
N
Me
6-B
6
(6-B:6 = 9:1)
X : parts per Million : Carbon13
139.044
129.500
128.298
128.060
127.812
125.734
123.912
121.395
114.778
111.117
83.772
32.123
24.820
170.0
160.0
150.0
140.0
130.0
120.0
110.0
100.0
90.0
80.0
70.0
60.0
50.0
40.0
30.0
20.0
10.0
0

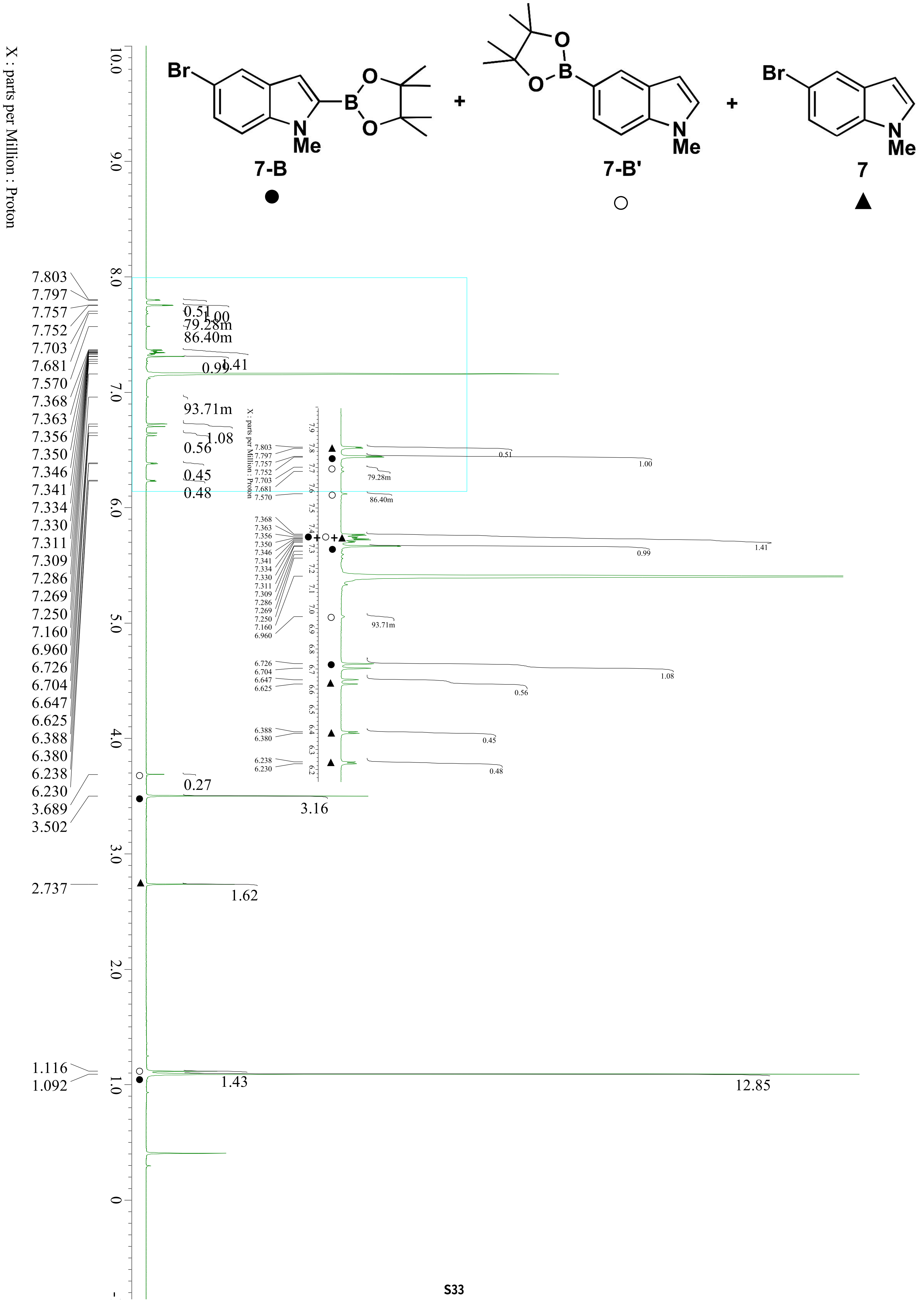
Br
N
Me
B
O
O
7-B
+
7-B'
7
X : parts per Million : Proton
7.803
7.797
7.757
7.752
7.703
7.681
7.570
7.368
7.363
7.356
7.350
7.346
7.341
7.334
7.330
7.311
7.309
7.286
7.269
7.250
7.160
6.960
6.726
6.704
6.647
6.625
6.388
6.380
6.238
6.230
3.689
3.502
2.737
1.116
1.092
0.51
1.00
79.28m
86.40m
0.99
1.41
93.71m
1.08
0.56
0.45
0.48
0.27
3.16
1.62
1.43
12.85

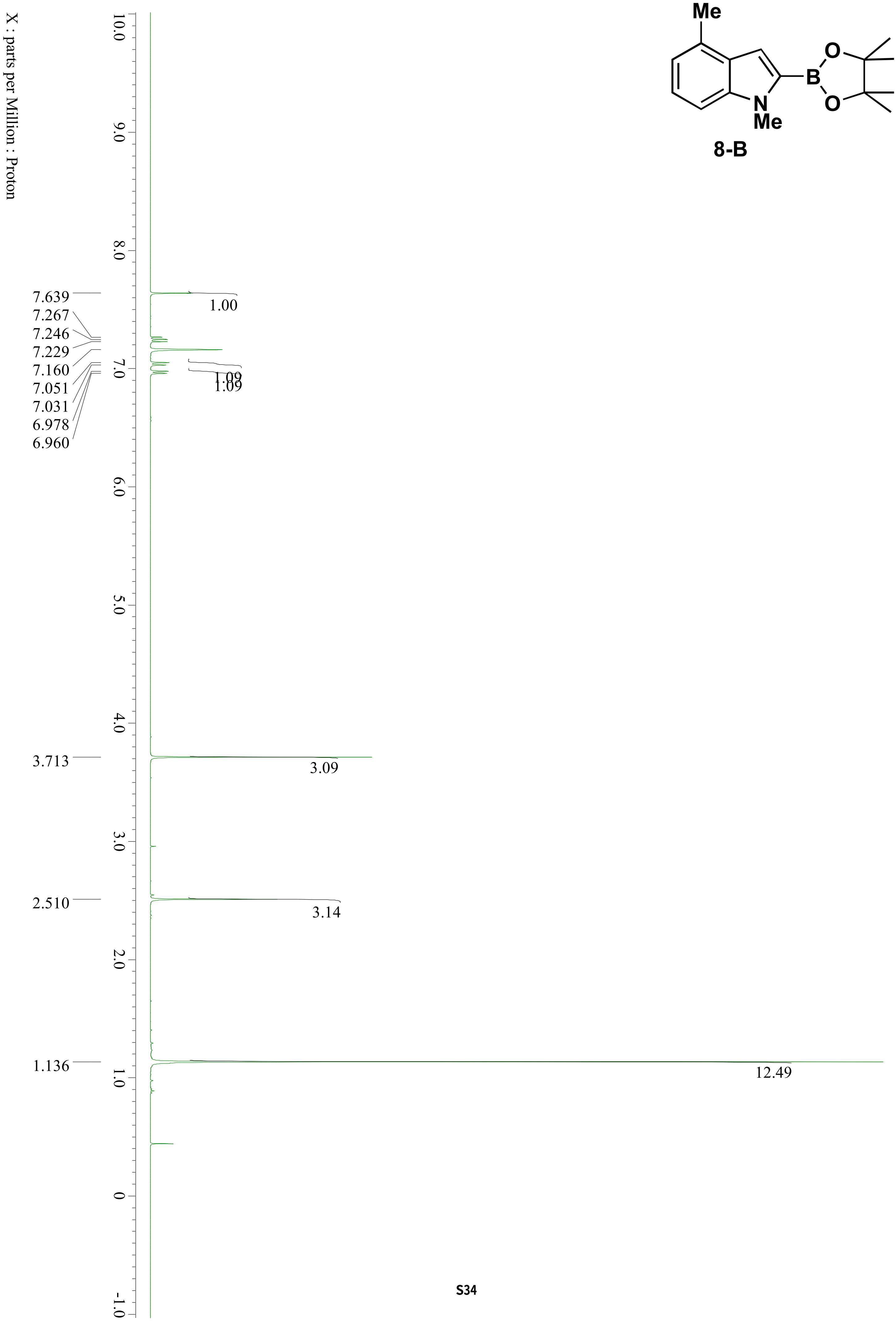
X : parts per Million : Proton
10.0
9.0
8.0
7.0
6.0
5.0
4.0
3.0
2.0
1.0
0
-1.0
7.639
7.267
7.246
7.229
7.160
7.051
7.031
6.978
6.960
3.713
2.510
1.136
1.00
1.09
1.09
3.09
3.14
12.49
Me
O
B
O
N
Me

8-B

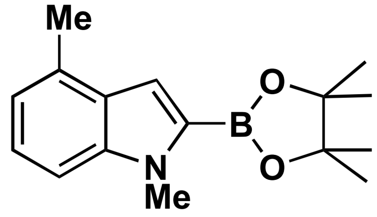
Me
O
B
O
N
Me

8-B

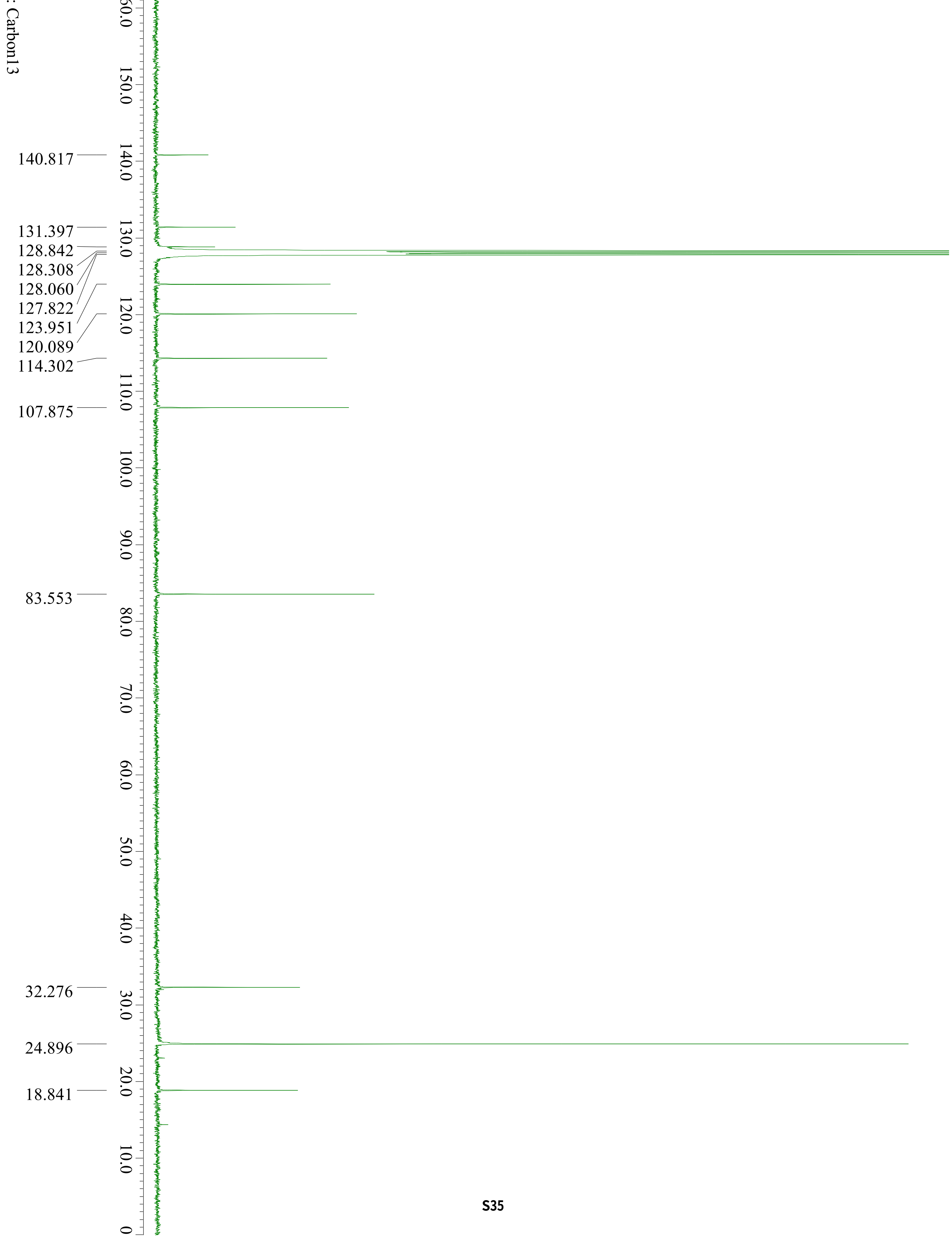
X : parts per Million : Carbon13
140.817
131.397
128.842
128.308
128.060
127.822
123.951
120.089
114.302
107.875
83.553
32.276
24.896
18.841
170.0
160.0
150.0
140.0
130.0
120.0
110.0
100.0
90.0
80.0
70.0
60.0
50.0
40.0
30.0
20.0
10.0
0

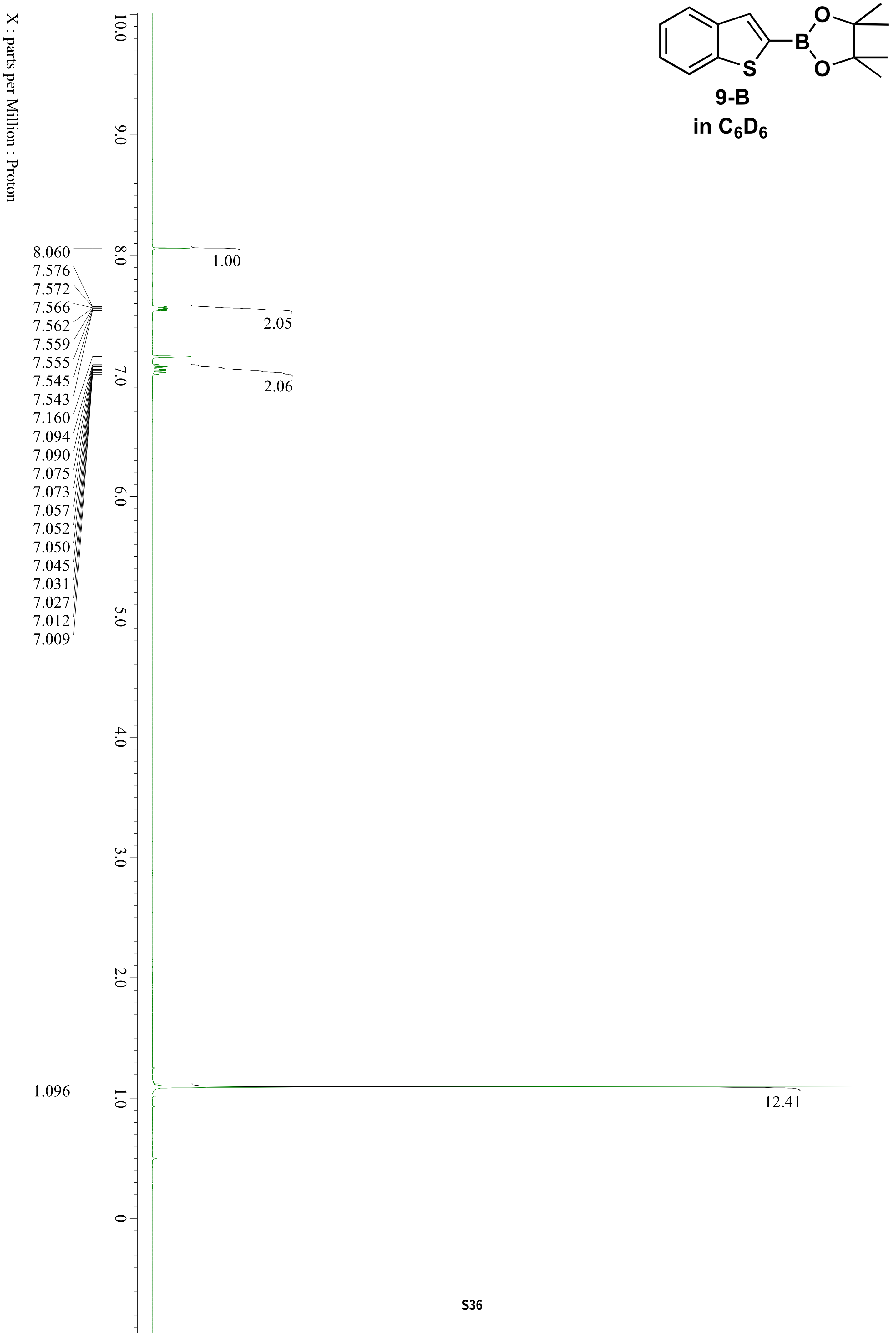

9-B
in C_6D_6

X : parts per Million : Proton
10.0
9.0
8.0
7.0
6.0
5.0
4.0
3.0
2.0
1.0
0
8.060
7.576
7.572
7.566
7.562
7.559
7.555
7.545
7.543
7.160
7.094
7.090
7.075
7.073
7.057
7.052
7.050
7.045
7.031
7.027
7.012
7.009
1.096
1.00
2.05
2.06
12.41

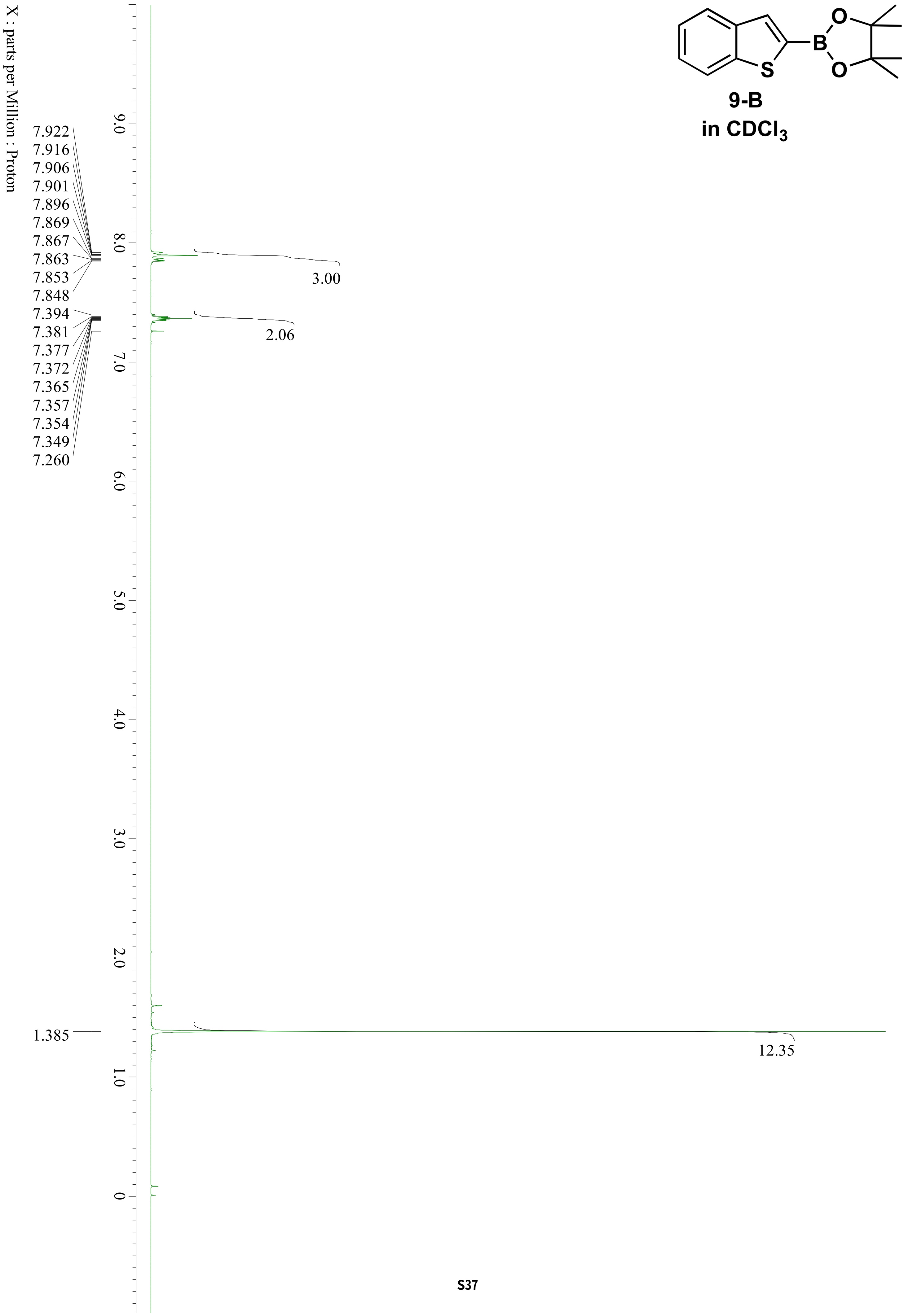
X : parts per Million : Proton
7.922
7.916
7.906
7.901
7.896
7.869
7.867
7.863
7.853
7.848
7.394
7.381
7.377
7.372
7.365
7.357
7.354
7.349
7.260
1.385
9.0
8.0
7.0
6.0
5.0
4.0
3.0
2.0
1.0
0
3.00
2.06
12.35
B
O
O
S

9-B
in $CDCl_3$

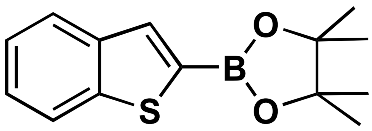

9-B

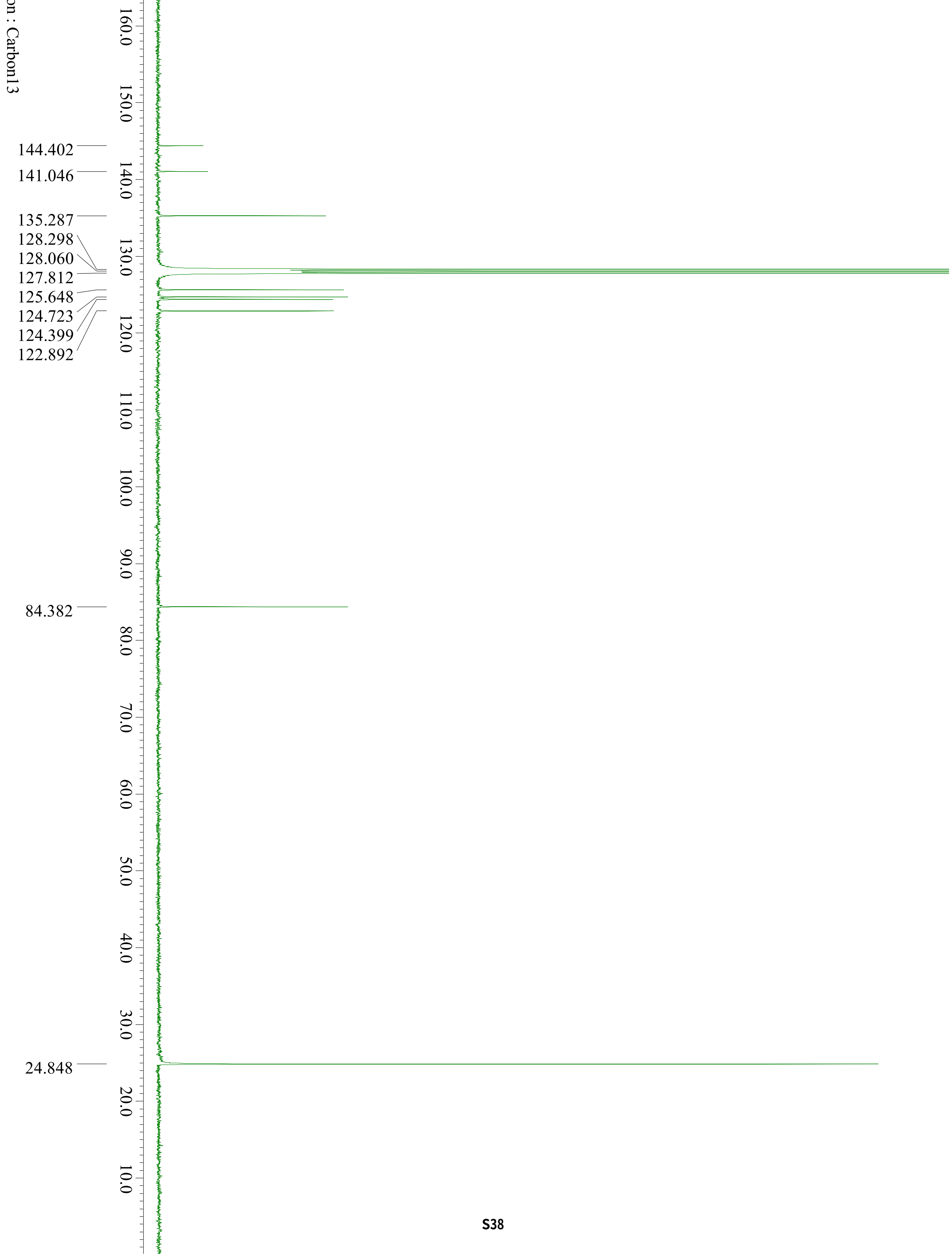
X : parts per Million : Carbon13
144.402
141.046
135.287
128.298
128.060
127.812
125.648
124.723
124.399
122.892
84.382
24.848
170.0
160.0
150.0
140.0
130.0
120.0
110.0
100.0
90.0
80.0
70.0
60.0
50.0
40.0
30.0
20.0
10.0

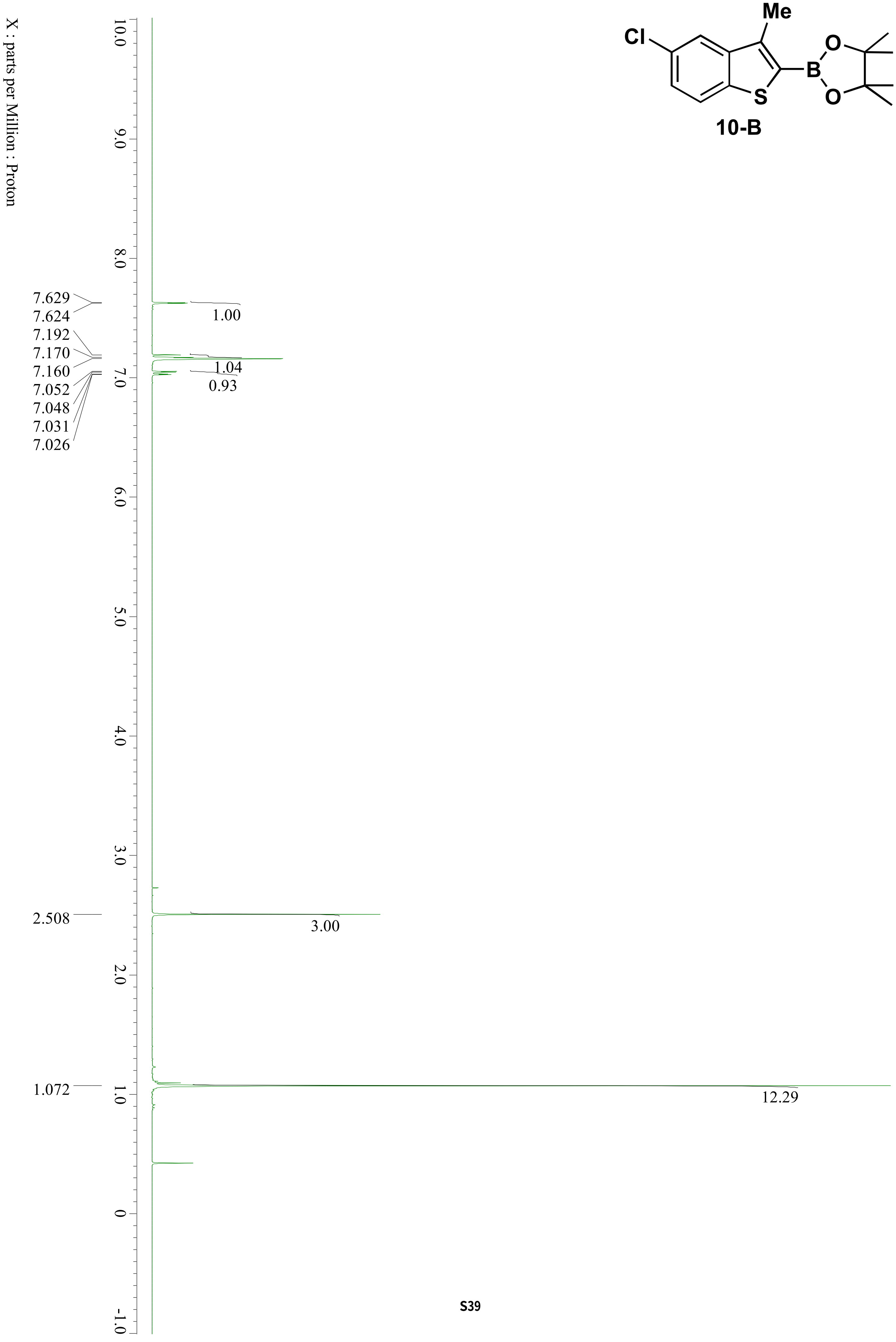
10-B
X : parts per Million : Proton
7.629
7.624
7.192
7.170
7.160
7.052
7.048
7.031
7.026
2.508
1.072
1.00
1.04
0.93
3.00
12.29
10.0
9.0
8.0
7.0
6.0
5.0
4.0
3.0
2.0
1.0
0
-1.0

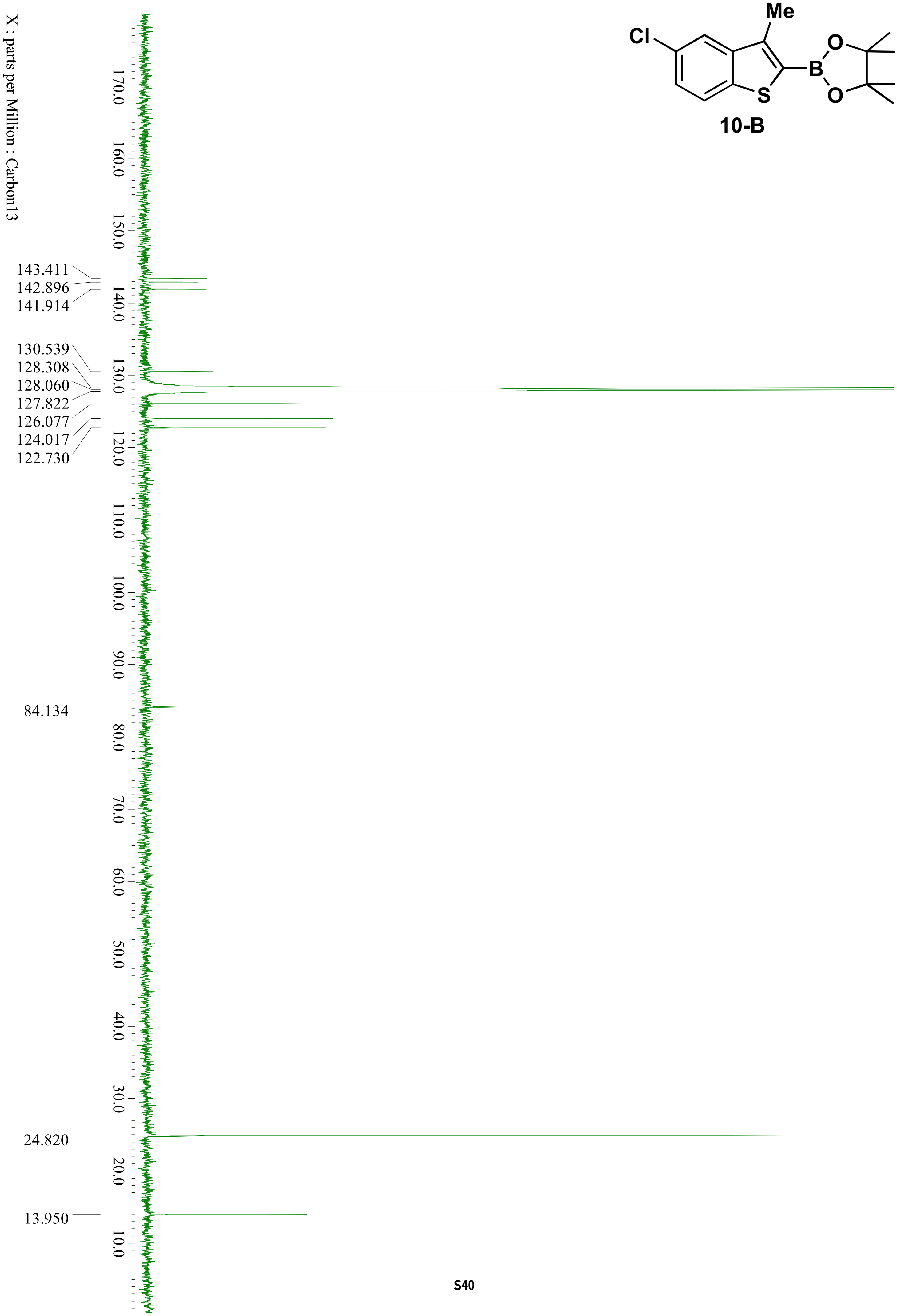
X : parts per Million : Carbon13
170.0
160.0
150.0
140.0
130.0
120.0
110.0
100.0
90.0
80.0
70.0
60.0
50.0
40.0
30.0
20.0
10.0
143.411
142.896
141.914
130.539
128.308
128.060
127.822
126.077
124.017
122.730
84.134
24.820
13.950
Me
Cl
S
B
O
O

10-B

11-B
in C_6D_6

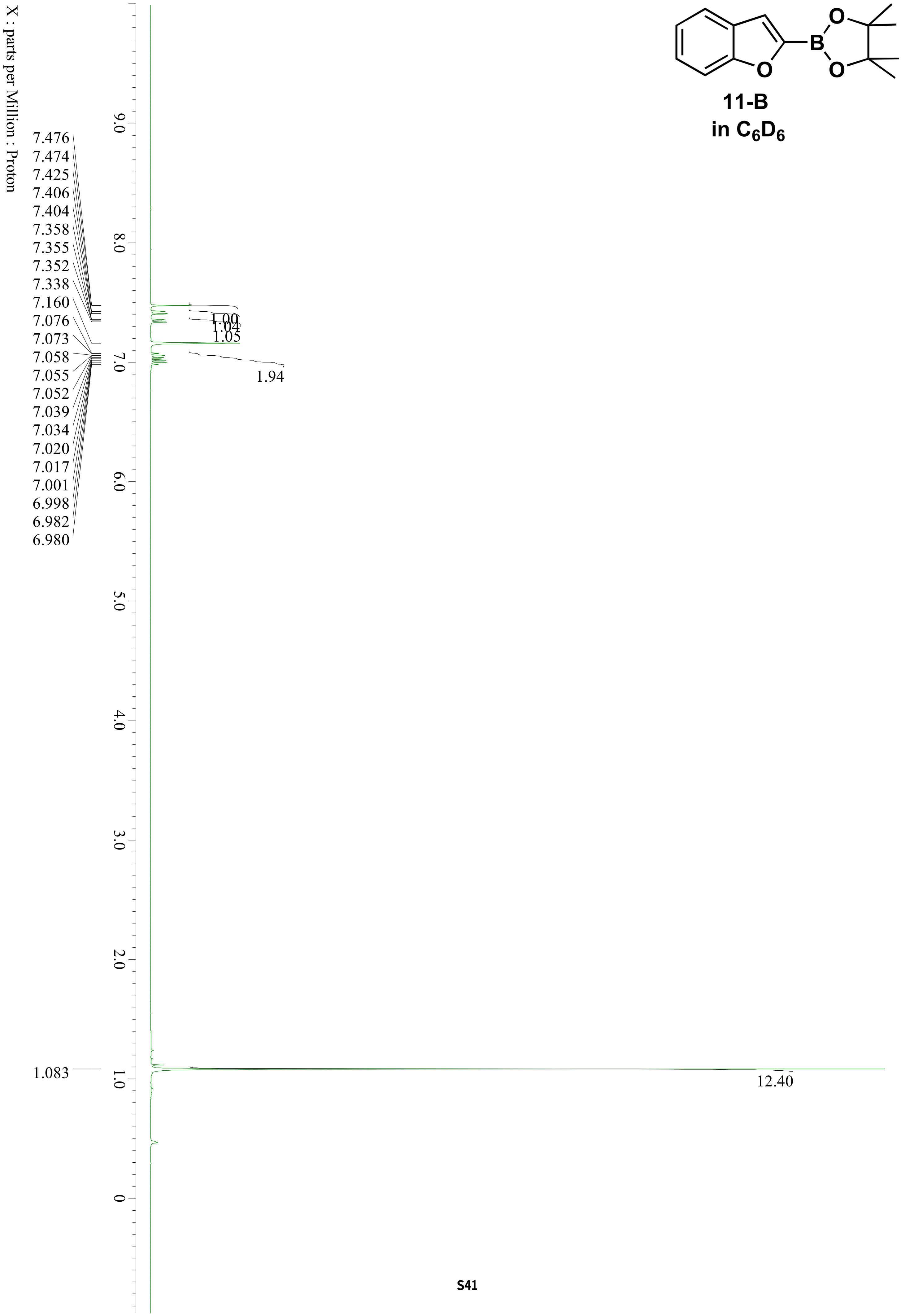
X : parts per Million : Proton
7.476
7.474
7.425
7.406
7.404
7.358
7.355
7.352
7.338
7.160
7.076
7.073
7.058
7.055
7.052
7.039
7.034
7.020
7.017
7.001
6.998
6.982
6.980
1.083
9.0
8.0
7.0
6.0
5.0
4.0
3.0
2.0
1.0
0
1.00
1.04
1.05
1.94
12.40

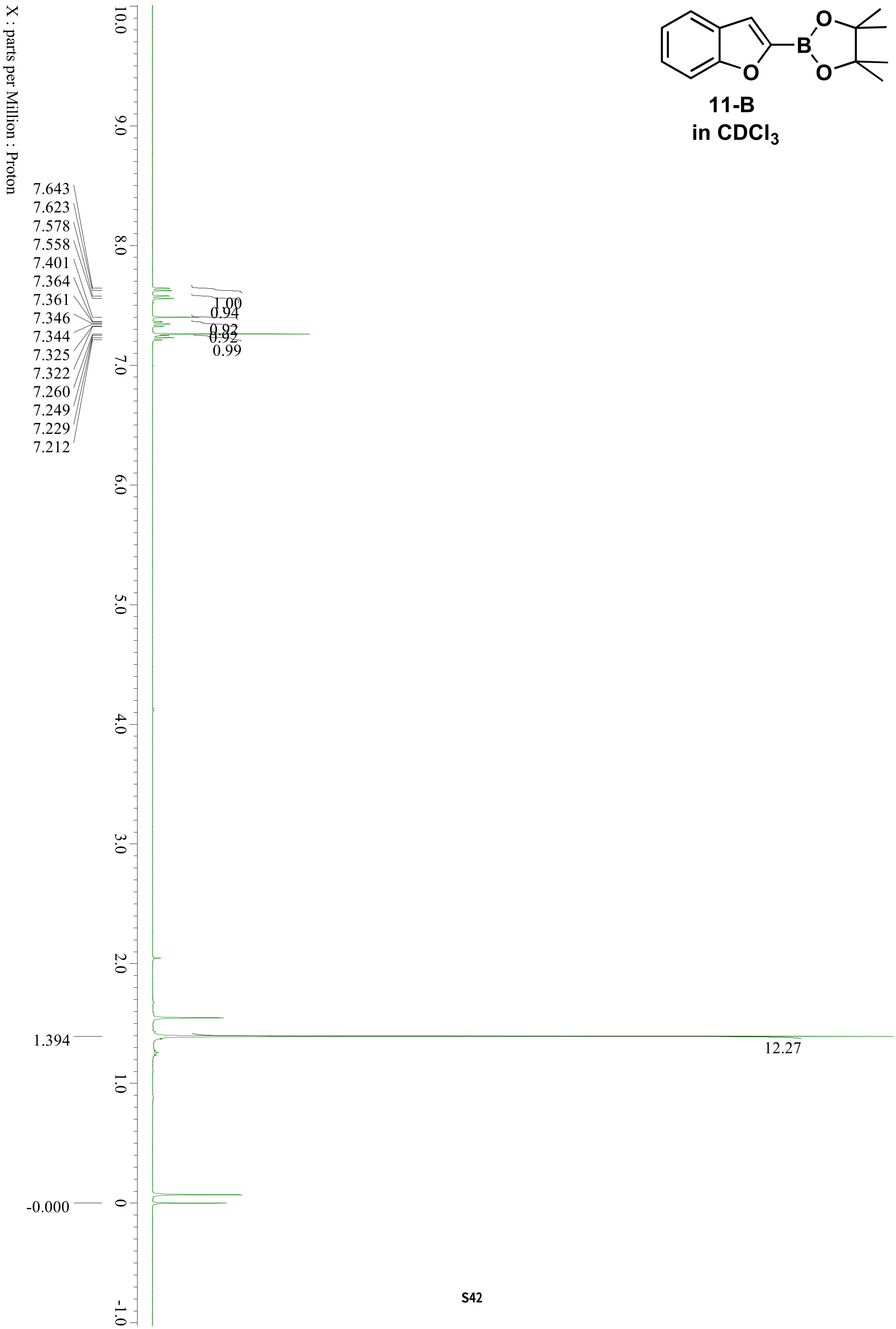
X : parts per Million : Proton
7.643
7.623
7.578
7.558
7.401
7.364
7.361
7.346
7.344
7.325
7.322
7.260
7.249
7.229
7.212
1.394
-0.000
1.00
0.94
0.92
0.92
0.99
12.27
10.0
9.0
8.0
7.0
6.0
5.0
4.0
3.0
2.0
1.0
0
-1.0

11-B
in $CDCl_3$

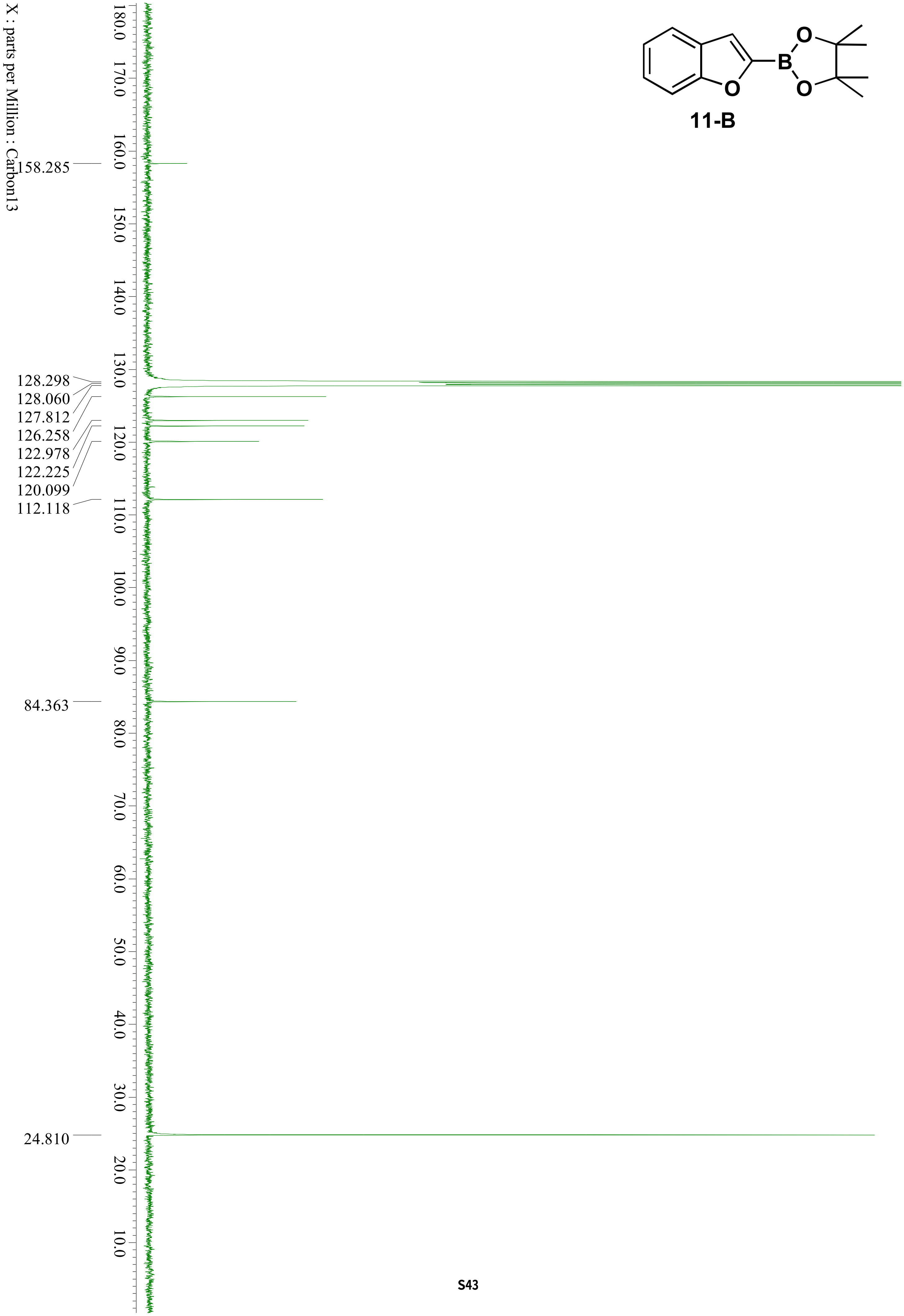
X : parts per Million : Carbon13
158.285
128.298
128.060
127.812
126.258
122.978
122.225
120.099
112.118
84.363
24.810
180.0 170.0 160.0 150.0 140.0 130.0 120.0 110.0 100.0 90.0 80.0 70.0 60.0 50.0 40.0 30.0 20.0 10.0

11-B

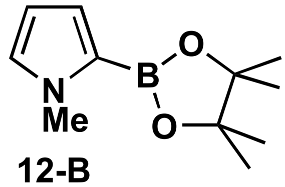

12-B

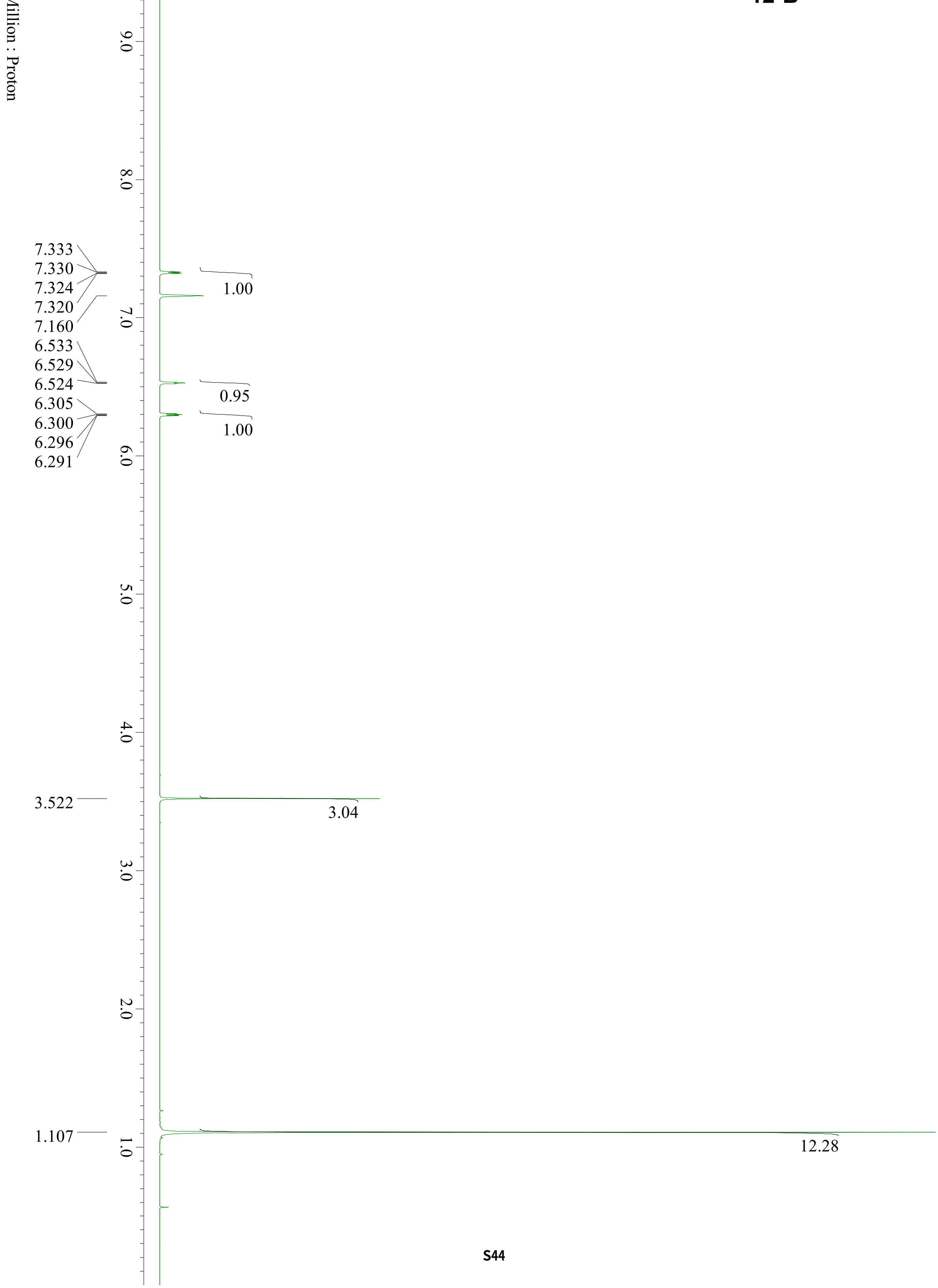
X : parts per Million : Proton
10.0
9.0
8.0
7.0
6.0
5.0
4.0
3.0
2.0
1.0
7.333
7.330
7.324
7.320
7.160
6.533
6.529
6.524
6.305
6.300
6.296
6.291
3.522
1.107
1.00
0.95
1.00
3.04
12.28

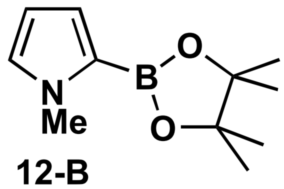

N
Me
B
O
O

12-B

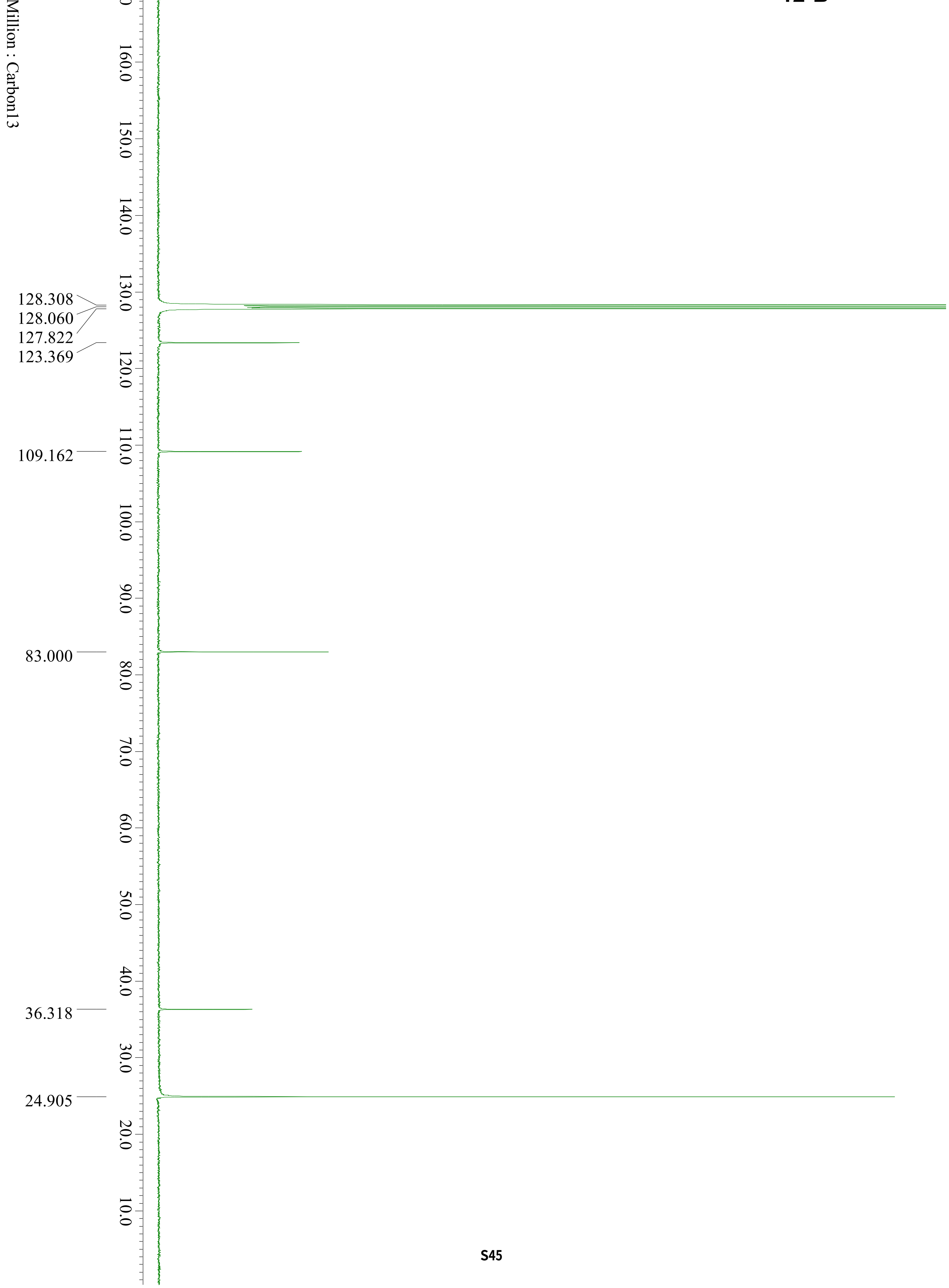

X : parts per Million : Carbon13
128.308
128.060
127.822
123.369
109.162
83.000
36.318
24.905
170.0
160.0
150.0
140.0
130.0
120.0
110.0
100.0
90.0
80.0
70.0
60.0
50.0
40.0
30.0
20.0
10.0

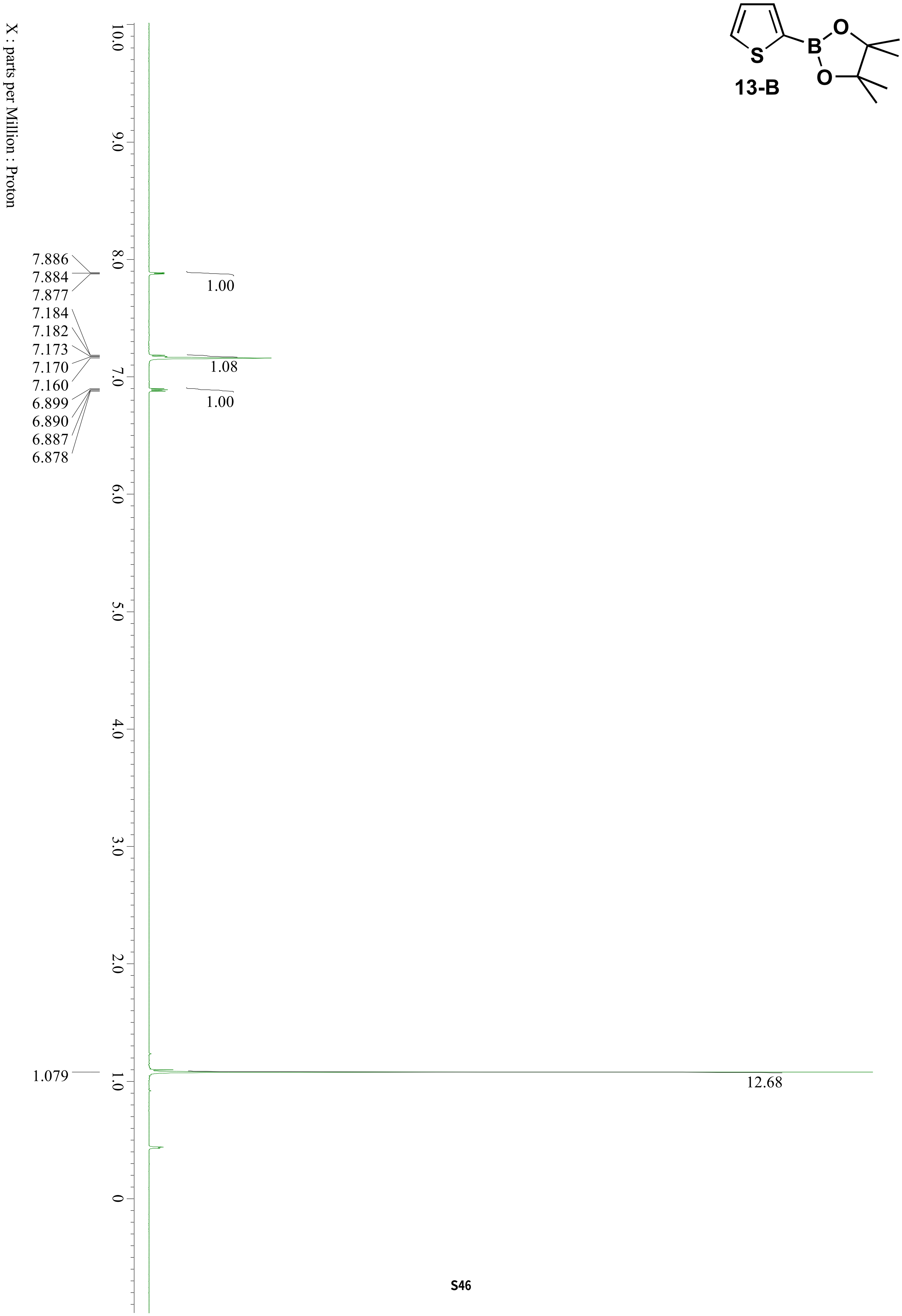
13-B
X : parts per Million : Proton
7.886
7.884
7.877
7.184
7.182
7.173
7.170
7.160
6.899
6.890
6.887
6.878
1.079
1.00
1.08
1.00
12.68
10.0
9.0
8.0
7.0
6.0
5.0
4.0
3.0
2.0
1.0
0

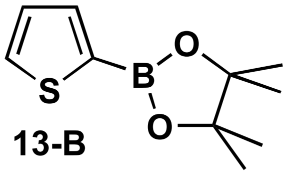

13-B

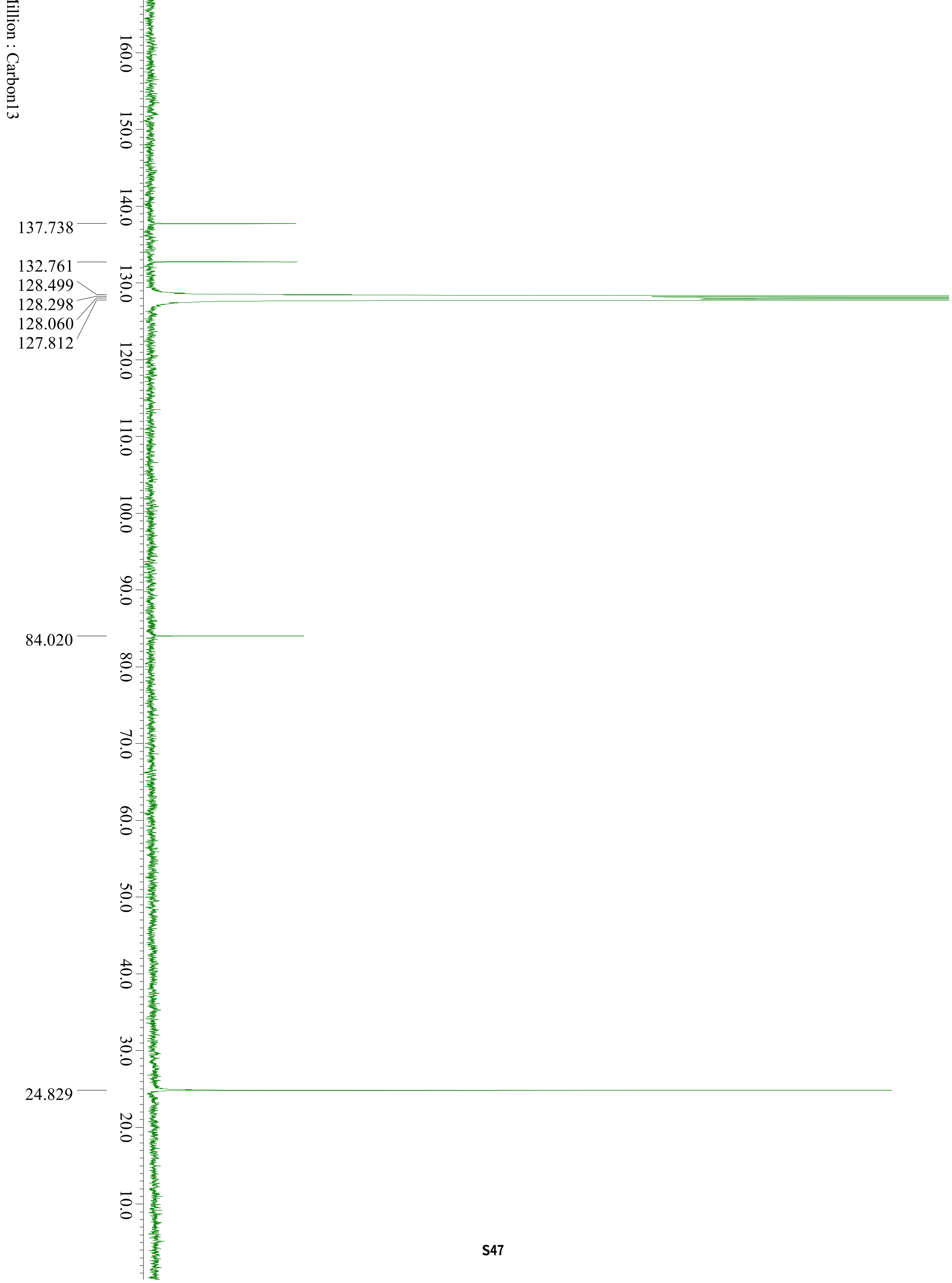
X : parts per Million : Carbon13
137.738
132.761
128.499
128.298
128.060
127.812
84.020
24.829
170.0
160.0
150.0
140.0
130.0
120.0
110.0
100.0
90.0
80.0
70.0
60.0
50.0
40.0
30.0
20.0
10.0

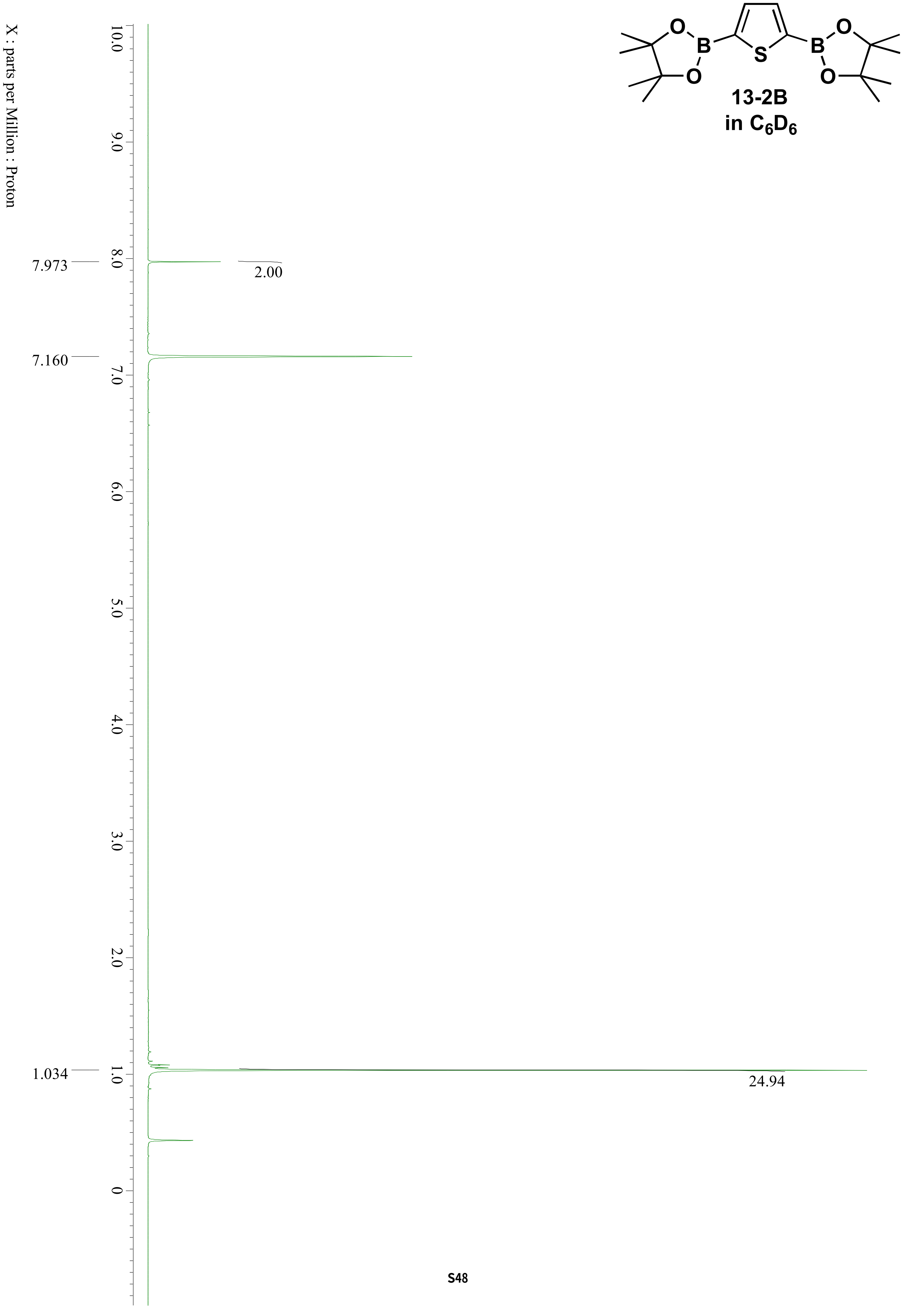
X : parts per Million : Proton
7.973
7.160
1.034
2.00
24.94
10.0
9.0
8.0
7.0
6.0
5.0
4.0
3.0
2.0
1.0
0
13-2B
in C_6D_6

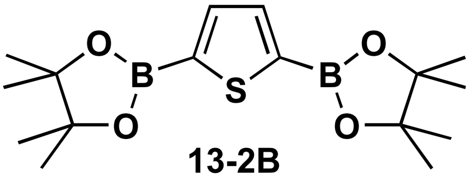

O
B
S
13-2B
in CDCl3

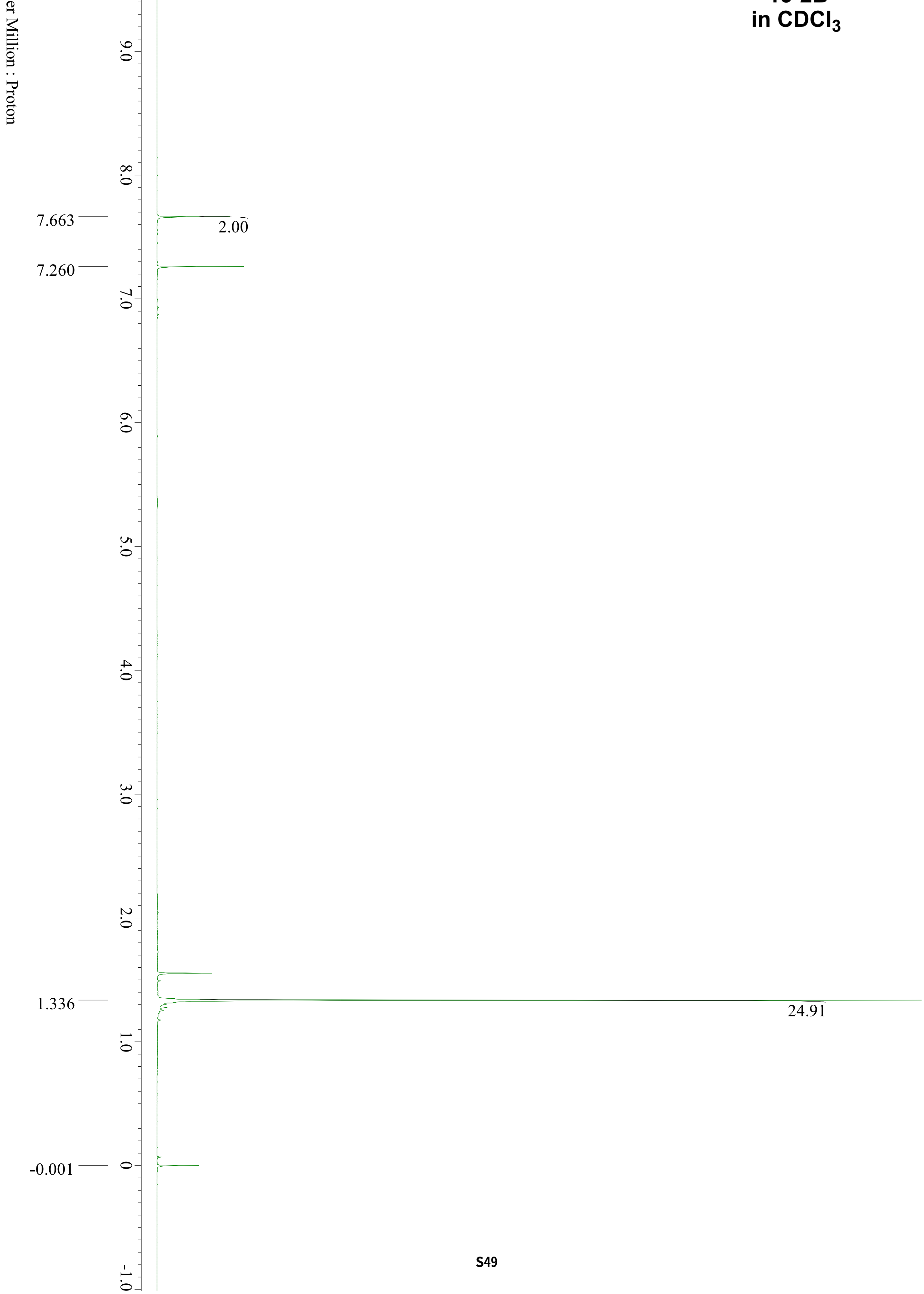

X : parts per Million : Proton
9.0
8.0
7.663
2.00
7.260
7.0
6.0
5.0
4.0
3.0
2.0
1.336
24.91
1.0
-0.001
0
-1.0

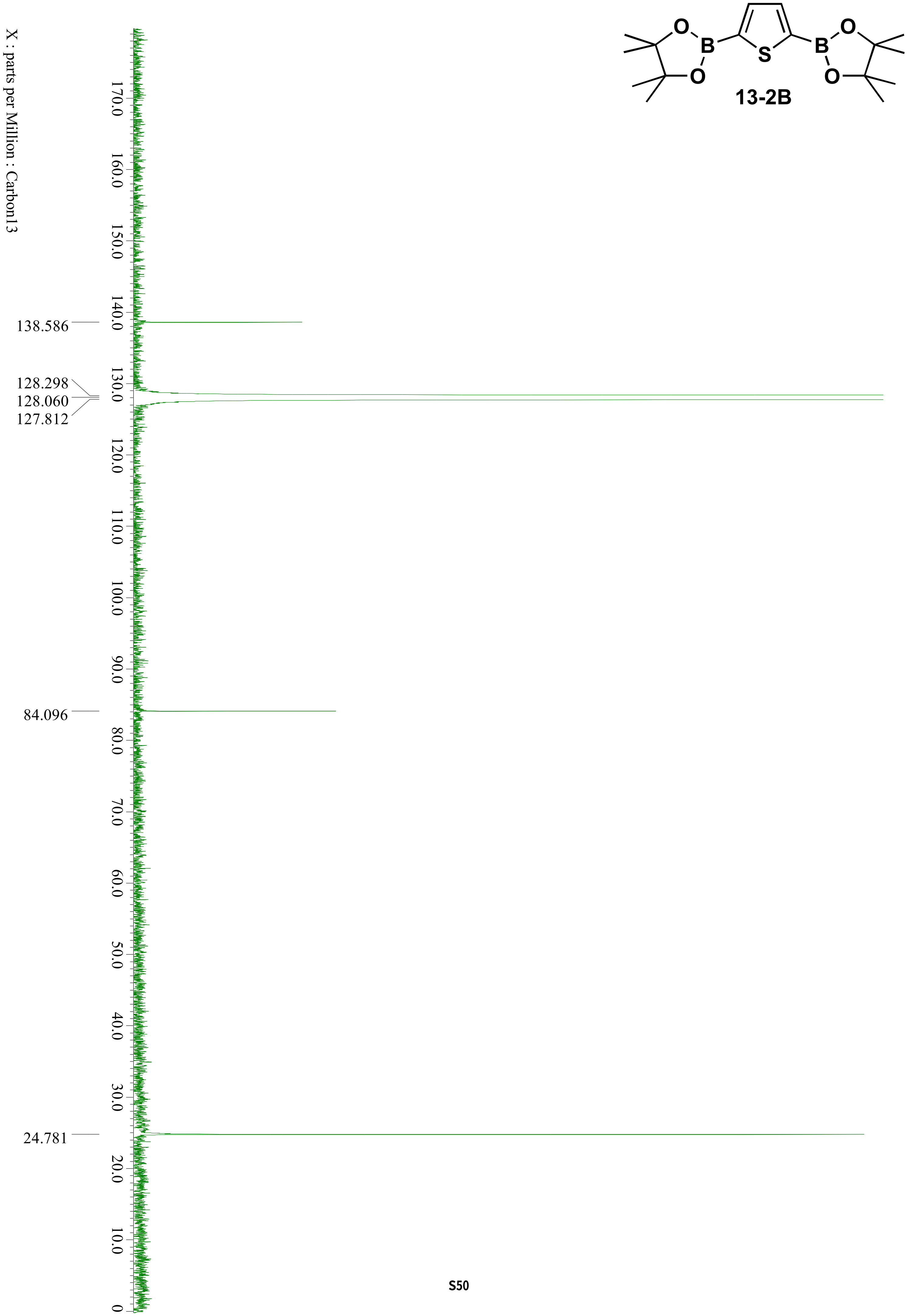
13-2B
X : parts per Million : Carbon13
138.586
128.298
128.060
127.812
84.096
24.781
170.0
160.0
150.0
140.0
130.0
120.0
110.0
100.0
90.0
80.0
70.0
60.0
50.0
40.0
30.0
20.0
10.0
0

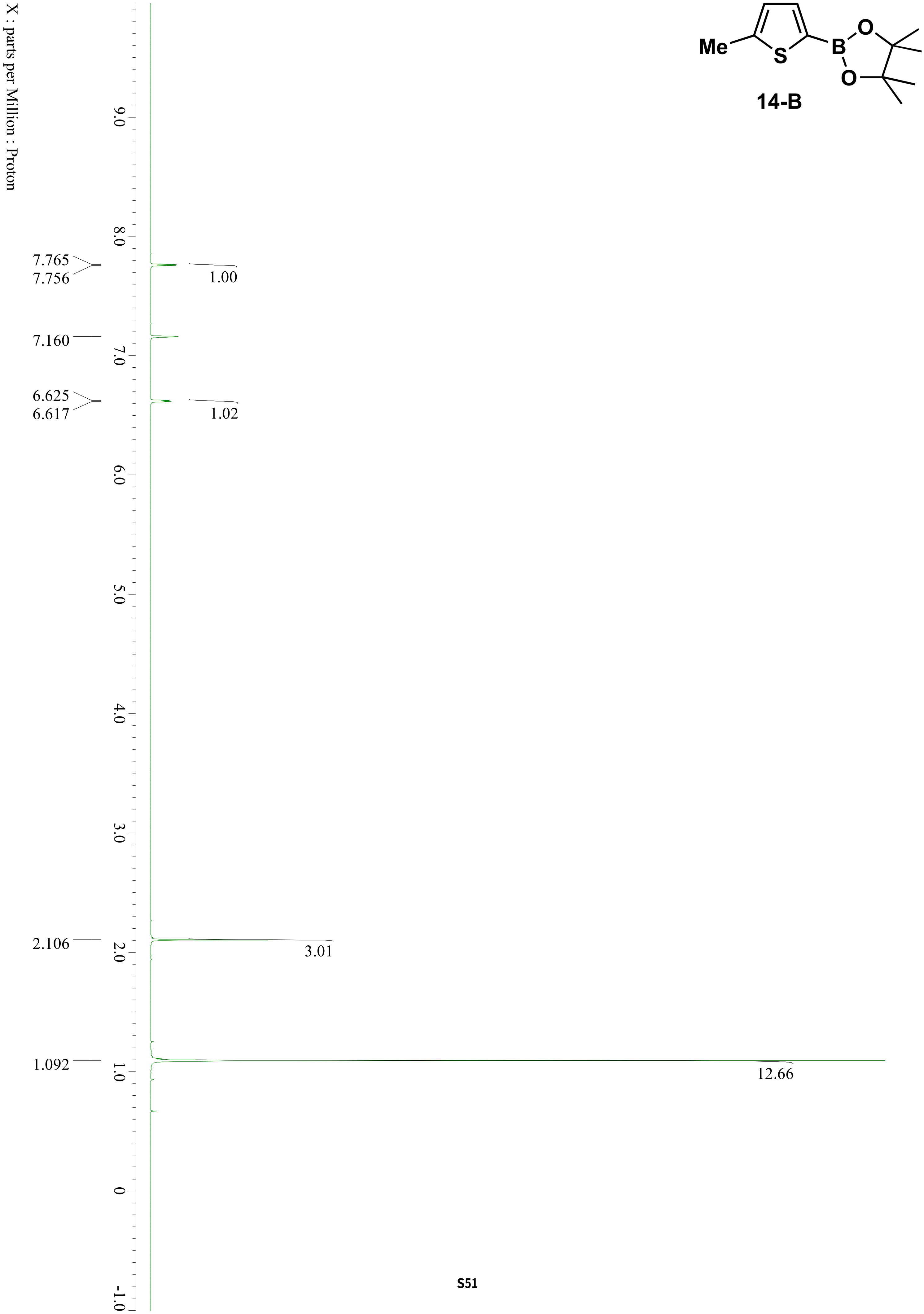
X : parts per Million : Proton
9.0
8.0
7.0
6.0
5.0
4.0
3.0
2.0
1.0
0
-1.0
7.765
7.756
7.160
6.625
6.617
2.106
1.092
1.00
1.02
3.01
12.66
Me
S
B
O
O

14-B

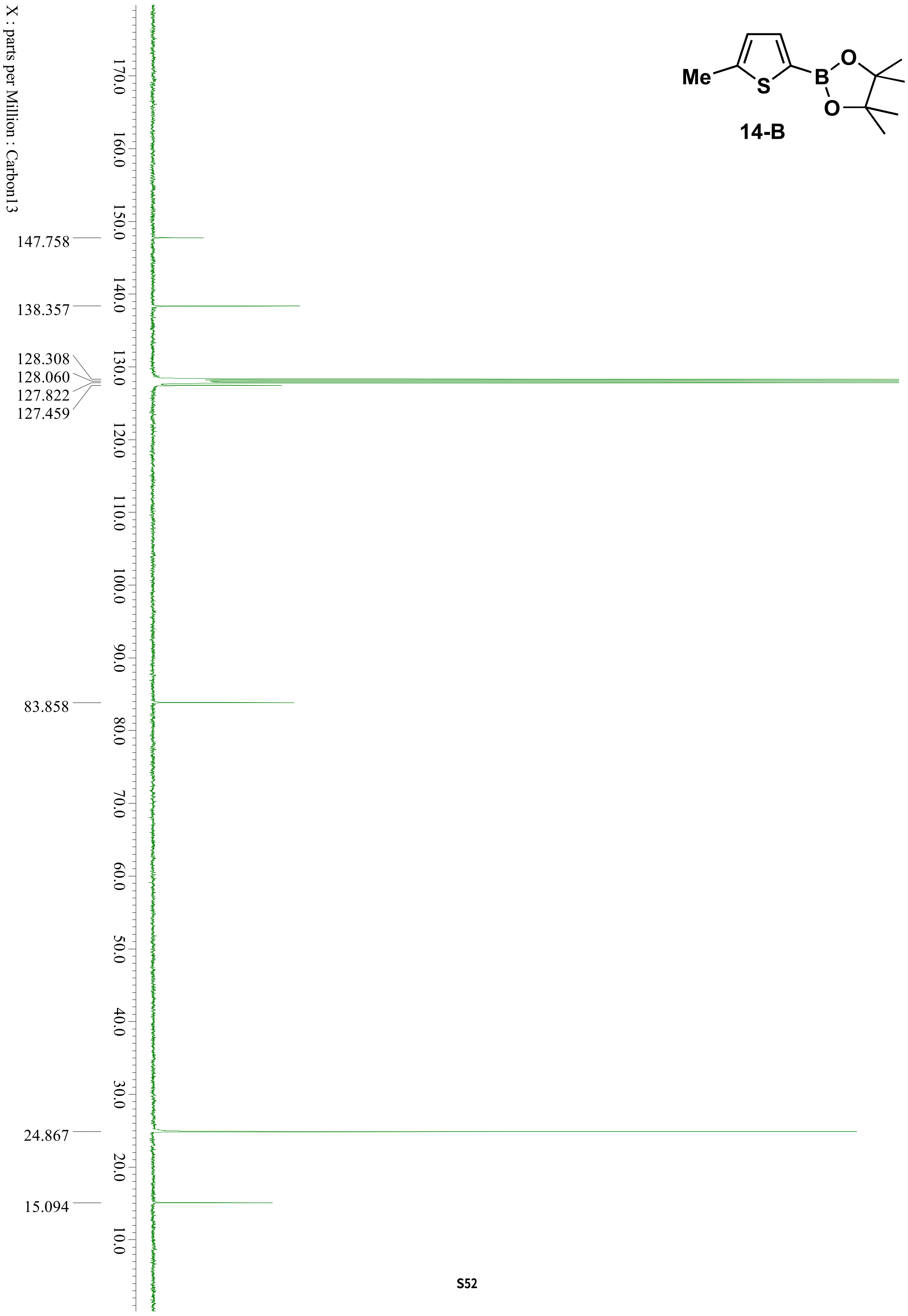

X : parts per Million : Carbon13
147.758
138.357
128.308
128.060
127.822
127.459
83.858
24.867
15.094
170.0
160.0
150.0
140.0
130.0
120.0
110.0
100.0
90.0
80.0
70.0
60.0
50.0
40.0
30.0
20.0
10.0
Me
S
B
O
O
14-B

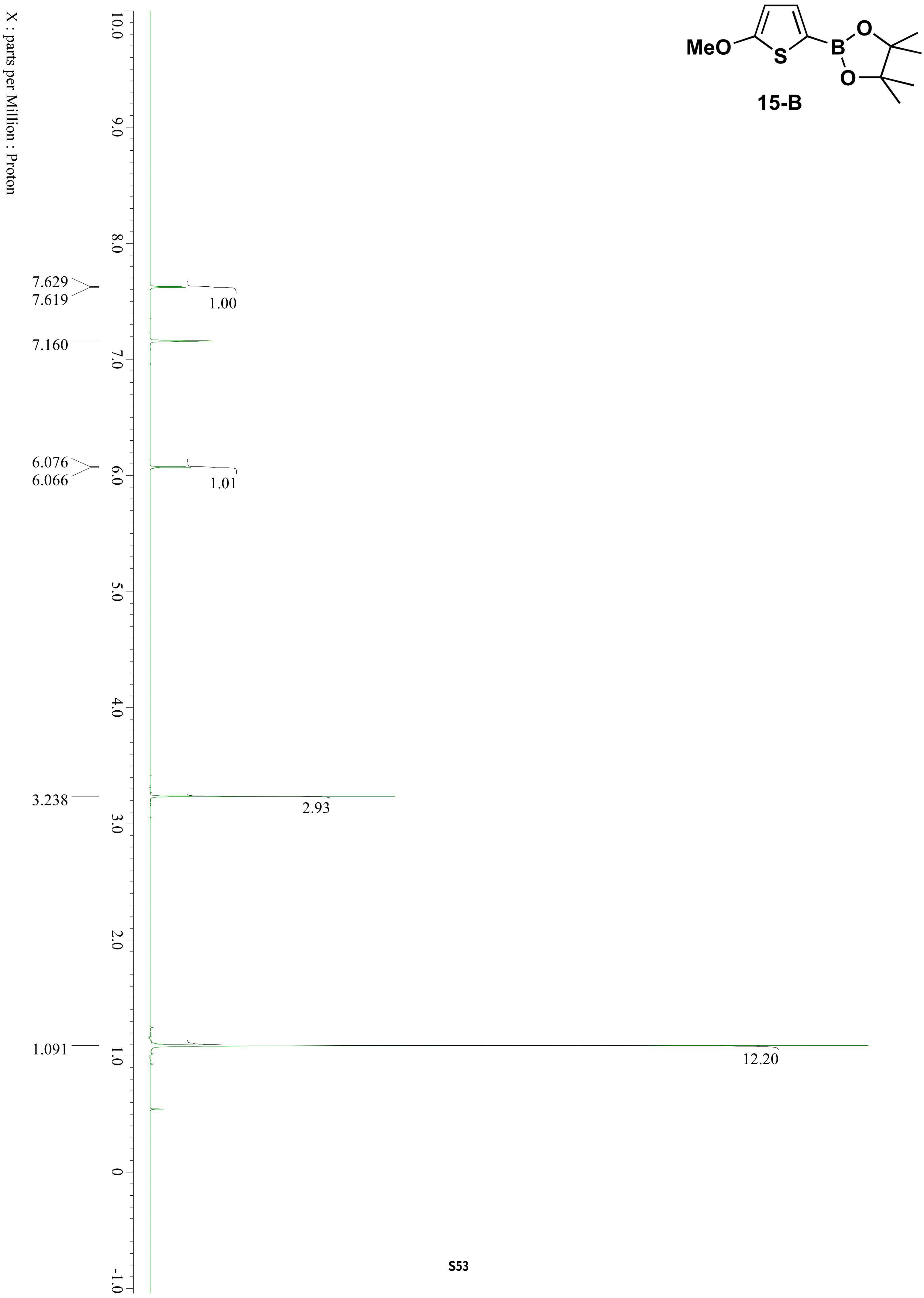
MeO
S
B
O
O
X : parts per Million : Proton
10.0
9.0
8.0
7.0
6.0
5.0
4.0
3.0
2.0
1.0
0
-1.0
7.629
7.619
7.160
6.076
6.066
3.238
1.091
1.00
1.01
2.93
12.20

15-B

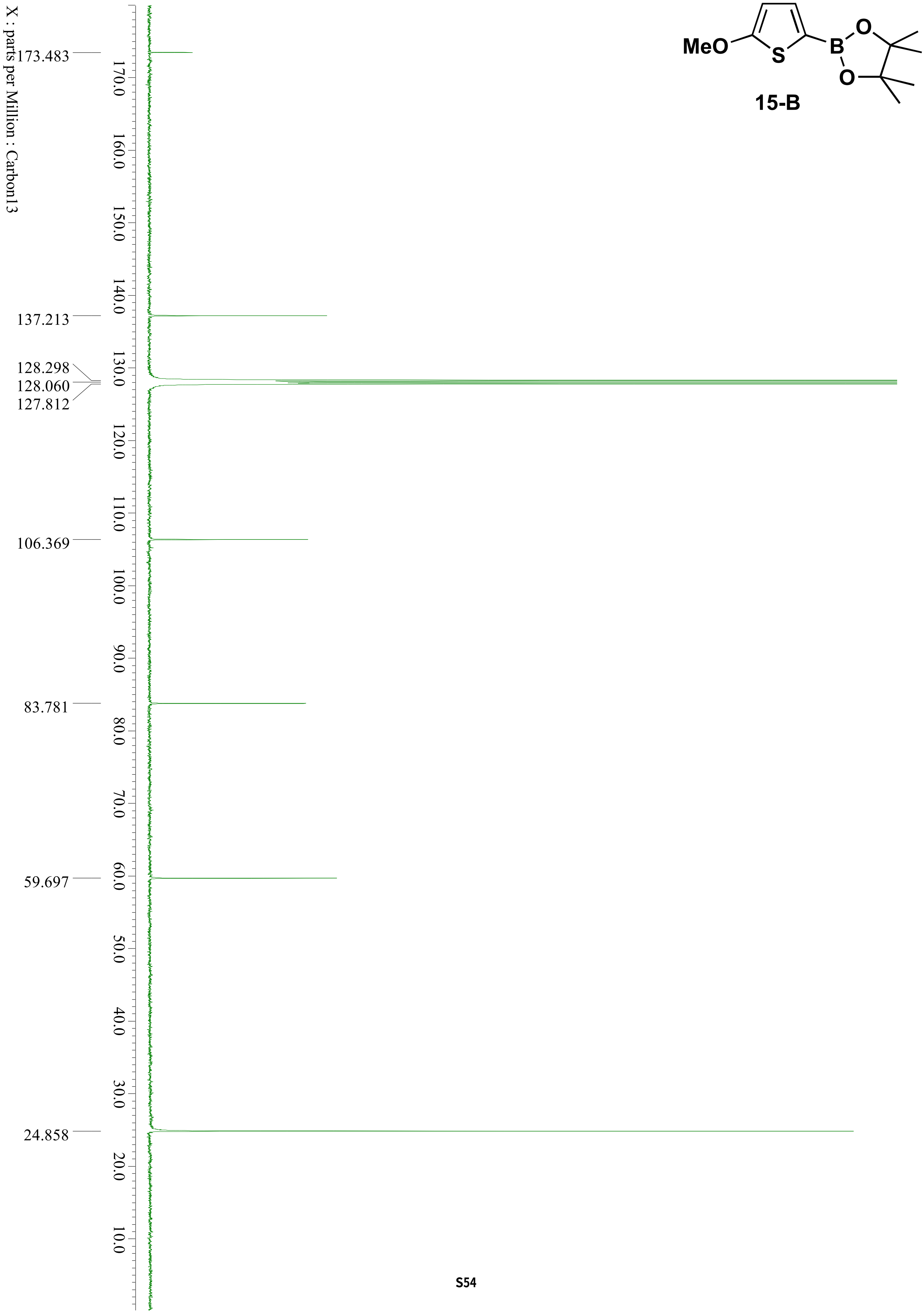
X : parts per Million : Carbon13
173.483
137.213
128.298
128.060
127.812
106.369
83.781
59.697
24.858
170.0
160.0
150.0
140.0
130.0
120.0
110.0
100.0
90.0
80.0
70.0
60.0
50.0
40.0
30.0
20.0
10.0
MeO
S
B
O
O
15-B

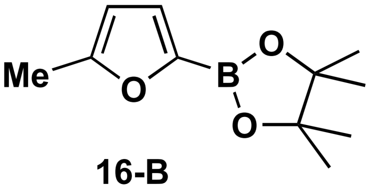
Me
O
B
O
O

16-B

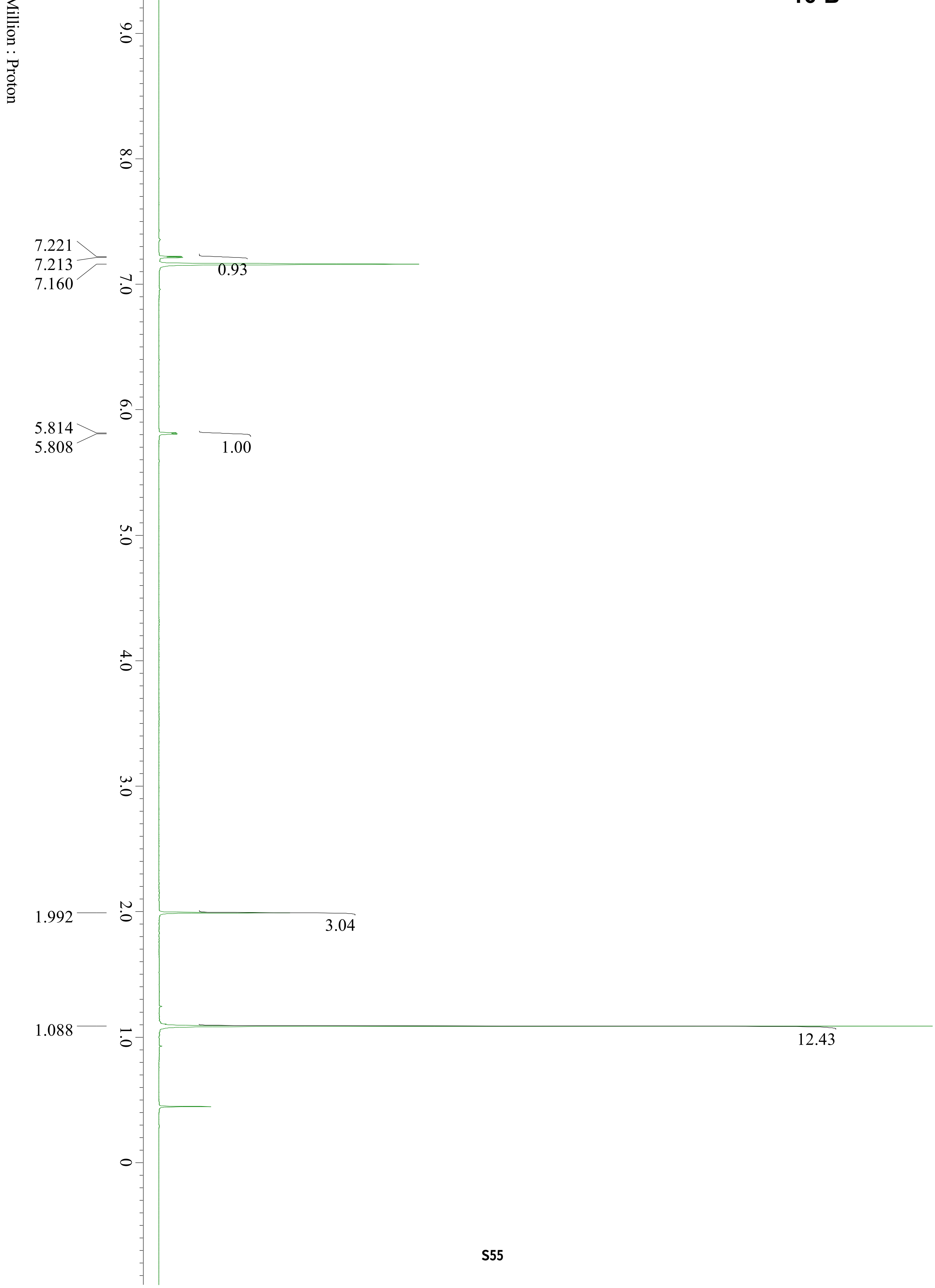
X : parts per Million : Proton
10.0
9.0
8.0
7.0
6.0
5.0
4.0
3.0
2.0
1.0
0
7.221
7.213
7.160
5.814
5.808
1.992
1.088
0.93
1.00
3.04
12.43

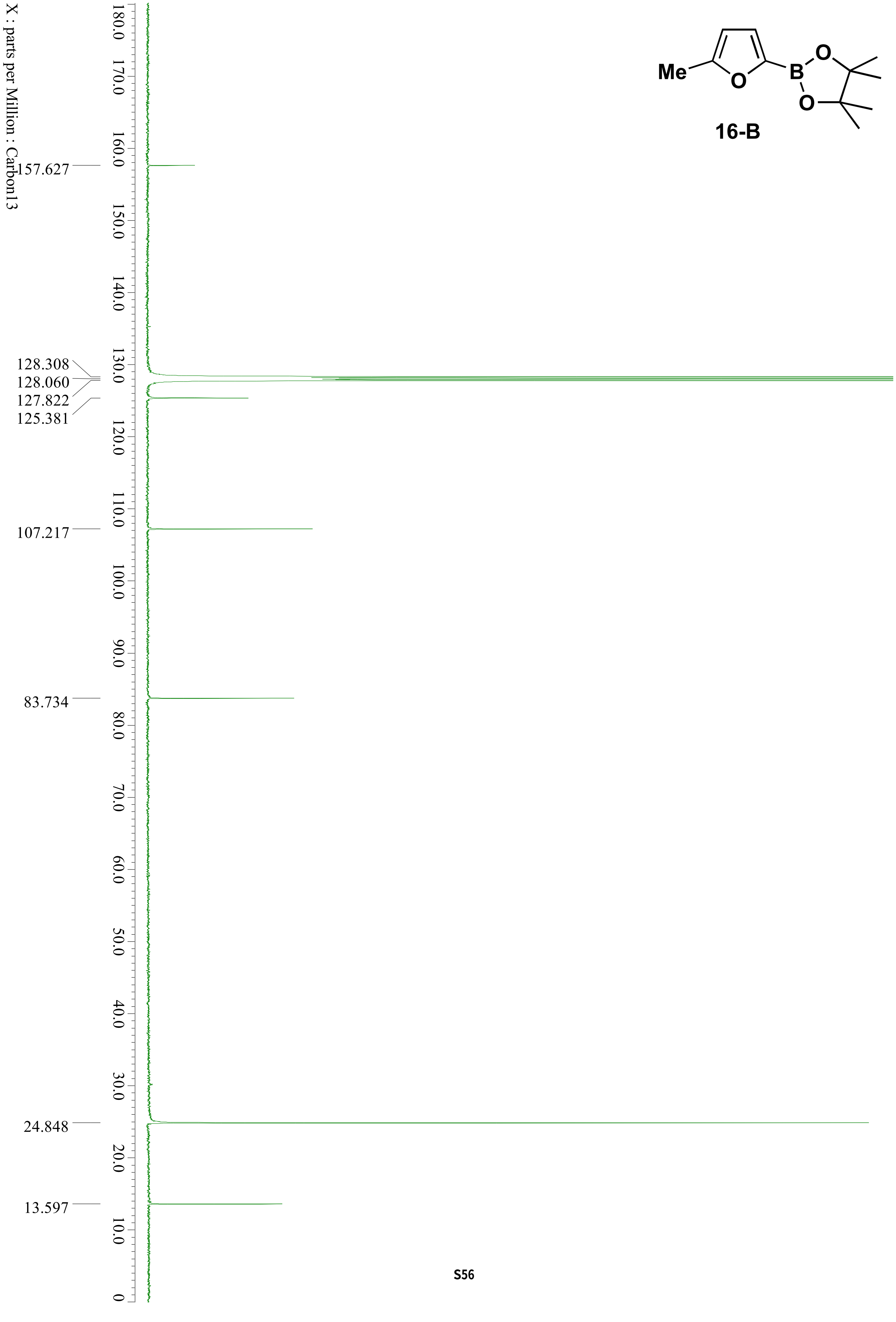

Me
O
B
O
O
16-B
X : parts per Million : Carbon13
157.627
128.308
128.060
127.822
125.381
107.217
83.734
24.848
13.597

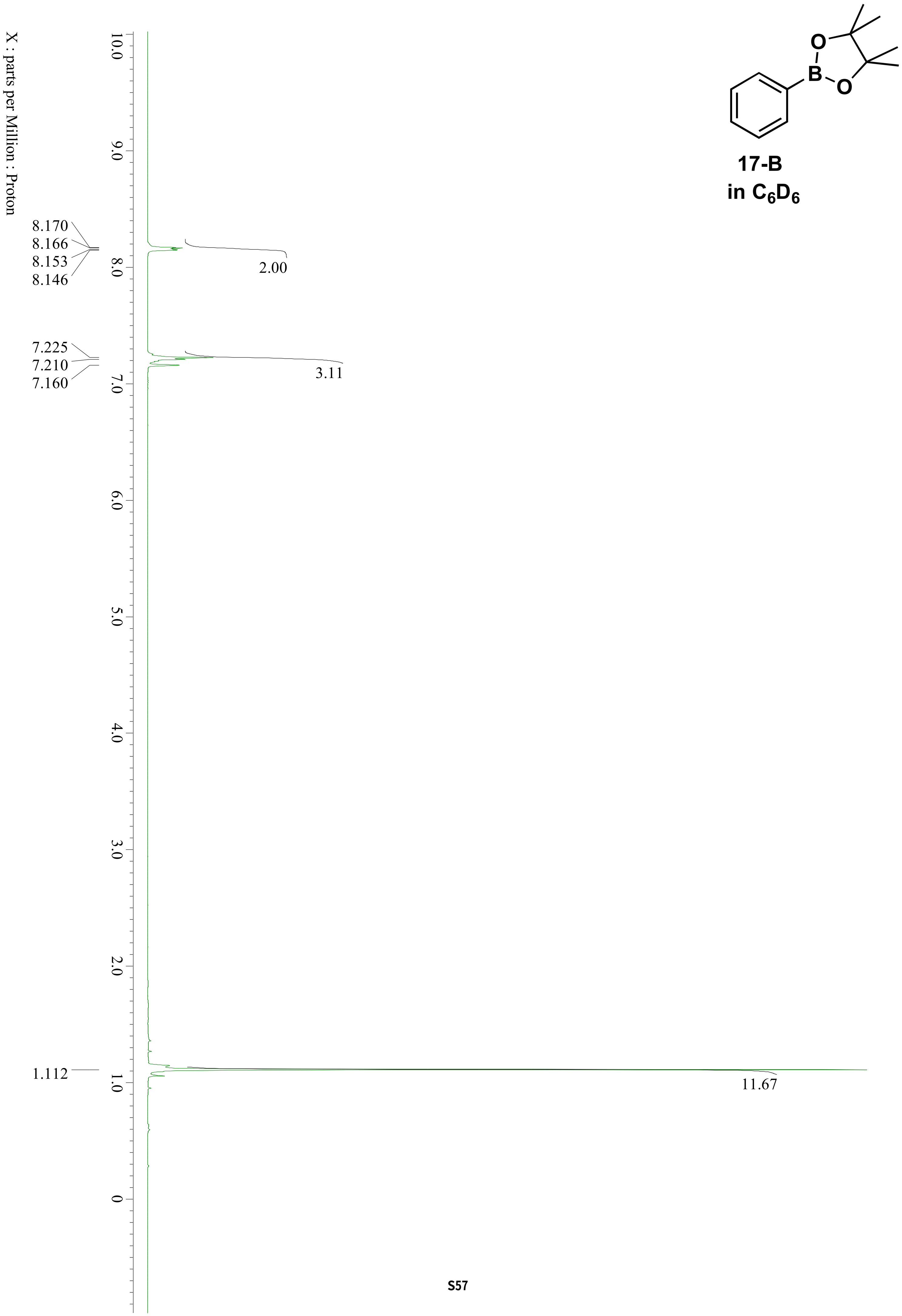

17-B
in C_6D_6

X : parts per Million : Proton
8.170
8.166
8.153
8.146
7.225
7.210
7.160
1.112
2.00
3.11
11.67
10.0
9.0
8.0
7.0
6.0
5.0
4.0
3.0
2.0
1.0
0

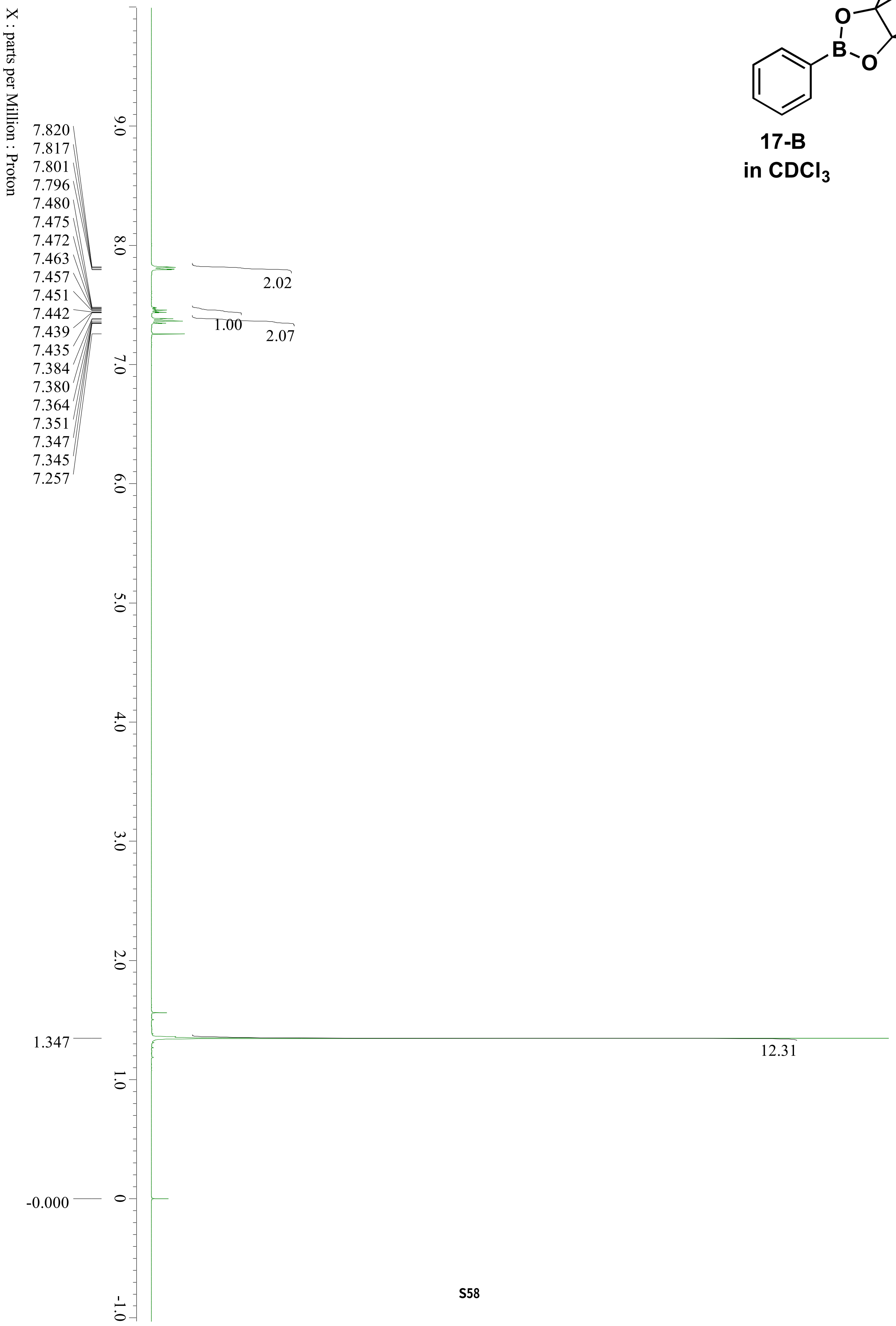
X : parts per Million : Proton
7.820
7.817
7.801
7.796
7.480
7.475
7.472
7.463
7.457
7.451
7.442
7.439
7.435
7.384
7.380
7.364
7.351
7.347
7.345
7.257
1.347
-0.000
9.0
8.0
7.0
6.0
5.0
4.0
3.0
2.0
1.0
0
-1.0
2.02
1.00
2.07
12.31

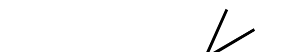

17-B
in $CDCl_3$

17-B

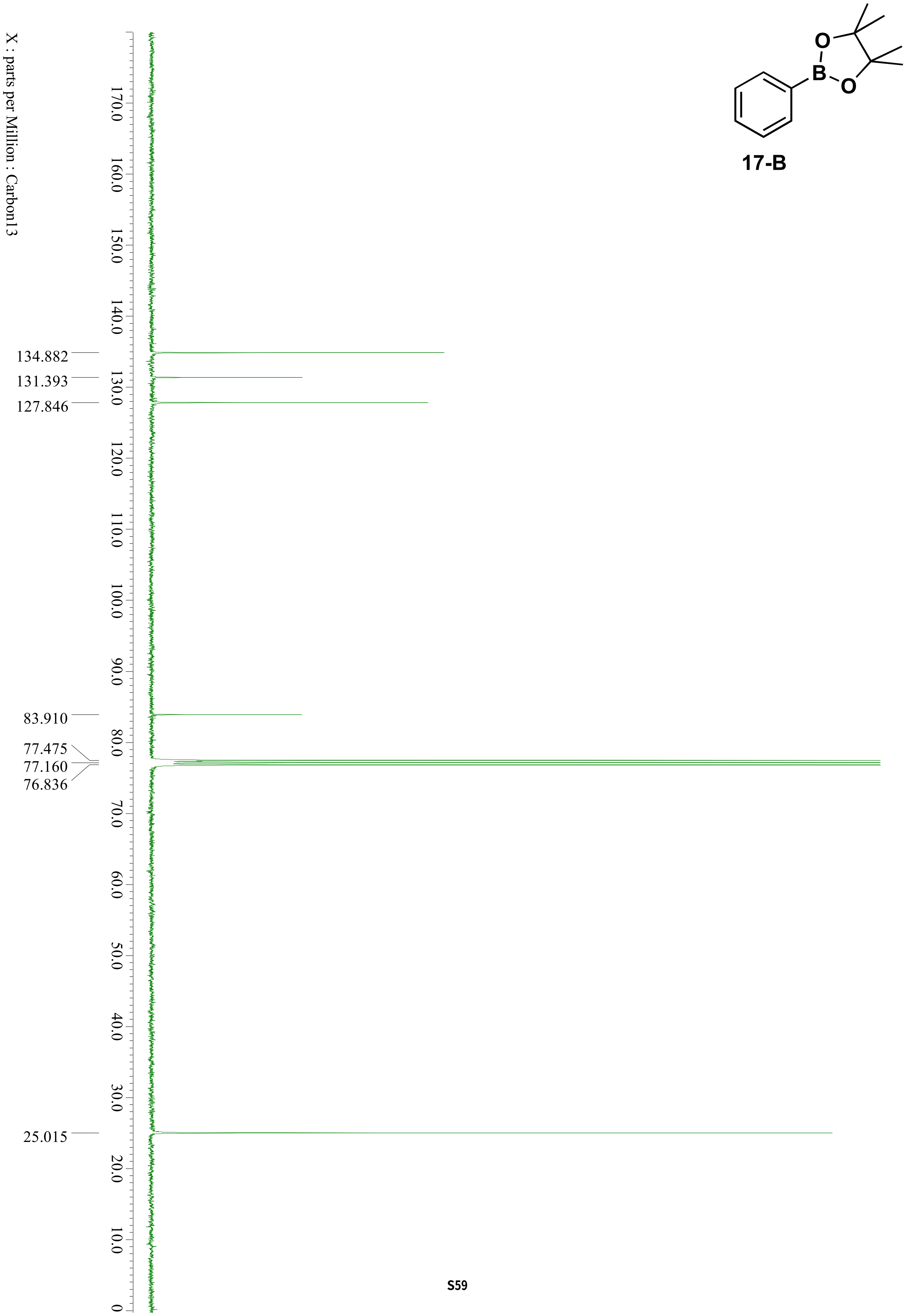
X : parts per Million : Carbon13
134.882
131.393
127.846
83.910
77.475
77.160
76.836
25.015
170.0
160.0
150.0
140.0
130.0
120.0
110.0
100.0
90.0
80.0
70.0
60.0
50.0
40.0
30.0
20.0
10.0
0

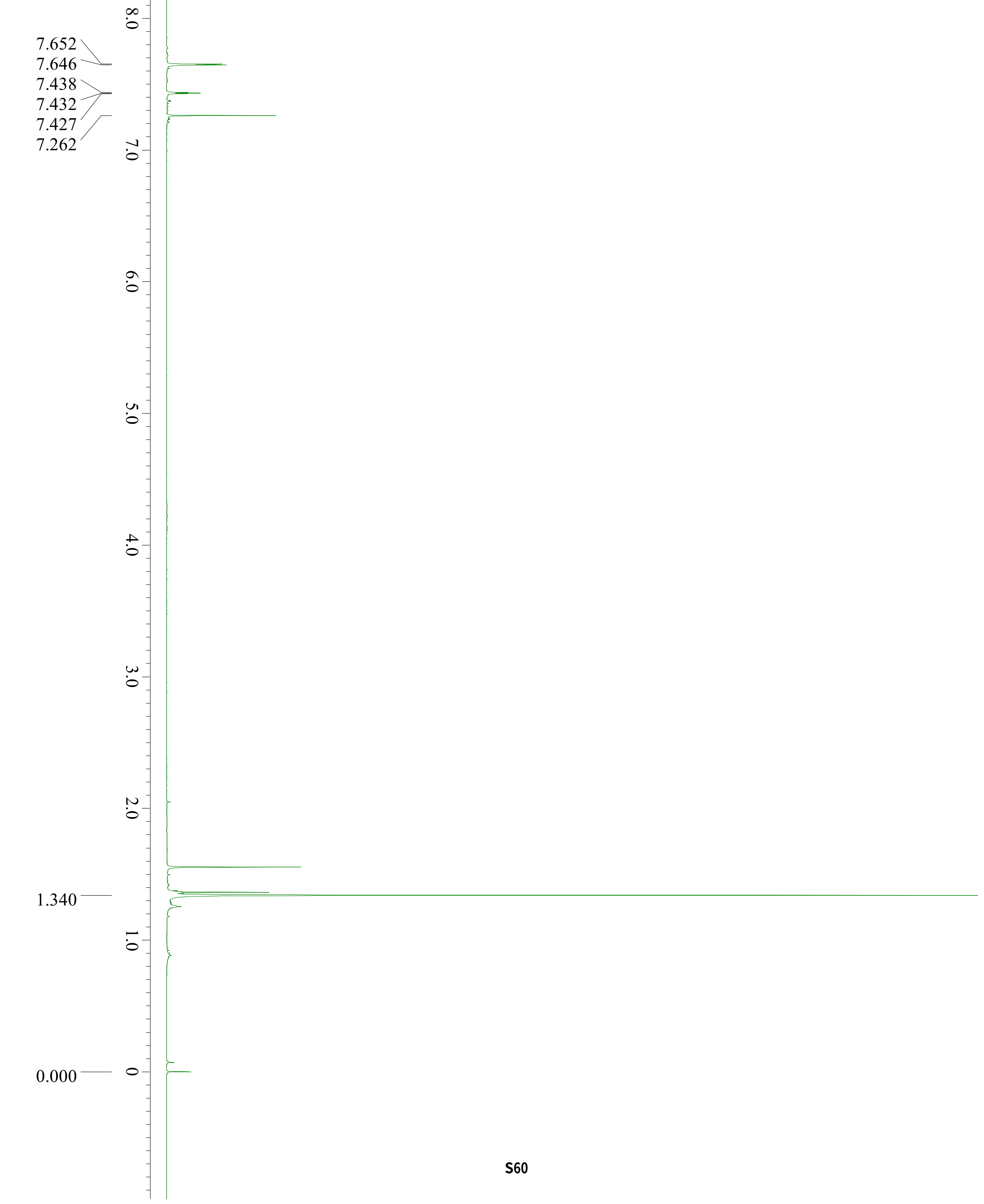
X : parts per Million : Proton
7.652
7.646
7.438
7.432
7.427
7.262
1.340
0.000
10.0
9.0
8.0
7.0
6.0
5.0
4.0
3.0
2.0
1.0
0

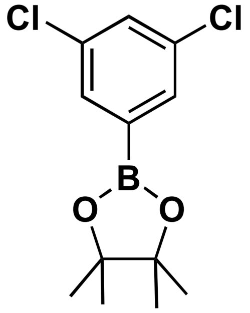

21-B

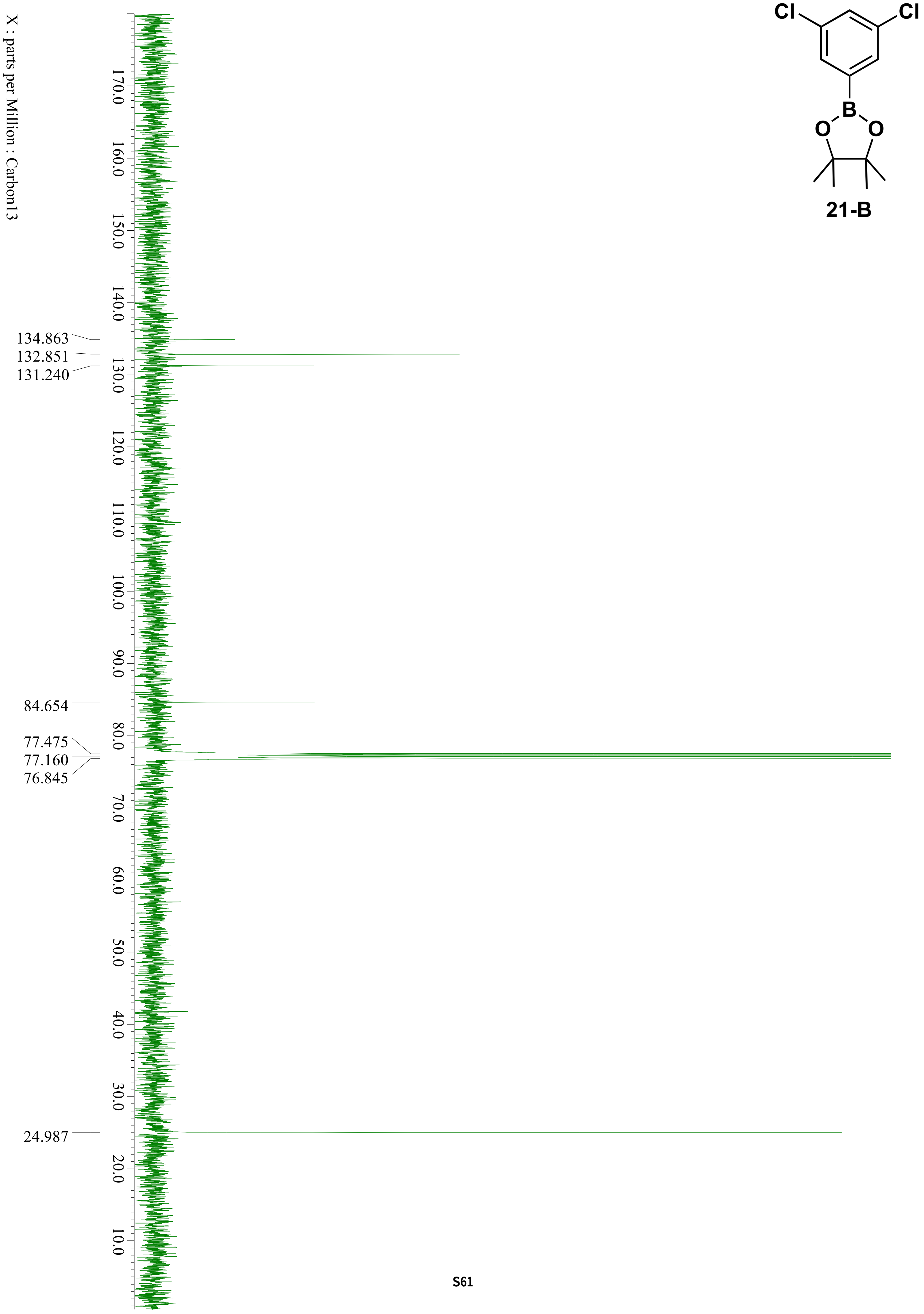
X : parts per Million : Carbon13
170.0
160.0
150.0
140.0
130.0
120.0
110.0
100.0
90.0
80.0
70.0
60.0
50.0
40.0
30.0
20.0
10.0
134.863
132.851
131.240
84.654
77.475
77.160
76.845
24.987
Cl
Cl
B
O
O
21-B

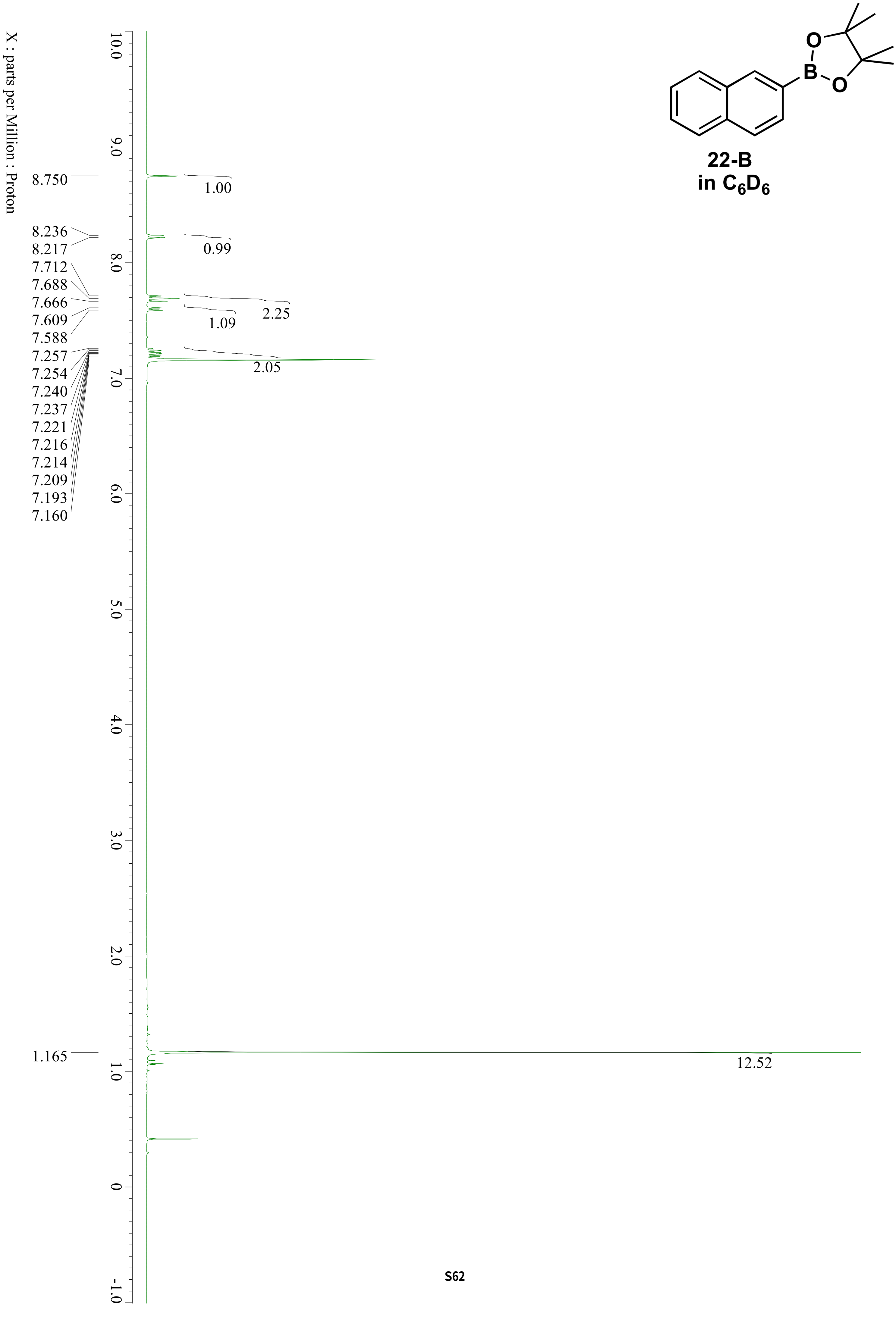
X : parts per Million : Proton
8.750
8.236
8.217
7.712
7.688
7.666
7.609
7.588
7.257
7.254
7.240
7.237
7.221
7.216
7.214
7.209
7.193
7.160
1.165
1.00
0.99
2.25
1.09
2.05
12.52
10.0
9.0
8.0
7.0
6.0
5.0
4.0
3.0
2.0
1.0
0
-1.0
O
B
O

22-B
in C_6D_6

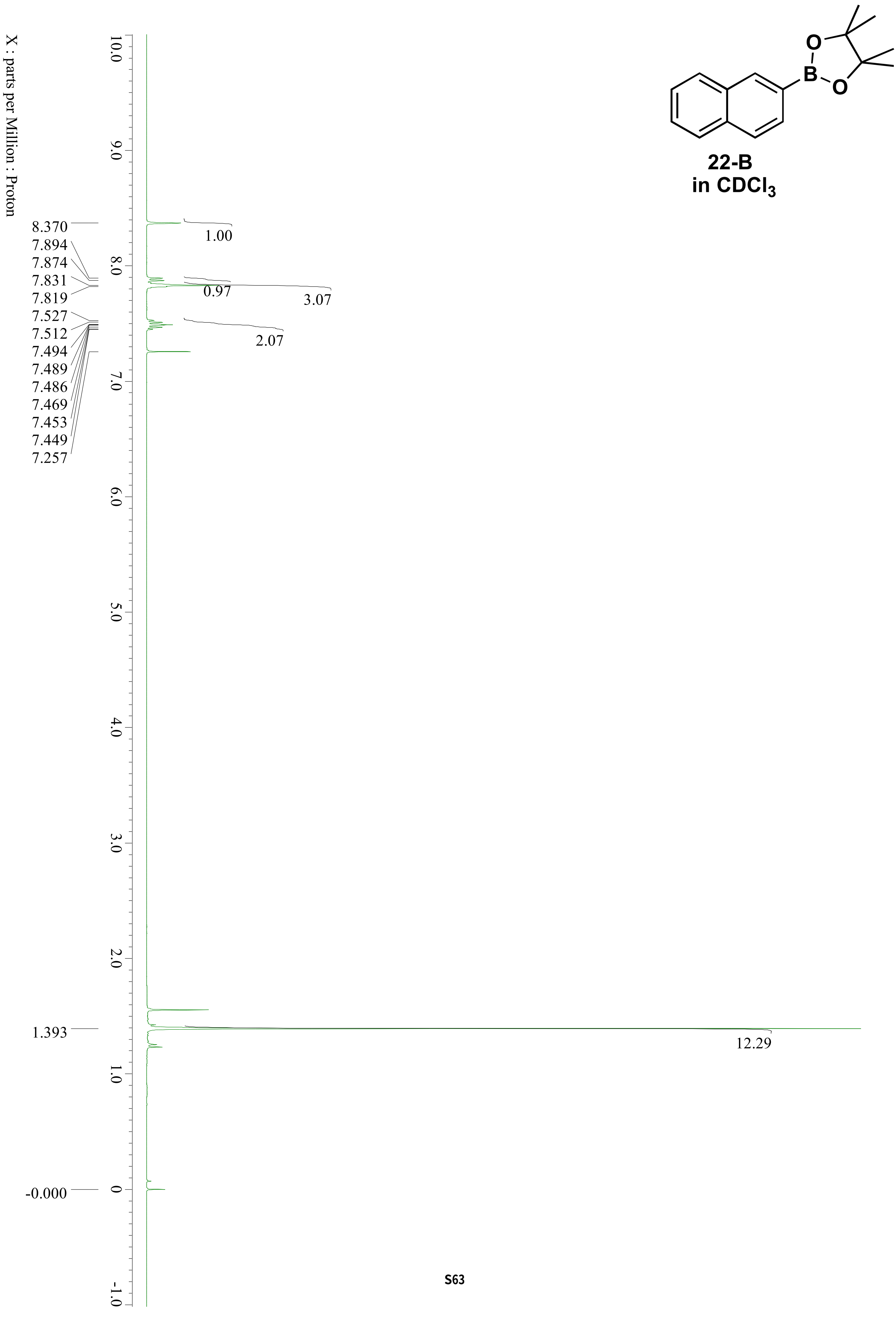
22-B
in CDCl3
X : parts per Million : Proton
10.0
9.0
8.0
7.0
6.0
5.0
4.0
3.0
2.0
1.0
0
-1.0
8.370
7.894
7.874
7.831
7.819
7.527
7.512
7.494
7.489
7.486
7.469
7.453
7.449
7.257
1.393
-0.000
1.00
0.97
3.07
2.07
12.29

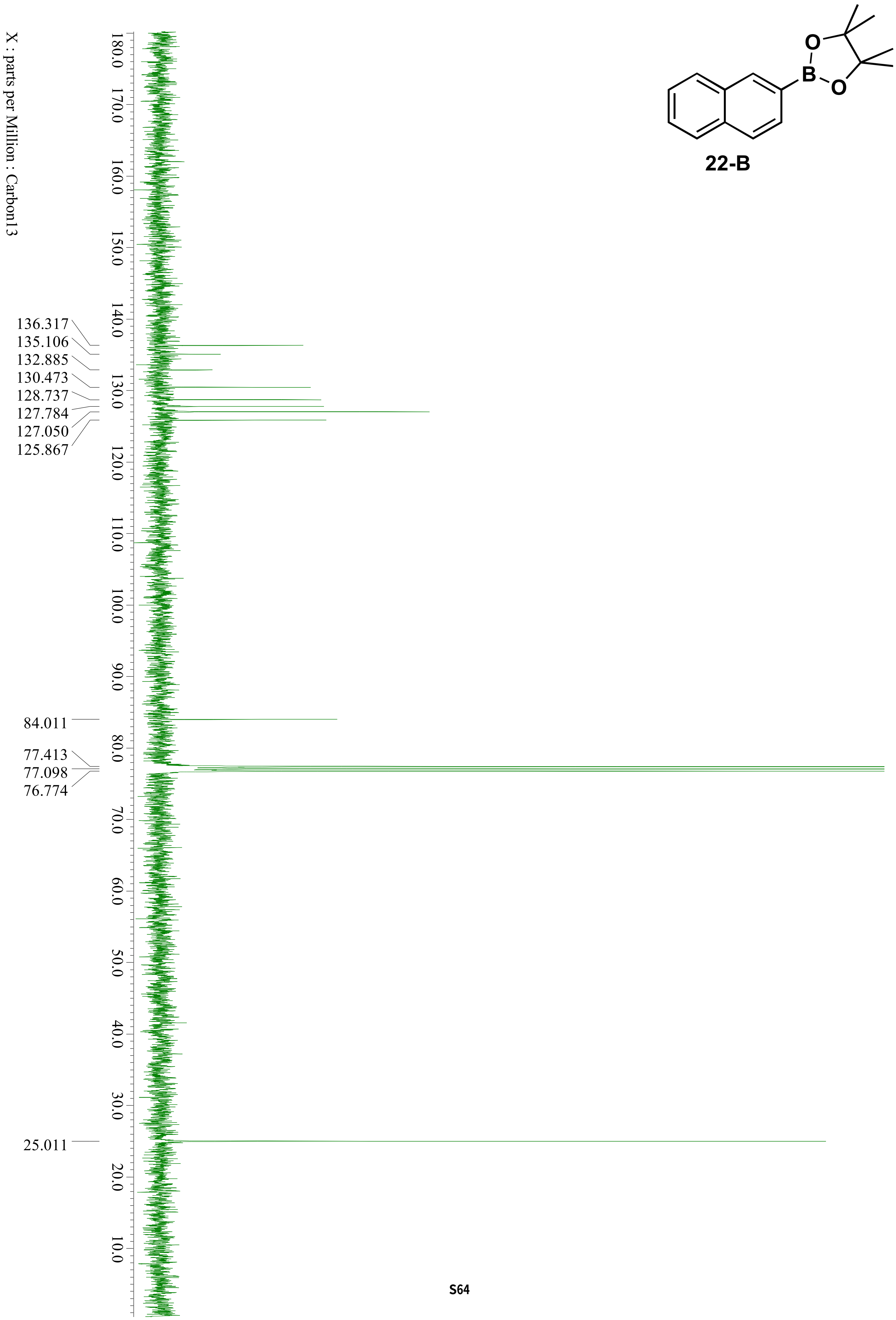

X : parts per Million : Carbon13
136.317
135.106
132.885
130.473
128.737
127.784
127.050
125.867
84.011
77.413
77.098
76.774
25.011
O
B
O

22-B

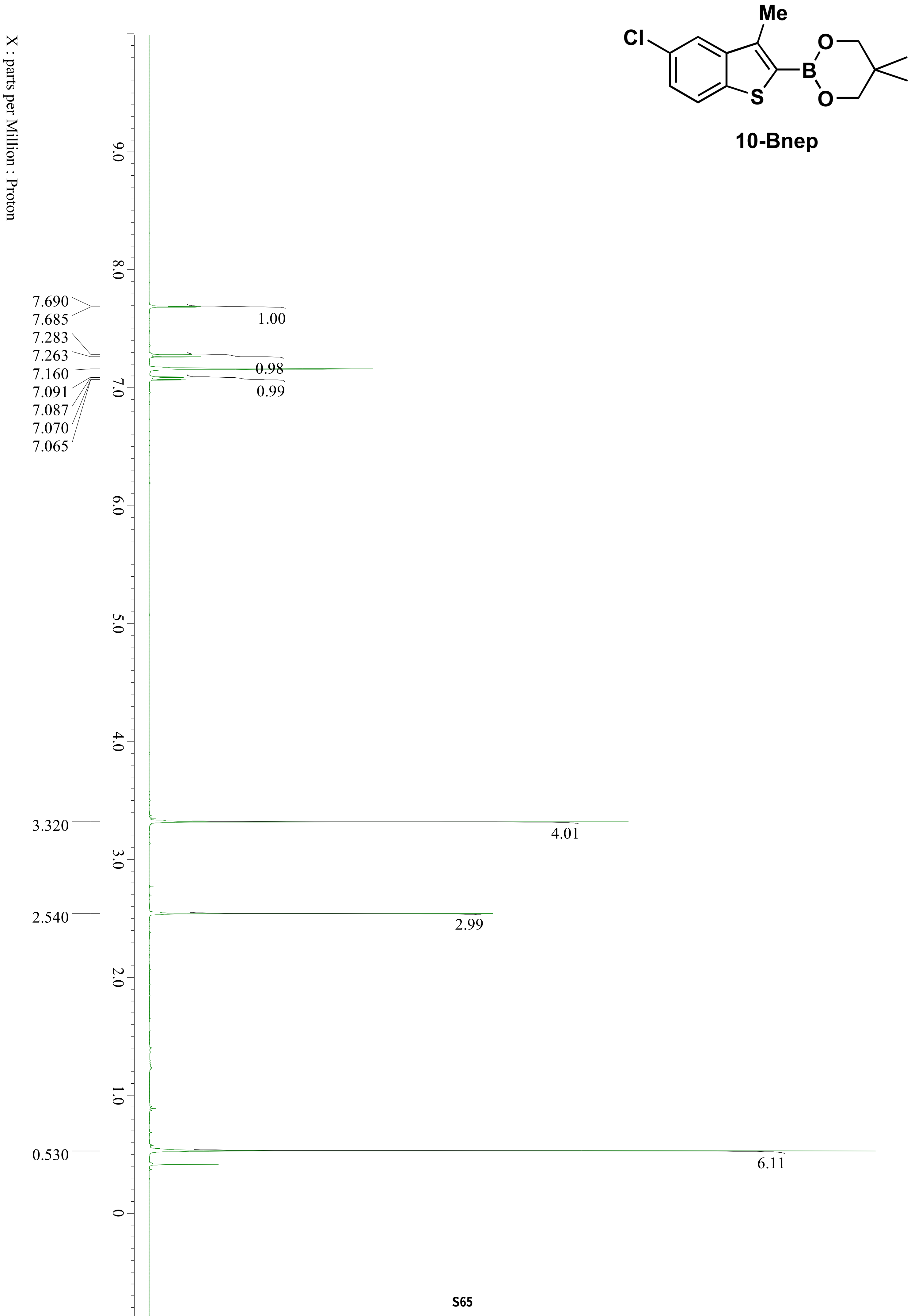

Me
Cl
S
B
O
O
10-Bnep
X : parts per Million : Proton
7.690
7.685
7.283
7.263
7.160
7.091
7.087
7.070
7.065
3.320
2.540
0.530
9.0
8.0
7.0
6.0
5.0
4.0
3.0
2.0
1.0
0
1.00
0.98
0.99
4.01
2.99
6.11

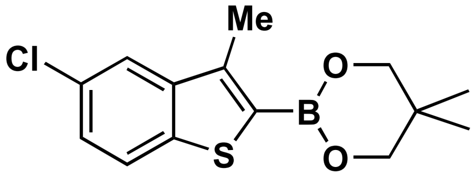
Me
Cl
O
B
O
S

10-Bnep

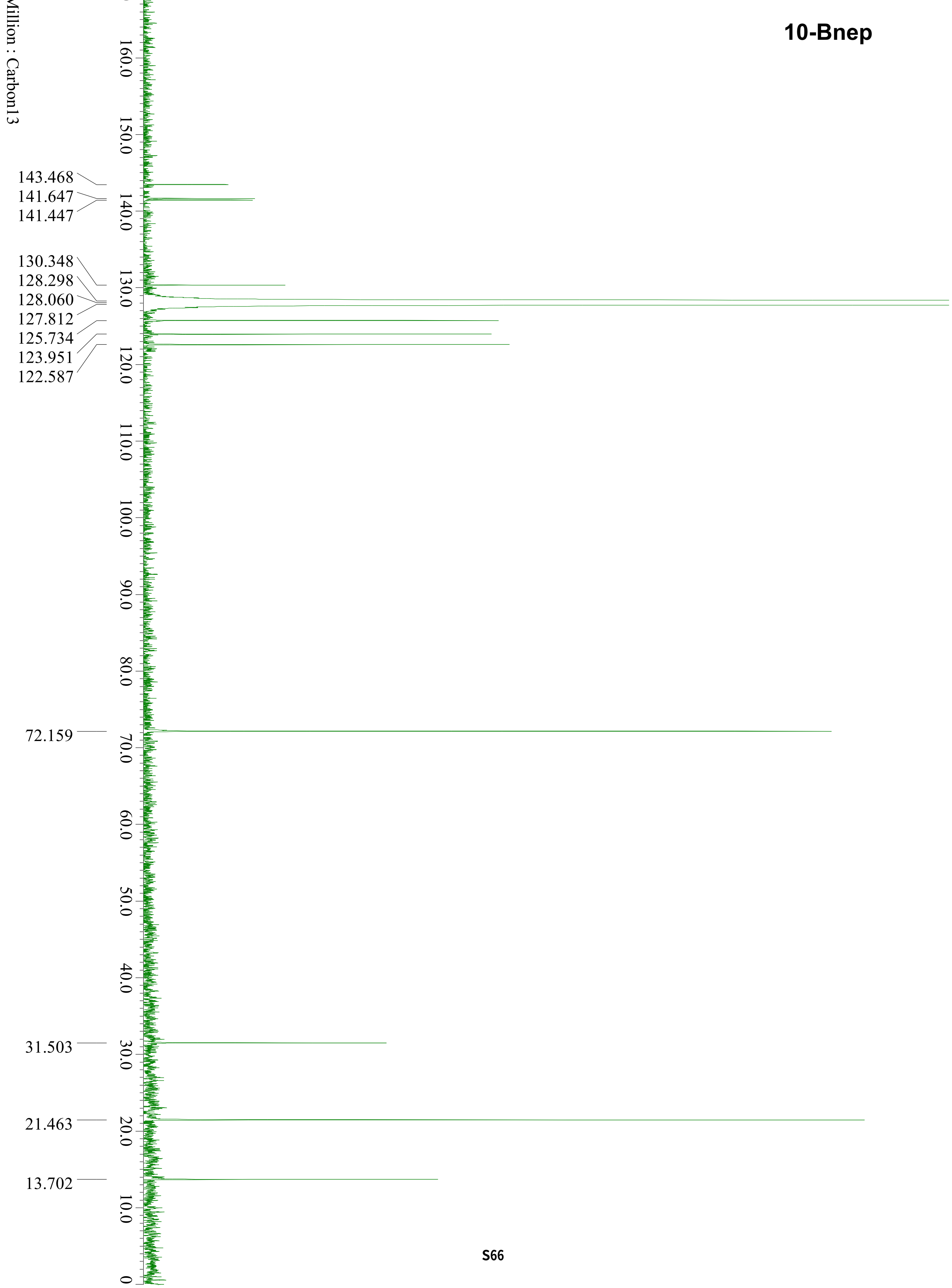
X : parts per Million : Carbon13
143.468
141.647
141.447
130.348
128.298
128.060
127.812
125.734
123.951
122.587
72.159
31.503
21.463
13.702
170.0
160.0
150.0
140.0
130.0
120.0
110.0
100.0
90.0
80.0
70.0
60.0
50.0
40.0
30.0
20.0
10.0
0

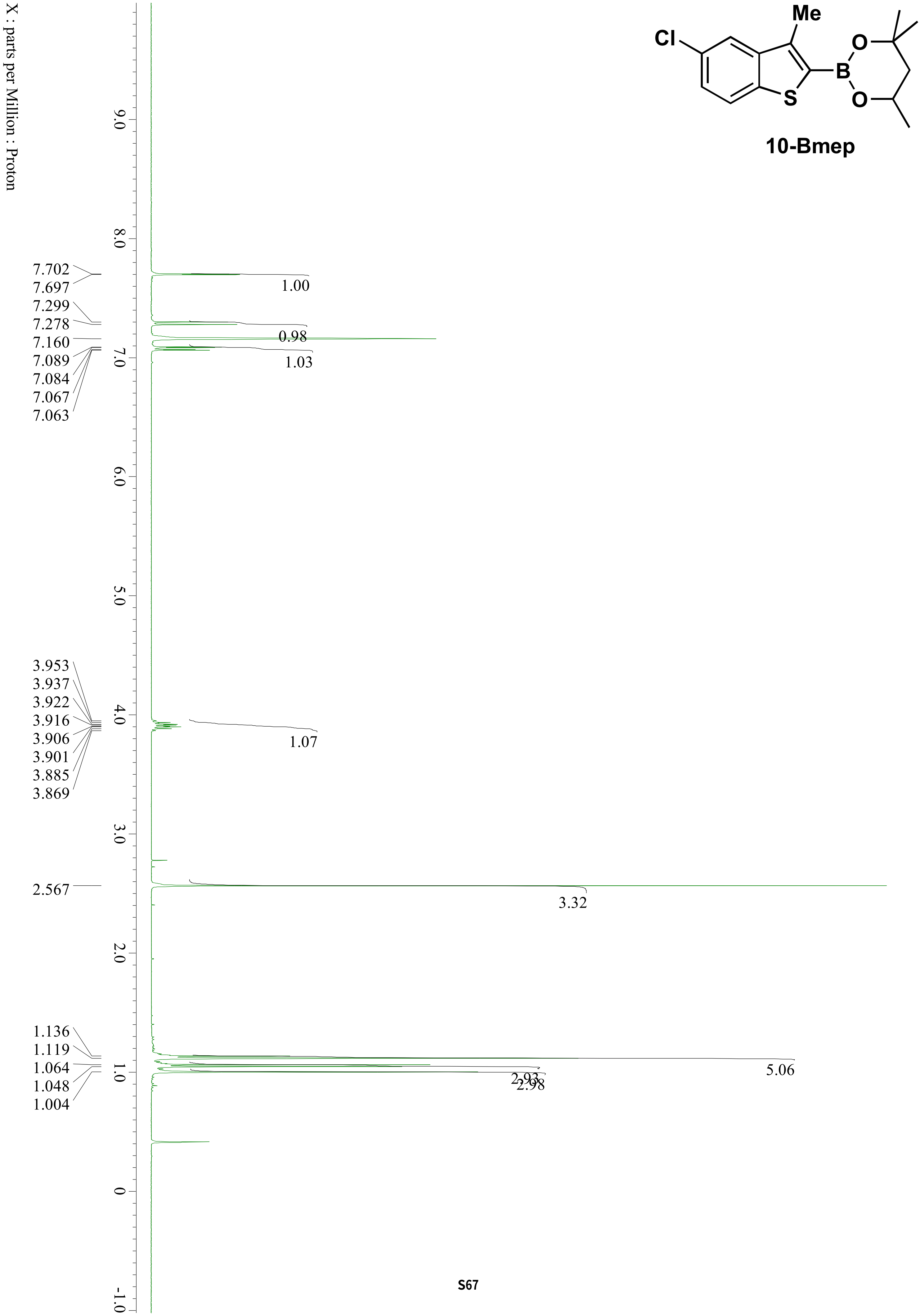
X : parts per Million : Proton
9.0
8.0
7.0
6.0
5.0
4.0
3.0
2.0
1.0
0
-1.0
7.702
7.697
7.299
7.278
7.160
7.089
7.084
7.067
7.063
3.953
3.937
3.922
3.916
3.906
3.901
3.885
3.869
2.567
1.136
1.119
1.064
1.048
1.004
1.00
0.98
1.03
1.07
3.32
5.06
2.93
2.98
Me
Cl
S
B
O
O

10-Bmep

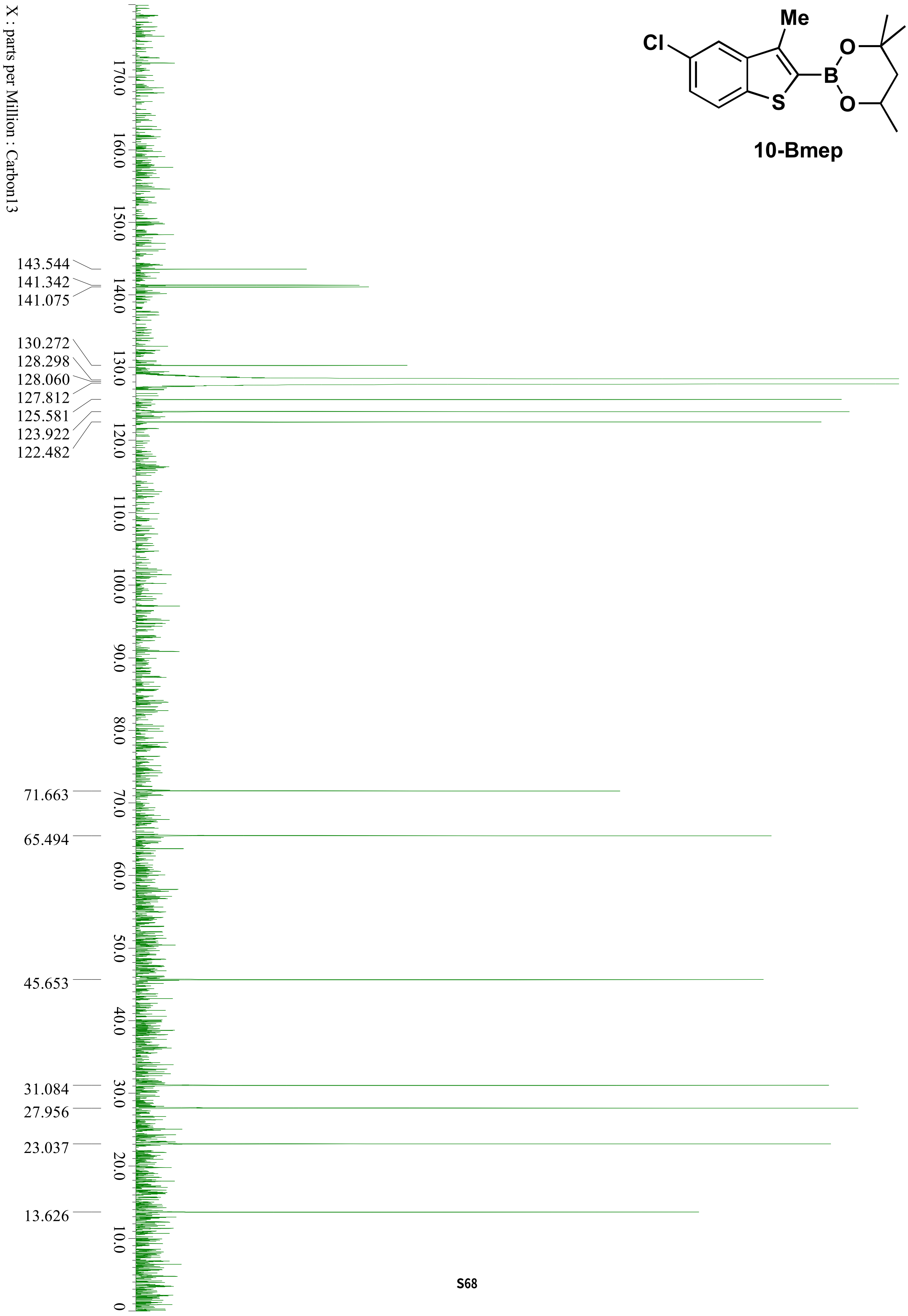
Me
Cl
S
B
O
O

10-Bmep

X : parts per Million : Carbon13
143.544
141.342
141.075
130.272
128.298
128.060
127.812
125.581
123.922
122.482
71.663
65.494
45.653
31.084
27.956
23.037
13.626
170.0
160.0
150.0
140.0
130.0
120.0
110.0
100.0
90.0
80.0
70.0
60.0
50.0
40.0
30.0
20.0
10.0
0

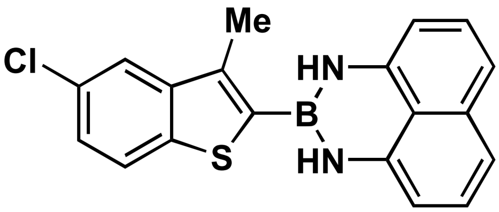

10-Bdan

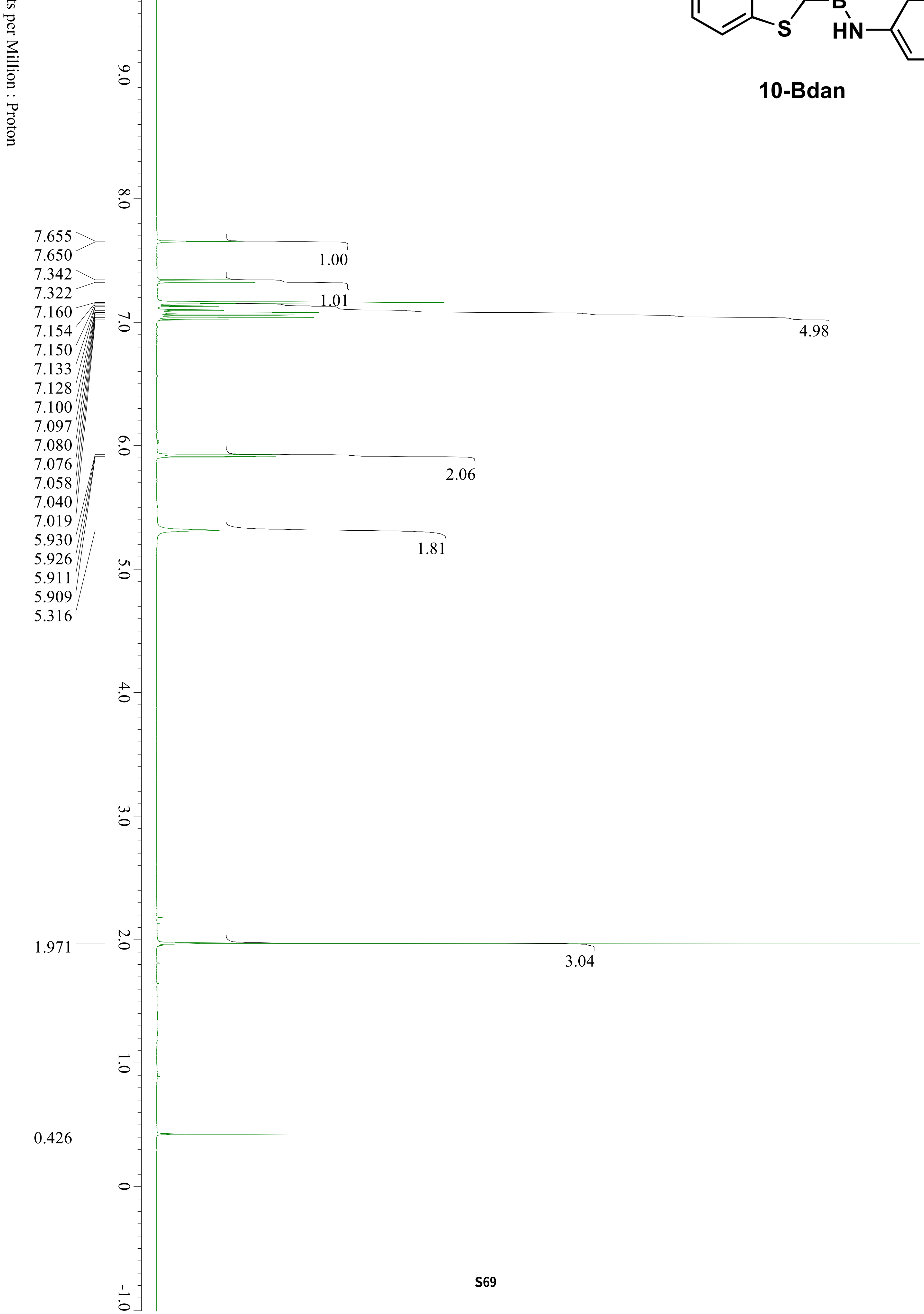
X : parts per Million : Proton
7.655
7.650
7.342
7.322
7.160
7.154
7.150
7.133
7.128
7.100
7.097
7.080
7.076
7.058
7.040
7.019
5.930
5.926
5.911
5.909
5.316
1.971
0.426
1.00
1.01
4.98
2.06
1.81
3.04
10.0
9.0
8.0
7.0
6.0
5.0
4.0
3.0
2.0
1.0
0
-1.0

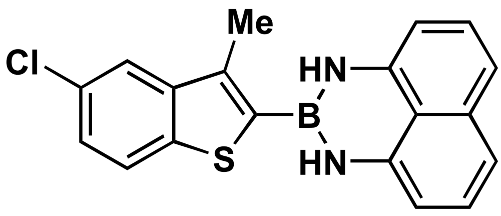
Me
Cl
HN
B
S
HN

10-Bdan

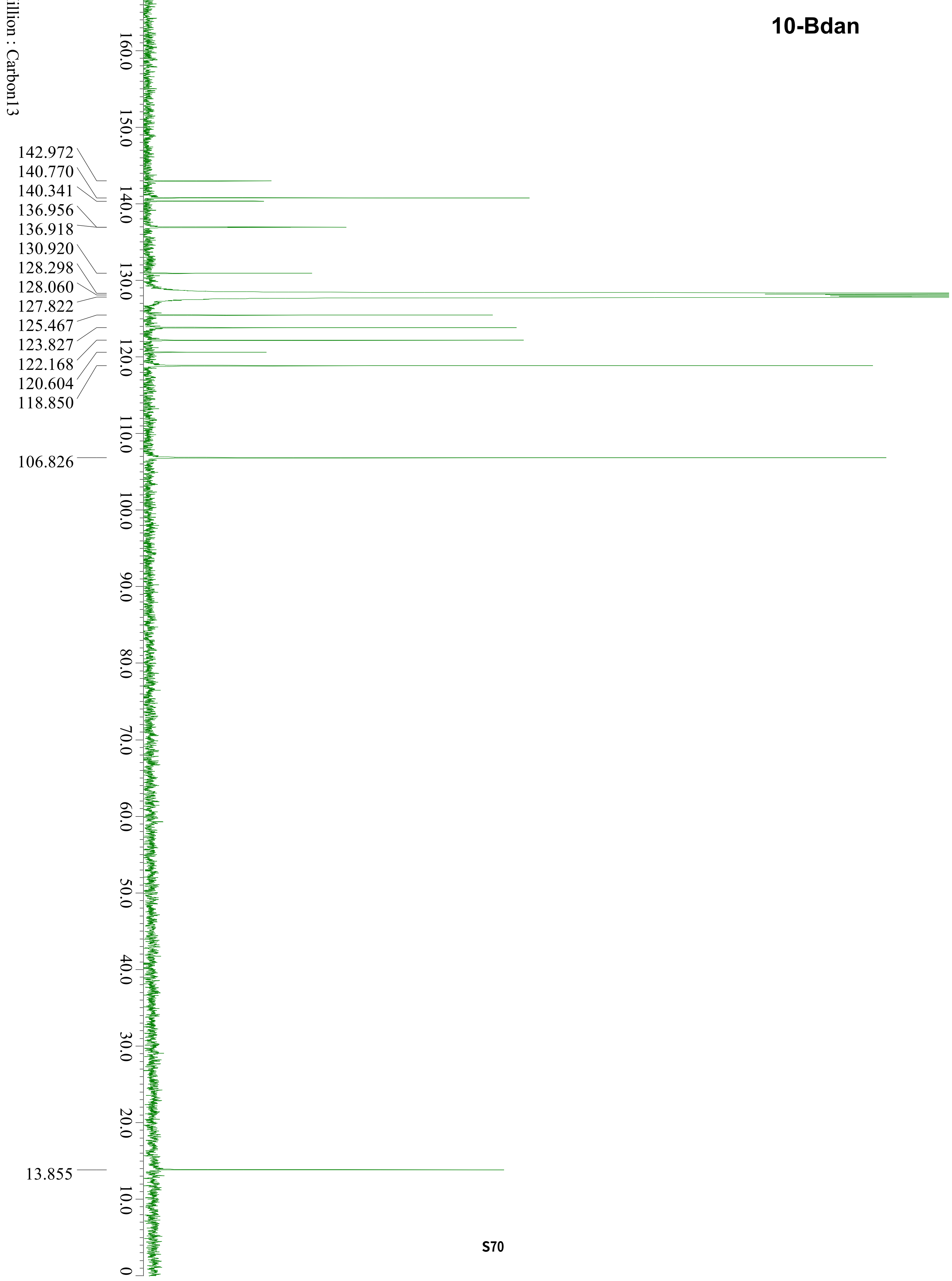
X : parts per Million : Carbon13
142.972
140.770
140.341
136.956
136.918
130.920
128.298
128.060
127.822
125.467
123.827
122.168
120.604
118.850
106.826
13.855
180.0
170.0
160.0
150.0
140.0
130.0
120.0
110.0
100.0
90.0
80.0
70.0
60.0
50.0
40.0
30.0
20.0
10.0
0
